# Psychiatric consultation in general practitioners’ daily practice: a qualitative study on the experience of consultation-liaison psychiatry interventions in primary care settings in French-speaking Switzerland

**DOI:** 10.1186/s12875-022-01937-y

**Published:** 2022-12-07

**Authors:** Konstantinos Tzartzas, Pierre-Nicolas Oberhauser, Régis Marion-Veyron, Stéphane Saillant

**Affiliations:** 1 Department of Ambulatory Care and Community Medicine, Centre for Primary Care and Public Health, Rue du Bugnon 44, 1011 Lausanne, Switzerland; 2 Faculty of Social and Political Sciences, University of Lausanne, Lausanne, Switzerland; 3 Department of Ambulatory Care and Community Medicine, Centre for Primary Care and Public Health, Rue du Bugnon 44, 1011 Lausanne, Switzerland; 4 Neuchâtel Psychiatry Centre, Rue de la Maladière 45, 2000 Neuchâtel, Switzerland

**Keywords:** Ambulatory care facilities, Consultation-liaison psychiatry, General practitioners, Qualitative research, Mental health, Primary health care

## Abstract

**Background:**

Mental disorders are frequent in primary care settings, which is challenging for primary care physicians. In Neuchâtel (Switzerland), a Consultation-Liaison psychiatrist integrated three primary care group practices, proposing both clinical interventions and supervisions/psychiatric training. Primary care physicians’ experience regarding this collaboration was investigated.

**Methods:**

A qualitative study was conducted. Three focus groups were organized in each primary care group practice involved in the project (10 primary care physicians participated in focus groups). Data were analysed with thematic content analysis.

**Results:**

Six major themes emerged from our analysis, describing primary care physicians’ collaboration with psychiatrists: 1) Impact on a difficult to reach and “reluctant to consult” population; 2) Fluidity of the intraprofessional collaboration; 3) Influence on the doctor-patient relationship; 4) Positive emotional experiences; 5) Psychiatric counselling and training; 6) Long-term prospects for the project.

**Conclusions:**

Consultation-Liaison psychiatrist’s presence came as a relief for participating primary care physicians, facilitating accessibility to mental healthcare, introducing a common culture of care, and offering “in-situ” psychiatric training. Primary care physicians felt that their relationships with patients benefited from such interventions, being better able to deal with complex emotional experiences and found patients more confident regarding proposed care. Models of psychiatric intervention provided in primary care must establish settings of collaboration that reinforce relationships between primary care physicians, psychiatrists, and patients.

**Supplementary Information:**

The online version contains supplementary material available at 10.1186/s12875-022-01937-y.

## Introduction

Primary care physicians (PCPs) are often the first contact with a caregiver for patients presenting mental health issues [1, 2]. The prevalence of mental disorders in primary care is evaluated at 25 - 60% and it can often be the primary diagnosis for these patients [3–5]. More than half of them are not receiving specialized care (45 - 80% have no contact with a psychiatrist), being therefore treated solely by PCPs [6–9]. PCPs are thus the main actors in the prevention and treatment of mental disorders [7, 9, 10].

Furthermore, patients presenting mental disorders are often considered by PCPs as “difficult to manage” and tend to use the resources available to them to a greater extent (emergency services, social assistance, home help, multiple medical follow-ups, etc.) [11]. Often, PCPs feel lacking competency in dealing with these patients, seeking suitable training to be able to manage them [12]. A close collaboration with a psychiatrist can help PCPs to reinforce their skills and to gain confidence in dealing with mental disorders [10, 13, 14]. But such a collaboration proves to be complex, particularly due to different treatment paradigms [1, 15–17], with both disciplines constantly seeking more harmonious and effective ways of collaboration [18, 19].

To introduce a model of care that offers optimal management of mental disorders in primary care, an intraprofessional collaboration project between PCPs and psychiatrists has been developed in a region of French-speaking Switzerland (canton of Neuchâtel). Intraprofessional collaborations are collaborations between two or more disciplines within the same profession, in our case, physicians. More precisely, a liaison psychiatric consultation within primary care practices has been implemented in the canton of Neuchâtel since 2019, to promote the quality of mental health care (Table 1) [ 10, 19–21].

**Table 1:** Objectives of the “Neuchatel project”

Overall objectives	
Improvement of accessibility of psychological care for certain patients managed in the primary care context	
Screening and early treatment of mental illnesses	
Better use of psychotropic medication	
More efficient use of specialist psychiatric services	
Potential reduction of health costs	

Research into the establishment of psychiatric interventions in primary care must be extended, into different treatment contexts [22, 23]. Moreover, the experience of PCPs during such interventions is rarely investigated, whereas the emotional experiences of PCPs seem to present one of the central obstacles to the optimal management of mental disorders in primary care [12, 14]. We have therefore investigated the experience of PCPs established in group practices during their collaboration with psychiatrists within the proposed intervention, to obtain a better understanding of the factors that influence this type of intervention and to better qualify its results.

More precisely, our research question is: “What is the experience of PCPs regarding the integration of a Consultation-Liaison psychiatrist into their group practices in Neuchâtel, Switzerland, and what factors influence such intraprofessional collaboration?”

## Methods

### Setting of the study

The intraprofessional Neuchatel project involving collaboration between PCPs and psychiatrists was implemented in the canton of Neuchâtel, a French-speaking region of Switzerland, with approximately 180,000 people, and was distributed between three urban centres as well as a large area of rural territory, divided into several mid-mountain valleys. The Centre Neuchâtelois de Psychiatrie (CNP) is the canton’s public psychiatric institution. Primary care treatment is organized into private practice units, many of which are group practices. The project consisted of the physical presence of a CNP psychiatrist at such group practices [20]. Three practices have participated, each of them representing one of three different regions of the canton, consisting, respectively, of 7, 6, and 3 PCPs.

Participating psychiatrists were part of the CNP in which they followed their post-graduate training in psychiatry and psychotherapy. They were supervised by the head of the project, who is a liaison psychiatrist. One day a week, a psychiatrist was present at each group practice. PCP, patient, and psychiatrist agreed upon the need for an intervention. The psychiatrist’s activity consisted, on the one hand, of the evaluation and clinical monitoring of patients, and on the other hand, of supervision and training of the PCPs. A collaboration agreement determined the contractual terms between the CNP and PCPs.

### Design of the study and characteristics of participants

To answer the research question, a qualitative approach was employed, with a phenomenological posture, in the sense that we investigated experiences from the perspective of the individual [24, 25]. Our qualitative inquiry was based on 3 focus groups (FGs) between June and October 2019, involving PCPs from all 3 group practices [26, 27]. Purposeful sampling was used to identify and select participants who have directly experienced the phenomenon of interest and could provide rich information regarding the research question [28]. Specifically, each of the three FGs was conducted in a different region of the canton of Neuchâtel [20], with sampling covering different parts of the state (coastal, valley and mountains) to capture maximum variation in location. At the same time, participants in each FG were direct PCPs colleagues, with homogeneity of participants sought within every FG, to provide in depth description of each subgroup, to simplify analysis, and to facilitate group interviewing. There were a total of 10 participants (of the 16 PCPs working in all 3 group practices - 62.5%), 4 of whom were women in the 3 FGs; FGs had either 3 or 4 participants per group. They were aged between 33 and 55, with an average age of 43.7 and with a clinical experience ranging from 8 to 26 years (Table 2).

**Table 2:** Demographic details of the participants in the focus groups

	**Participants N (%)**
Men	6 (60%)
Women	4 (40%)
	**M (range)**
Age of the participants (in years)	43.7 (33 to 55)
Years of experience (in years)	16.6 (8 to 26)

The first and third authors (KT and PNO, a psychiatrist and a sociologist) conducted the FGs. They were not involved in the clinical project and had no hierarchical link with the PCPs or the CNP [29, 30]. The FGs took place in French. They were recorded and fully transcribed. All participants were informed of the procedures involved in the study and gave their consent. All procedures were approved by the Human Research Ethics Committee of Canton de Vaud (CER-VD), which concluded that the study was not subject to the law on medical research involving human subjects.

To design the interview guide, we drew upon two previous qualitative research studies [29]: a) a study on the referral process, as experienced by PCPs (FGs) and b) a study on psychiatrist/PCP discussion groups, with a sociological perspective (observation in situ). (See Table 3 and Additional file 1: Appendix) [31, 32].

**Table 3:** Interview guide

Interview topics: 1. how PCPs involved in the project perceived this experience, and their view on its value and limitations; 2. the reasons why PCPs call upon the psychiatrist, as they see it; 3. the substantive conduct of the collaboration between PCP and psychiatrist in the context of the project.	

The purpose of the FGs was to understand the way PCPs perceive the “psychological” or “psychiatric” issues present in their practice and how they feel about requests for a psychiatric consultation. PCPs were asked to describe specific, substantive situations, in which they identified, or suspected, the presence of psychological distress in their patients and for which they mobilized a psychiatric intervention.

### Type of data analysis

Original data were in French and themes were based on the review of these raw data. Particular attention was given to the quotes in order to maintain the meaning of the statements, while using the usual English expressions suggested by a native English speaker who translated all of the transcripts. The first and last authors (KT and SS) have verified that the translated quotes accurately convey the meaning expressed by the participants in their native language (French).

Transcripts were analyzed using thematic deductive and inductive analysis [33, 34]. At first, two members of the research team (KT and PNO) independently coded the same “raw data”. Then, they subsequently confronted the emerging codes that were compared to “extent of possible overlap”, different “sets” of codes having been combined or reorganized, with KT and PNO performing an inter-rater agreement. Once the new codes were established, they were discussed with another member of the research team (SS). At the end of this process, new codes appeared as slight variants of existing codes; and at this point, we considered the information power to be sufficient [29, 35, 36]. Afterwards, KT and PNO explored together overarching themes that emerged in all three focus groups, that were then presented to the other members of the research team, which consisted of experienced researchers (RMV, SS), to confirm that interpretations were going in the right direction. SS assumed a dual role as researcher-psychiatrist and project coordinator, while RMV conducted peer examination. We carried out exchanges back and forth until we were able to confirm that our interpretations were going in the right direction. The research team employed triangulation, using multiple data sources and methods, to increase the rigour of our study.

## Results

Our analysis of the experience of PCPs regarding the integration of a Consultation-Liaison psychiatrist into their group practices revealed two major themes, namely, *“Access to mental healthcare”* and the *“Impact of the psychiatrist’s presence in the group practice”.*

### Access to mental healthcare

Participants clearly reported improved accessibility to psychiatrists, for consultations, and for their patients, particularly for those with multiple vulnerabilities. A promotion of mental health and of fluid collaboration was thus achieved. Subthemes i) “Impact on a difficult to access and “reluctant to consult” population”, and ii) “Fluidity of the intraprofessional collaboration”, were grouped under this category.

#### Impact on a difficult to access and “reluctant to consult” population

The value of facilitated access to psychiatric treatment was emphasized by the participants, particularly for the patients who most needed it. According to them, this facilitated access was possible both with geographical proximity and with the possibility of obtaining a psychiatric consultation as soon as possible:

*P1.“The … the proximity, I think that is really positive...”*

*… … … …*

*P2.“And the delay…”.*

*P1.“External psychiatrists were saying: “Send us a letter and we’ll reply.” But then again, it can take two to 3 months”.*

They also mentioned the specific case of patients resistant to the idea of consulting a psychiatrist, although they presented multiple vulnerability factors. The presence of a psychiatrist at the practice provided the option for these patients of having initial contact with a psychiatrist. This was felt by the participants to promote mental health, in the widest sense:

*“As soon as we are talking about a psychiatrist… People think “no”. Then, afterwards, we can say: “Ah, but there’s a psychiatrist working here at the practice, he could see you here.” Then they say: “Oh yes, OK”… That’s crazy.”*

The availability of psychiatric consultation in the medical centre might also have particular relevance to the “*stigma”* associated with mental health problems and with recourse to psychiatry. More specifically, PCPs had the impression that for their patients “*it is less... stigmatizing to come to a medical practice than to go to a psychiatric hospital”.*

#### Fluidity of the intraprofessional collaboration

With the proposed project, PCPs felt that they benefited from the direct collaboration and communication with the psychiatrist. They noted that the quality of exchanges contrasted with the distant contacts they often have with specialists with whom their patients consult:

*“[…] because … Well, there are some specialists... with whom there is no communication. We don’t know what treatments they have changed... What they have found...”*

While communication between PCP and the psychiatrist often takes place by letters, emails, or phone calls, the presence of the psychiatrist at the practices concerned encouraged spontaneous forms of exchange, “*in the corridors”* or “*around the table at lunchtime”*, which the participants claimed to appreciate particularly. In addition to the exchange of important information regarding patient care, this proximity seemed to be beneficial to the PCPs interviewed, on several subjects, both clinical and organizational, having cited, inter alia the possibility of arranging joint consultations without difficulty. Overall, the psychiatrist’s ability to adapt to their respective work environments was greatly appreciated, PCPs having expressed a pleasant feeling that they are *“part of the same team”*.

### Impact of the psychiatrist’s presence in the group practice

Participants emphasized that the presence of the psychiatrist offered by the “Group Practice” project had a multidimensional impact in their daily practice. They identified for themselves the presence of various *“positive emotional experiences”* and a benefit regarding “*psychiatric counselling and training”* offered by psychiatrists, and for their patients an optimal *“influence on the doctor-patient relationship”*, having expressed overall a desire of *“long-term prospects for the project”*.

#### Influence on the doctor-patient relationship

In parallel, the participants thought that the availability of the psychiatrist contributed to a better relationship between the PCPs and their patients:

*“But it’s true that when they [the patients] feel distressed, they have a problem, they want to see us, then they would like to see a psychiatrist […]. Telling them: “OK, there will be something in two or three months…” They… don’t feel like they are being taken seriously.”*

In the same way, PCPs said that they were pleased to be able to recommend to their patients a psychiatrist in whom they had confidence. As they saw it, the prior relationship and esteem between PCP and the specialist could further reassure patients and encourage the establishment of the alliance with the specialist, thereby providing continuity in treatment:

*“It is also an extension of their [patients] confidence in us. If they have confidence in you, because you have confidence in the doctor [specialist], they are also more ready to trust that doctor.”*

Recourse to the psychiatrist also consisted of a “Concilium” for “*situations that are a bit conflictual”* or simply situations that have been “*difficult”* for PCPs.

#### Positive emotional experiences

The PCPs expressed a very positive view of the development of the project. They reported a relevant, and enriching experience, both for themselves and their patients:

“*[…] we remain very, very, very positive, and pretty enthusiastic… about this project. Because … […] This idea of bringing in a psychiatrist… A specialist at the practice, this is really, really beneficial … for our relationships, with the patients, in our follow-up… In the long term. For us, and for them.”*

They described that the simple fact of knowing that they will be able to call upon the psychiatrist without difficulty if they have any problem was for them a *“great relief”.* The possibility of sharing medical responsibility when confronted with “*complex situations”* was reassuring. Within this collaboration they felt “*less alone”*, having described the psychiatrist as *“someone with whom one can share things, and then... With whom one can feel supported”* – a fact that *“is beneficial”.* In clinical situations where PCPs may feel overwhelmed or powerless, they saw this opportunity as a “*way out”*, having been reassured by the presence of the psychiatrist:

*“When do we call the psychiatrist… when we feel powerless, I think… Most of the time, it is… patients that we see for the first time more or less urgently, then we start follow-up, and we discover that… maybe the situation is, after all, a little bit more complex and slightly beyond our capabilities […].”*

#### Psychiatric counseling and training

The PCPs called upon the psychiatrist, when they were present at their practice, for advice, particularly when they were dealing with “complex” psychiatric pathologies. More specifically, the psychiatrist’s skills were mobilized *“really for further information concerning the psychiatric diagnosis”,* concerning drug treatments, or even for doubts regarding their relational position:

*“When there are situations in which things become protracted… when things are not improving, or even deteriorating, then one has doubts: “Is this the right medication? Is the dosage correct? Or is the follow-up I provide insufficiently supportive?” And then, one has doubts, and we decide to refer to the psychiatrist…”.*

Expert assessment input from the psychiatrist was sometimes sought, particularly for “*chronic issues with health insurance questions for questions relating to extended absences from work, depression, burnouts. For questions of disability insurance documentation, etc.”* PCPs also noted that the psychiatrist *“had a better understanding of the healthcare networks”*, which extended their scope for collaboration with multiple partners, as well as the type of interventions they could offer to their patients.

The participants felt that their formal and informal exchanges with the psychiatrist involved in the project had an educational dimension, enabling them to consolidate their knowledge and skills in psychiatry:

*“I think it [the presence of the psychiatrist] enables us to have many exchanges in a general way, which… which probably modify our [clinical] view… And then again, these form like a kind of short clinical training courses ...”*

They emphasized unanimously that they would like to see further development of clinical training “in situ”, focused on *“difficult”* situations and relationship aspects. PCPs would like to implement “*Balint groups*” (purposeful, regular meeting among doctors to discuss difficult clinical situations, with a trained facilitator or leader) or similar forms of exchanges in their practices [ 37–39]:

*“Ah, for me… It’s clear that if, from time to time, we could have a Balint group with a psychiatrist who knows us, in our workplace… it would be great.”*

### Long-term prospects for the project

A major question raised by the PCPs concerned the sustainability of the project. They all agreed to underline the value of the project, wishing that the forms of collaboration it makes possible could be maintained:

*“The less positive point [concerning the project], is that we are not sure that we will be able to extend it!” [participants’ laughter].*

They also mentioned certain difficulties relating to the yet relatively unstructured nature of their collaboration with the psychiatrist. They thought that it would be helpful to make “*joint consultations”* more systematic and to establish time slots devoted to exchanges with the psychiatrist. Participants would also appreciate more frequent attendance and more psychiatric presence in each medical practice.

Respondents emphasized that they were careful to avoid offloading onto the psychiatrist what they could do themselves in patients’ care and to avoid overburdening the psychiatrist:

*“For me, the only time I was hesitant was more to avoid overloading them. Because I feel like this is such a precious resource for us, and I just wanted to keep it in reserve, er… kind of use it as a joker card”.*

It can be seen that the limits attributed to the project by the PCPs, in the true sense, related less to shortcomings or problems, but rather the wish to make it a lasting project, and overtime to be extended, developed, or expanded – i.e. that it should be allocated more resources.

In parallel, the participants reported “very positive” feedback from the vast majority of patients concerned, who – according to them – shared their sense of satisfaction and gratitude following their contacts with the psychiatrist involved in the project:

*“So, for me, people were very optimistic, and really happy. They found that it had really helped them...”*

They state that the possibility of raising with the psychiatrist the difficulties they may encounter with certain patients enabled them to manage the patients concerned more effectively, notably by enabling them to better understand certain behaviors:

*“The situations that affect us most are not necessarily the cancer we have to announce. They are often the “psych’” situations which… which stick with us, and then the patient who is not getting better and that… that we take home.”*

They also affirmed that they called upon the psychiatrist “*in emergencies”* for “*suicide situations”, the “availability”,* and the proposed intervention having an optimal outcome, having avoided “*hospitalizations”.* Finally, they felt that they respond better “*to patient needs*” concerning a psychiatric intervention.

## Discussion

The development of collaborative care models allows for seamless collaboration between PCPs and psychiatrists [19, 22, 40]. A variety of projects have been implemented in different healthcare settings [20, 40, 41]. This present project of integrating psychiatrists into PCPs’ group practices could be an innovative proposition in this direction. Our qualitative study of the experience of PCPs participating in this project has highlighted various factors that influence the collaboration between PCPs and psychiatrists.

First, we note that PCPs benefit from “*psychiatric counselling and training”*, through the *“fluidity of the intraprofessional collaboration”* that the physical presence of psychiatrists in the practices permits. This context promotes a progressive establishment of a trusting relationship. PCPs also refer to many *“positive emotional experiences”* that correspond well to their needs. Regarding the *“long-term prospects for the project”*, the concerns expressed were about the possibility of sustaining it. The PCPs also felt that the dynamic established with the psychiatrist has a (favourable) “*influence on the doctor-patient relationship”*. Overall, they noted better *“access to mental healthcare”* for their patients, through the removal of the barriers they had encountered before the presence of their psychiatrist colleague at the practice [12].

The current study addressed the need for qualitative studies on psychiatric consultations in primary care [22, 23, 42]. It was conducted in different regions (coastal, valley and mountains) of the canton of Neuchâtel, Switzerland, and contributes to the effort to gain an in-depth understanding of PCPs’ subjective experience of a psychiatric intervention in their primary care practice and to better understand the factors that influence such intraprofessional collaborations. We note that interventions at the primary-secondary care interface have been studied, with a variety of team compositions, but less specifically with psychiatric interventions in primary care [14, 43]. A systematic review and meta-analysis on psychiatric consultation in primary care included only randomized controlled trials and did not consider qualitative data, as the subjective experience of the PCP, [44] thus missing an in-depth understanding of the phenomenon. Finally, a qualitative study of behavioural health-primary care integration in New York, did not consider the PCP as the central actor in these integrations and the study’s setting (urban, federally qualified health centers) was not sufficiently representative of primary care settings [45]. Hence, through our study, PCPs who have directly experienced psychiatric interventions in their primary care practice provided rich information about these interventions, adding new insights to the existing literature. Furthermore, the proposed intervention model may be of interest to various stakeholders because it involves the presence of a psychiatrist from a public psychiatric institution, who works directly “in situ” with PCPs, a setting rarely studied before.

Regarding the results of our study, on the one hand, the various factors that influence such an intervention to emerge, are already described in similar literature, such as teamwork, optimal communication, co-location, sharing of responsibility, creation of a common culture, and coordination of care [14, 43, 45, 46]. Thus, themes related to the benefits of psychiatric training for PCPs, the accessibility of treatment for complex patients, and the desire of PCPs to make this type of consultation-liaison psychiatry more permanent [21], are the key points emerging from our study, and which have not previously been described. Also, the need for psychiatric group and/or individual supervision is clearly noted, focusing on the doctor-patient relationship. Finally, we can state that the usual barriers to treatment of mental disorders in primary care settings, such as the lack of psychiatric knowledge, the lack of accessibility and communication, and complex emotional experiences, are directly addressed by the implemented intervention, resulting in an improvement in the quality of provided care for mental disorders in such settings [12, 14, 42, 44].

## Limitations

Current findings should be interpreted considering the study’s limitations. Firstly, an inclusion bias should be mentioned, as the PCPs interviewed were part of a specific project (homogenous population). However, there was heterogeneity in terms of their sex, age, and years of clinical experience. The limited number of participants in a given territory (canton of Neuchâtel) is a second limitation. To consider this aspect, we paid close attention to access the largest number of PCPs participating in this project (62.5% - 10/16) as well as deploying it in the three different regions of the canton (coastal zone, valley, and mountains). A third limitation is that we studied only the point of view of PCPs. Interviews with psychiatrists, medical assistants, or patients would likely reveal additional themes. Future studies could focus more specifically on the experience of psychiatrists, patients, or other primary healthcare providers. Finally, due to the study design and the methodology chosen to answer the research question, our study has rich qualitative data but lacks quantitative data, which should be developed in future studies on this subject.

## Implications

Some important clinical implications emerge from our study’s results. Firstly, the need for clinical psychiatric training for PCPs is clearly emphasized, as careful consideration of this topic is a central issue in promoting mental health and avoiding stigma in primary care [2, 3, 16, 42, 46]. Given the complexity of the clinical situations encountered, but also the possibility to treat “the whole patient” in such settings, it seems important that the PCPs benefit from “holistic” training, not just skills or knowledge acquisition [16, 42]. Training that promotes awareness of PCPs subjective experience related to their inner (e.g. own feelings, attitudes or experiences) and outer world (e.g. contextual constraints or society’s dominant discourse), as well as an appreciation of the relational aspects of communication and recognition of the patient’s psychological state and associated vulnerabilities, could be achieved through psychiatric supervision (group or individual). This has already been proposed in various medical settings (oncological, palliative care) [47, 48]. Keeping in line with the culture of general practitioners and focusing on the importance of the doctor-patient relationship, we could follow the results of our study and propose the adjuvant instauration of Balint groups in psychiatric interventions in primary care settings, which could allow PCPs to become aware of how they establish relationships with their patients and team members and work through them, by stimulating their reflexivity [45–47, 49].

Furthermore, we note that spontaneous forms of exchange (in the corridors, around the table, etc.) were stimulated by the intervention context (location of the psychiatrist, accessibility, availability) and were highly appreciated by PCPs. We hypothesize that the consultation-liaison “position” adopted by the psychiatrist, offering, in addition to direct interventions, informal clinical exchanges regarding patients he or she had not yet seen, was a key element of the intervention and stimulated the PCPs’ desire to pursue such collaboration, thus providing them personal relief and rapid clinical proposals [4, 21, 22]. Such clinical exchanges, also described between PCPs and specialists other than psychiatrists, are also called curbside consultations. They play an important role in improving communication and coordination of care, but there are risks if practiced without discretion and consideration of the doctor-patient relationship [50, 51]. When performing consultation-liaison psychiatry interventions, the psychiatrist considers the various factors influencing the relationship dynamics between the PCP and his or her patient, between himself or herself and the PCP (emotions, vulnerability, and resources, contextual factors, representations, etc.) but also the interactions between these relationships, in order to propose a framework that is well-suited of such high-quality informal exchanges.

**Fig. 1:**
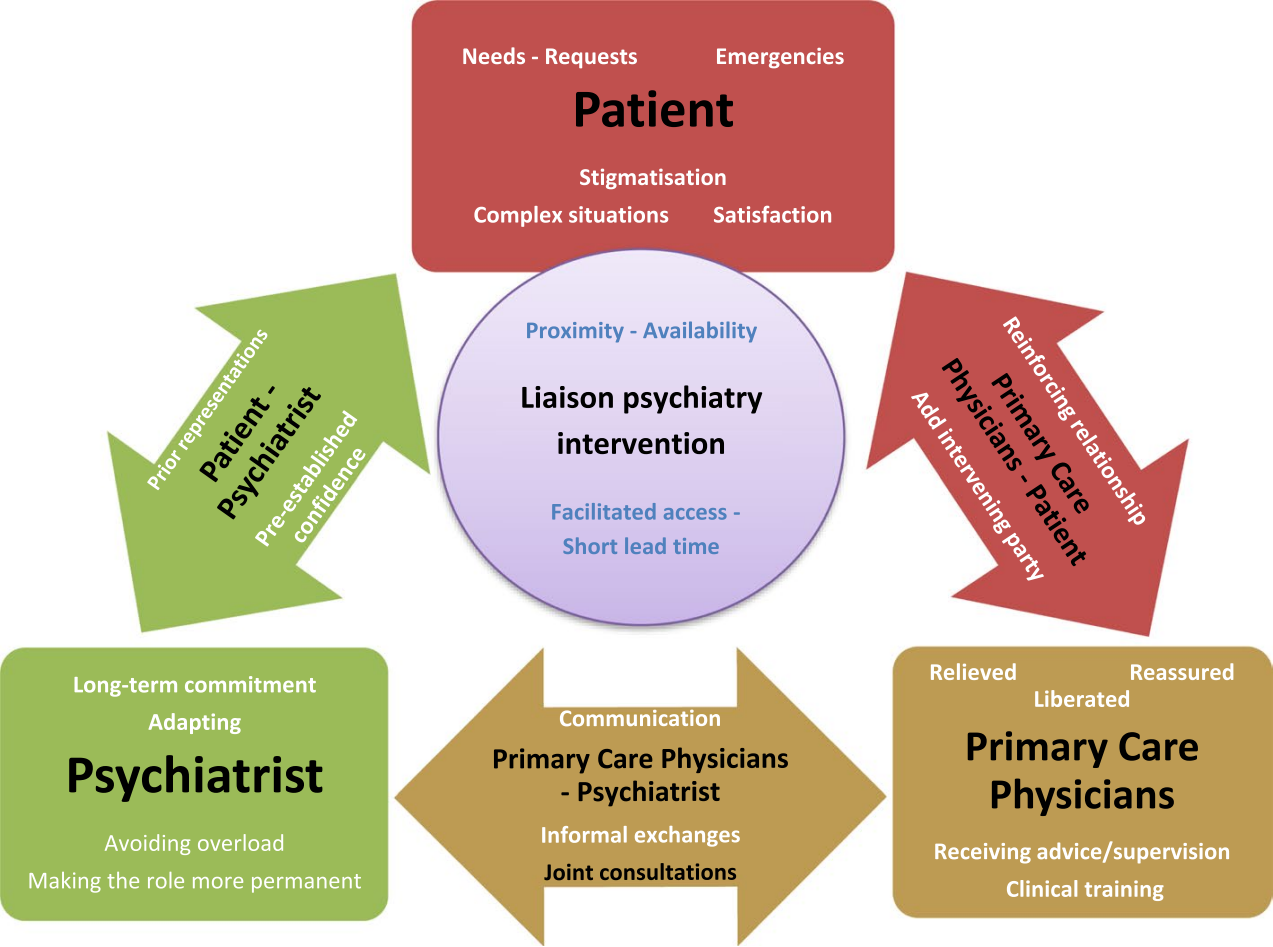
Dynamics of Primary Care Physician (PCP), Psychiatrist, and Patient relationships, regarding the proposed consultation-liaison psychiatry interventions in primary care settings. Each box represents an actor/stakeholder in the dynamics of the relationship (i.e., PCP, psychiatrist and patient). Text within each box are some emerging key issues regarding mental health in primary care. Each arrow represents a relationship between the different stakeholders (i.e., PCP, psychiatrist, and patient). Text within each arrow are some key issues affecting these relationships. The circle reports some of the key aspects of the proposed Consultation-Liaison psychiatry intervention

Finally, we can consider that identical models of intraprofessional collaboration need to be implemented in ambulatory care facilities, influencing primary mental health care on three different levels: i) for the PCPs themselves, ii) for the intervening psychiatrists, and iii) for the patients. The quality of the relationships established between these three actors [31, 37] is most likely a determining factor for the evolution of patient care and, indirectly, for the efficiency of the proposed treatment. We, therefore, postulate that in future models of psychiatric intervention provided in primary care, it is important to establish settings of collaboration that sufficiently “underpin” the relationships between PCPs, psychiatrists, and patients. A dynamic representation of such a mechanism has been conceived (Fig. 1), which may be of interest to mental health care stakeholders (PCPs and psychiatrists), educators, or primary care system planners.

## Conclusions

A global satisfaction of PCPs participating in the primary care liaison psychiatry intervention is highlighted through our study. In such an intervention, PCPs can be reassured by the presence of the psychiatrist and by his advice, especially when confronted with “complex” clinical situations. A need for a fluid collaboration with psychiatrists and clinical psychiatry training adjusted to PCPs’ specific situation is underlined. Moreover, the presence of the psychiatrist in primary care settings can give rise to an interest of PCPs for more shared time, to further extend their knowledge and clinical skills in psychiatry. Finally, such collaboration can have a positive influence on the doctor-patient relationship, through facilitated accessibility of psychiatric care, and improved quality of proposed primary care. An urgent need for models of psychiatric primary care interventions emerges that reinforce relationships between primary care physicians, psychiatrists, and patients.

### Data confidentiality

I confirm all patient/personal identifiers have been removed or disguised so the patient/person(s) described are not identifiable and cannot be identified through the details of the story.

## Supplementary Information

**Additional file 1.** Appendix. Interview guide

**Additional file 2.**

## Data Availability

All data generated or analyzed during this study are included in this published article and its supplementary information files (please refer to supplementary material).

## Abbreviations

PCPs: Primary Care Physicians
C-L: Consultation-Liaison
CER-VD: Cantonal Committee on Ethics for Research on Human Beings
FGs: Focus Groups
FLHR: Federal Law on Human Research
CNP: Centre Neuchâtelois de Psychiatrie.
