# Supplementary material for: Psychiatric consultation in general practitioners’ daily practice: a qualitative study on the experience of consultation-liaison psychiatry interventions in primary care settings in French-speaking Switzerland
Source: BMC Prim Care. 2022 Dec 7;23:316. doi: 10.1186/s12875-022-01937-y (PMC9730556; doi:10.1186/s12875-022-01937-y)
Supplement: Supplementary file 1 — Additional file 1. Appendix. Interview guide [file 12875_2022_1937_MOESM1_ESM.docx]

**Appendix: Interview guide**

1. **The reasons why PCPs call upon the psychiatrist, as they see it:**

- In which situations do you use the proposed healthcare offer? Can you give specific examples?
- Have you ever hesitated to use the proposed healthcare offer? For what reason(s)? Can you recall some specific examples?
- How do patients react when you refer to the possibility of a psychiatric consultation? Do you feel that they need to be convinced?
- In your view, does calling upon a psychiatrist constitute an issue from the perspective of your relationship with the patient?
- In your view, what are the benefits for the patients concerned?

1. **The substantive conduct of the collaboration between PCP and psychiatrist in the context of the project:**

- In concrete terms, how have you been in contact with the psychiatrist (telephone, email, face-to-face, etc.)?
- What feedback do they give concerning the consultations? How is this feedback provided (telephone, email, face-to-face, etc.)?
- Are you able to address questioning, hesitations, concerns, etc., as far as your patients are concerned?
- Do you feel that this collaboration changes your clinical perspective? If so, how does this manifest itself in your interventions?
- Does this collaboration change your experience of complex consultations?

1. **How PCPs involved in the project perceived this experience, and their view on its value and limitations:**

- In relation to the provision already in existence, in your view, what are the benefits of the project? And the less positive or less relevant points?
- Concerning further action following the project, in your view, what are the possibilities for improvement or development?
- Would you need an additional provision for general practitioners concerning “psychological” aspects of your practice (for example, training, a specific type of intervention, another mode of collaboration, etc.)? If so, which type(s)?
