# Supplementary material for: Psychiatric consultation in general practitioners’ daily practice: a qualitative study on the experience of consultation-liaison psychiatry interventions in primary care settings in French-speaking Switzerland
Source: BMC Prim Care. 2022 Dec 7;23:316. doi: 10.1186/s12875-022-01937-y (PMC9730556; doi:10.1186/s12875-022-01937-y)
Supplement: Supplementary file 2 — Additional file 2. [file 12875_2022_1937_MOESM2_ESM.pdf]

Focus Groupe 1:

MH 1: Face aux patients, c'est quand que vous faites appel au psychiatre ? Face à quelles situations? #00:00:00-0#

MF1: En fait, nous c'est, moi ça fait longtemps que j'avais envie depuis longtemps de faire appel à des psychiatres, on en avait discuté avec des patients, mais où et l'un et l'autre ont avait renoncé… Parce que c'était trop compliqué à mettre en place et puis… trop difficile et pis euh… que ça allait, on sentait que ça allait pas convenir en fait, donc, finalement moi c'est assez tôt chez mes patients que je suivais finalement, pas comme psychiatre, mais pour leurs pathologies psychiatriques alors qu'ils avaient aucun autres suivis. Donc assez tôt quand moi j'ai su que y avait ce projet, j'ai assez rapidement parlé au patients, et puis ils étaient toujours très contents à l'idée que ils puissent aussi bénéficier de de l'expertise de quelqu'un de plus spécialisé là dedans même si ils ont été, ils me disaient toujours "ah mais vous savez, j'aime beaucoup, ont continu à vous voir" mais bien sûr on va continuer à se voir, vous allez pouvoir continuer à me raconter comment vous vous sentez, y a pas de problèmes. Mais euh… on était tous très soulagés de trouver une solution comme ça confortable pour euh… pour aussi un petit peut rajouter un intervenant différent dans des situations qu'étaient compliquées depuis longtemps. Donc c'est vrai que moi tous les patients que j'ai référé, euh… ça s'est fait assez vite, et c'était souvent des patients que je suivais depuis longtemps euh… pour euh… des symptomatologies quasiment uniquement psychiatriques et pis euh pour lesquels ont avait pas tellement d'autres possibilités de… de suivi et puis c'était pas grave au point où il fallait absolument passer par un [inaudible] ou euh… ou vraiment essayer avec le CNP même si des fois c'était compliqué, on se disait "ouais bon ben voilà on essaye quand même [imitation d'une voix blasé] un peu comme ça et pis c'était… c'était vraiment profitable à personne mais finalement c'était un peu la moins mauvais solution. Donc c'est vrai que moi je me suis assez rapidement précipité, d'ailleurs, pauvre CL-Psy, elle a du, elle a du avoir beaucoup de mes patients #00:01:59-3#

MH2: Des fois, moi je sais [pas sure] quand on a des situations où ça se prolonge, ça s'améliore pas ou ça se détériore, pis on a des doutes, "est-ce que c'est le bon médicament ? Est-ce que c'est le bon dosage ? ou est-ce que c'est le suivi que j'y donne qu'est pas assez soutenant ? Et puis on, on a ses doutes là et pis se dit on va voir en référant à la psychiatre, des fois ça, ça permet de, voilà de voir si on est fait fausse route ou y a des choses qu'on avait pas intégré dans l'approche du patient. Euh…j'pen… #00:02:31-9#

[intervention de la MF2] : moi je pense… #00:02:31-9#

MH2: …et puis des fois c'est à leur demande. #00:02:33-5# #00:02:34-2#

[intervention de la MF2] : oui, #00:02:34-2#

MH2: demande du patient #00:02:36-4#

[intervention de la MF2] : oui. #00:02:35-2#

MH2: qui vient clairement et puis qui dit vous connaissez un psychiatre #00:02:38-0#

[intervention de la MF2] : oui. #00:02:38-0#

MF2: Mais… Moi je pense que y a plus… vraiment pleins de cas de figures différents. Y a justement des patients chroniques que tu suis depuis longtemps, et puis que personnellement t'aimerai adresser au psychiatre depuis longtemps parce que t'es embourbé euh… jusqu'au cou. Pis que t'as jamais pu, parce que que y a c'te barrière de l'accessibilité pis là, tout d'un coup, t'as un psychiatre au bout du couloir, tu, tu dis au patient, "mai écoutez, je crois que ce serait bien et pis là ben je peux vous proposer quelqu'un, vous aurez rendez-vous rapidement". Et puis du coup ils adhèrent, parce que c'est facile d'accès. Y a les patients qui viennent en crise, [pause courte] ou… ou vraiment euh, tu les a jamais vu pour un problème psychologique, dépressif ou comme ça, pis qui arrivent en crise et pis t'es super content de pouvoir avoir un rendez-vous dans les deux semaines chez ta collègue psychiatre, parce que tu te dis "wawouh, là, là ça craint", parce que… #00:03:25-8#

MH1: est-ce que vous avez un cas une situation pratique, ou? #00:03:01-9#

MF2: Moi j'en ai une en tête, parce que ben on a notre collègue là, qui a… Qui nous à lâché. Pis du coup on s'est retrouvé avec beaucoup… Enfin tous ses patients euh, sur les bras. #00:03:38-9#

MH1: Hum. #00:03:38-9#

MF2: Et pis, ben, j'ai un patient en tête qu'était suivi par elle. Un jeune d'une vingtaine… #00:03:42-4#

MH2: — he tu veux dire notre ancienne collègue ? #00:03:44-3#

MF2: Notre ancienne collègue, euh… un jeune d'une vingtaine d'année qui… Qui est rentré de vacances et puis qui, qui est venu me voir, parce qu'il pouvait pas retourner travailler parce qu'il était probablement en burn-out, mais… Il y avait une situation familiale, professionnelle qui.. Son père était son employeur. Pis je le sentais vraiment…euhmmm [comme une hésitation]. Un jeune d'une vingtaine d'année, qui voulait pas retourner travailler, qui se posait mille questions sur son avenir et pis ben je suis pas son médecin traitant, parce que je faisais que de remplacer en urgence son médecin qui n'est plus là. Et pis je l'ai adressé à CL-Psy, pis qu'est-ce que j'étais contente de pouvoir aussi rapidement, savoir que y avait quelqu'un pour euh, pour l'accompagner. #00:04:43-0#

MF1: C'est vrai aussi que on a énormément de patients. On a des agenda extrêmement pleins. Et quand on voit arriver quelqu'un… #00:04:51-2#

MF2: ouais. #00:04:51-2#

MF1: …en pleurs, en disant "ça va pas du tout à mon travail, euh… Mon chef c'est horrible, il m'a convoqué, il m'a dit ça, ça, ça, je pouvais même pas me défendre. [Inspriation] C'est dur

pour nous parce que on sait que ça va être compliqué d'assurer le suivi, même simplement en termes de temps. #00:05:12-2#

MF2: De logistique, ouais. #00:05:12-7#

MF1: Moi je sais que des fois, je suis là "ah non c'est pas vrai". #00:05:15-0#

MF2: Humhum. #00:05:15-0#

MF1: Bon ben faut qu'on se voit la semaine prochaine, on regarde l'agenda, on se dit déjà, "wawouh, super". Donc on les met dans une plage d'un quart d'heure, en sachant de toute manière ça va prendre une demi heure. Une demi-heure trois quart d'heure donc d'emblé on se met déjà en échec en sachant que voilà, on va prendre du retard. Moi je trouve que c'est quand même une partie stressante quoi. On se dit, "ouais bon ben, en même temps je peux pas le laisser tomber. Visiblement, il a besoin d'aide. Mais en même temps, moi cette aide, je sais pas si mes, simplement ma disponibilité en termes de temps, je, je l'ai. #00:05:38-7#

MF2: ouais. #00:05:39-2#

MF1: Et ça, moi j'trouve c'est quand même pour ça, c'est-à-dire, [inaudible] de simplement pouvoir déléguer une partie… #00:05:42-8#

MF2: oui. #00:05:42-8#

MF1: …De se dire, ben voilà, comme ça, même si, si je le vois qu'une fois par mois, ça peut suffir. #00:05:47-5#

MF2: Humhum #00:05:47-5#

MF1: Et ben franchement, moi je trouve que c'était très précieux. #00:05:49-4#

MF2: Et après, y a des autres cas de figures, où j'étais contente d'avoir CL-Psy, c'était simplement pour les histoires de, de traitements. Parce que ben, on prescrit un peu des choses qu'on connait un petit peu, pis quand on voit que, [tentative d'intervention d'un des MH]…Franchement mieux, ou au contraire, ça ça s'aggrave, euh… Ou bien qu'ils ont déjà une liste…#00:06:14-5#

[MH2 en fond]: Y a aussi ça. #00:06:14-2#

MF2:… de médicaments comme ça, pis y a mille interactions. Ben des fois, simplement, de pouvoir demander euh, "écoute dans cette situation, qu'est-ce que tu ferais ?" quel médicament tu mettrais ?… #00:06:27-8#

MH2: Ouais. #00:06:29-2#

MF2:… Ou enlèverait?" Et ça, ça j'ai eu plusieurs fois le cas de figures. #00:06:35-5#

MF1: Ah, moi pas trop. Mais c'est vrai que pour ça, j'ai entre guillemets de la chance. Mais comme j'ai le "home", je suis beaucoup confronté à ça. Et pis ben là y a l'équipe, y a la psychogériatrie #00:06:41-7#

MF2: ouais. #00:06:30-2#
MF1: qui vient souvent. Y a la pharmacien qui vient souvent donc, du coup, de ce point de vue là, c'est vrai que ça m'a donné un peu une expérience différente des médicaments psychotropes euh, chez les gens âgés, mais, tu… Tu peux utiliser cette expérience pour les patients adulte, ça va. #00:06:55-0#

MF2: Humhum. #00:06:53-4#

MF1: Donc euh, c'est vrai que de ce point de vue là, moi je me sentais moins mal à l'aise. C'était plus en termes de temps, et pis en termes de… De vraiment le côté pratique de, de la chose quoi. Ouais ça va m'a vraiment bien aidé. #00:07:04-9#
[Pause] #00:07:06-3#

MF2: En fait, euh… Elles s'aident pour pleins de choses, pour pleins d'aspects moi je trouve. #00:07:12-2#

MF1: Ouais, ouais moi je trouve ça… [les deux MF parlent en même temps] #00:07:12-0#

MF2: Moi je trouve que c'est une expérience hyper enrichissante et pis pratique. Cette disponibilité à tous les niveaux. #00:07:21-9#

MH2: Pis elle connait mieux aussi #00:07:22-6#

MF2: Le temps… #00:07:22-9#

MH2 : mieux les réseaux, aussi #00:07:24-0#

MF2: En terme de connaissances #00:07:25-6#

MH2: De dire "ah mais là, on pourrait mettre un hôpital de jour. On pourrait faire de l'ergothérapie, on pourrai faire…". Et ça c'est des réseaux que nous on connait pas et puis, du coup, [acquiescement verbal de MF2: "ouais"] On y a même pas pensé pour nos patients [acquiescement verbal de MF2 "ouais"]. Et on se dit, c'est chouette qu'elle ait [acquiescement verbal de MF2: "ouais"] ses propositions-là parce que… On a peut-être pas assez de… De renseignements [acquiescement verbal de MF2: "hum hum"] par le CNP pour nous dire "voilà ce qui existe, ce qu'on a en place, vous pouvez faire appel à nous dans ces situations-là [acquiescement verbal de MF2: "hum hum"]. ça nous fait du bien d'avoir quelqu'un qu'a un pied là-dedans. [acquiescement verbal de MF2: "ouais"] Et puis qui… [acquiescement verbal de MF2: "hum hum"], qui peut nous ouvrir ses portes-là. #00:07:58-9#

MF2: Complètement. #00:07:59-8#

MH1: Du coup au contraire, est-ce qu'il y a des fois où vous hésitez, où vous avez des patients, vous hésitez de faire recours … #00:08:09-9#

MH2: oui. #00:08:11-2#

MH1:…à des psychiatres ? #00:08:11-8#

MH2: Ah, ah… Avec euh, avec la psychiatre ici? #00:08:14-6#

MH1: hum hum #00:08:15-3#

MH2: Ben c'est juste pour pas la surcharger #00:08:17-6# [rires de MH2 et MF2] #00:08:18-6#

MH2: Non, mais en fait c'est… Pour moi c'est un des trucs principaux. Je me dis "la pauvre elle a déjà tellement, j'hésite à lui donner parce que, est-ce que ça vaut vraiment la peine, je peux pas le régler tout seul. J'me pose la question, je me dis mais là peut-être que j'arrive à… Et pis là ben on peut lui en parler deux minutes justement à midi, en disant "voilà, j'ai ce patient-là, mais je suis pas sure que ce soit vraiment nécessaire que tu le vois"… Et puis, donc oui, des fois j'hésite, mais c'est plutôt pour euh… Une crainte de la surcharger, ou que le patient n'y aille pas. [acquiescement verbal de MF2: "hum"] J'hésite parce que je le sens pas prêt à y aller. #00:08:45-9#

MH1: Hum #00:08:46-0#

MH2: Je… Quand on discute, j'ai l'impression que c'est comme pour arrêter de fumer, on dit je suis une étape trop vite par rapport à lui. [acquiescement verbal de MF2: "hum hum"] il propose un suivi psychiatrique, mais il va pas s'y rendre. Là, peut-être que ça prend tout son sens de faire une consultation commune la fois d'après. #00:08:58-3#

MF2: Hum hum #00:08:58-9#

MH2: Moi je l'ai jamais proposé, donc je sais pas comment s'est vécu par le patients, s'ils sont réticents ou pas de nous avoir avec le, le psychiatre. #00:09:03-4#

MF1: ça peut être vécu de deux façons, soit ça peut être vécu comme "ah ben c'est chouette, je serai pas tout seul… #00:09:07-6#

MF2: Ouais. #00:09:08-2#

MF1: …Par cette personne inconnue". Soit ça peut être comme "ok, super. Il sent que je suis pas ravi, on va me forcer la main [intervention de MH2 mais inaudible] #00:09:12-6# … Tu vois. #00:09:12-9#

MH2: Ouais. #00:09:14-6#

MF2: Alors moi l'expérience que j'ai eu avec euh, ma patiente, elle était assez réticente. Mais en fait moi, c'est moi qui avait besoin qu'elle soit suivi par un psychiatre, parce que c'est moi qui était en train de décompenser, parce que j'en pouvais plus de ce suivi, qui est extrêmement lourd. Et puis euh… Ouais je lui ai un peu forcé la main, en lui disant "non non, mais ce serait vraiment bien pour vous", sous-entendu "pour moi aussi". Et pis euh.. #00:09:36-6#

MH1: Qu'est-ce qui était "lourd" dans cette situation ? #00:09:37-5#
[rire de MF2 ou MF1] #00:09:42-9#

MH2: Mais elle consume déjà hyper souvent, hors des rendez-vous, elle revient en urgence. Elle a un côté un peu euh… #00:09:58-1#

MH2: …hypocondriaque #00:10:03-5#
MF1: Elle est extrêmement hypocondriaque #00:10:08-1#
MF2: elle a un trouble anxieux généralisé et pis euh…Alors ça va mieux maintenant, elle est plus allée aux urgences depuis plus d'une année, mais y a un temps je l'a voyait toutes les semaines et elle appelait encore trois fois par semaines entre des rendez-vous. Donc moi j'en pouvais plus… Je… #00:10:30-5#

MF1: Surtout qu'elle une… Une méthode [intervention de MF2 "oui"] de prolongation extrêmement pauvre euh… #00:10:32-2#

MF2: Elle sait pas parler #00:10:32-2#

MF1: extrêmement peu informative [rires] #00:10:34-0#

MF2: Elle sait pas parler, elle sait pas transmettre les informations #00:10:34-3#

MF1: Qui consiste à donner des silences et puis ensuite à dire "mais vous voyiez" #00:10:37-3#

MF2: Vous… Ouais. #00:10:37-9#

MF1: Non ? [rires] #00:10:38-4#

MH2: Mais c'est qu'elle connait pas son corps aussi, pis du coup elle arrive à pas a exprimer [intervention de MF1 "non"] ce qui se passe, que y a quelque chose qui va pas [intervention de MF1 et MF2 "oui"] et qu'on va deviner ce que c'est [intervention de MF1 et MF2 "oui"]. Et, du coup… #00:10:45-8#

MF1: Sur la base d'aucuns symptômes #00:10:46-4#

MH2: ça te met aussi de la pression à toi de, de, de se dire mais comment je vais réussir à comprendre ce qu'elle veut me dire, [intervention de MF2 "ouais"], parce que elle elle arrive

pas à l'exprimer. [intervention de MF2 "ouais ouais"]. Euh… ouais. Pis en plus elle a des choses graves quand même dans ses antécédents donc [intervention de MF2 "oui"] t'as pas envie de louper des choses #00:10:56-3# tu peux pas juste baliser [? inaudible]…

MF2: oui y a des trucs somatiques euh… Qu'elle a vécu euh… Donc voilà mais… Y a… Y a énormément de somatisation, enfin y a pratiquement que de la somatisation chez elle mais. [inspiration] Pis du coup ben voilà, pour moi c'était important de me décharger un peu, histoire de pouvoir un peu espacer les consults chez moi parce que mon agenda… #00:11:13-4#
MF1: inaudible #00:11:15-6#

MH2: La preuve c'est qu'on la connait tous #00:11:17-5#
MF2: Ouais, c'est vrai [rires] #00:11:16-5#

MH2: donc elle a épuisé déjà son médecin traitant, mais presque elle a épuisé tout le cabinet. Parce qu'on se remplace quand on est plein [intervention de MF2 "ouais"] et puis du coup, ben nous la connait aussi, on l'a vu, donc elle a épuisé la structure hein. #00:11:27-3#
MF2: Ouais, pis même déjà CL-Psy hein, euh… [rires de MF1, MF2 et MH2]. Du coup ben voilà comme elle elle est allée la première fois, elle était assez réticente. et pis euh… Elle a, apparement, on avait commencé la consulte, j'ai juste du m'absenter une minute pour régler un truc avec l'assistante et pis CL-Psy me dit "mais tu sais", après la consulte elle me dit "mais tu sais quand tu t'es absenté, elle m'a regardé comme ça pis elle m'a dit : "mais vous aller m'hospitaliser?"". Enfin voilà, elle, elle était sure que je l'envoyait chez le psychiatre pour se faire hospitaliser, alors que c'était pas du tout dans ce sens-là, mais [inspiration]. Pis après ben, elle a compris que, non c'était juste histoire de faire un travail d'équipe, de se coordonner, de coordonner les soins et pis que c'était pas que je m'en débarrassais non plus mais je pense être effectivement vécu de pleins de façons différentes. #00:12:23-9#

MH2: En reparlant pour toi, toi ça t'as redonné un nouveau souffle ? Ca t'as redonné de l'énergie ? Des nouvelles… ? #00:12:27-5#

MF2: Pas pendant longtemps mais… [rires] #00:12:27-8#

MH2 [amusé]: Mais pendant un petit moment quand tu… D'avoir fait ça quoi. #00:12:30-5#

MF2: Oui. #00:12:30-9#

MH2: Ouais. #00:12:31-0#

MF1: Ouais quand on la voit sur notre agenda, ça illumine pas vraiment notre journée [rires de MF2 et MH2], alors qu'elle est très gentille hein [rires] mais… #00:12:38-0#

MF2: D'ailleurs [inaudible] #00:12:39-0#

MH1: mais du coup, sur la relation médecin-patient est-ce que vous avez l'impression que, la présence du psy ou votre relation il y a un enjeu ou pas finalement ? #00:12:49-7#

MF2: Bah en l'occurence avec cette… Tu dis pour la consultation de groupe ? Alors moi je pense… #00:12:53-8#

MH1: Pour votre consultation finalement… individuelle. C'est-à-dire avec.. #00:12:58-6# #00:12:59-3#

MF2: Ah s'y il y a eu euh…une incidence #00:12:59-3#

MH2: Si on a envoyé quelqu'un, si on a l'impression quand y revient enfin que y a eu une perte de temps #00:13:00-9#

MH1: Après, avant ou le contraire. Ouais qu'est-ce qu'il y a comme enjeu? #00:13:05-7#

MF2: Ah moi je trouve que ça change rien [intervention de MF1 "Ca change pas, ouais"] #00:13:06-6#

MF1: moi les gens ont beaucoup remercié, surtout. Parce que justement ils étaient très contents du suivi, de la disponibilité, de la facilité… Et puis c'est vrai que je remarquais que ben, évidemment, je les voyais plus pour des problèmes purement somatiques que pour leur problèmes psychiques. Pis ça donnait de ce point de vue là une relation légèrement différente [intervention de MH2 "ouais"]. Mais quand même ils me disaient, on a beaucoup discuté, avec la doctoresse de ç, elle m'a conseillé ça, ça, ça. Ils me donnaient quand même toujours un feedback de ça moi. [intervention de MH1 "hum hum"] #00:13:33-4#

MH2: Justement moi je pense, pour moi, peut-être de pas aller voir en consultation commune et pis le fait que, ouais je suis pas très fort en psy, contrairement à Ariane qui, voilà qui prend beaucoup de temps, qu'a beaucoup d'empathie. Peut-être que ça, ça, ça donne une mauvaise image, enfin, pas une mauvaise mais, les patients ont l'impression "ouais je vais voir un doctor… Quand j'ai un problème physique". Et puis psychique, peut-être qu'ils ont, ils auront moins envie de se, de se confier parce qu'ils ont l'impression que c'est pas mon domaine. [pause] [intervention de MF2 "ouais"] Je dois dire égoïstement que ça me dérange pas [rires de MF1], mais, mais c'est vrai que c'est peut être pas bien #00:14:12-8# euh… C'est peut-être pas une #00:14:13-8#

MF2: non moi je suis pas sur. #00:14:13-7# #00:14:16-8#

MF1: parce que les gens de toute manières #00:14:15-4#

MH2:…Bonne chose. #00:14:16-1# #00:14:16-5#

MF1: [inaudible]… Compliqué de, de… Enfin, quand tu te sens pas bien, tu veux déjà voir un médecin. Et puis, bah, tu sens que t'es pas bien au niveau psychique, mais… Tu vois, je veux dire, t'habites au Locle, tu te dis, mais est-ce que tu penses vraiment que tu vas voir un

psychiatre ? Non [Acquiescement de MH2 “non” en même temps]. Tu vas appeler ton généraliste. #00:14:31-5#

MH2: Bien sur. [acquiescement de MF2 “hum hum”] #00:14:31-0#
MF1: même si tu trouves…   #00:14:31-9#

MH2: Mais sans trop d’attentes quoi. #00:14:31-6#

MF1: …que niveau communication, tout ça. [rires de MH2] #00:14:33-5# Mais oui, mais voilà mais… Tu vois, je veux dire, pis tu vas pouvoir au moins l’aiguiller et tout ça. Moi je pense que ça va pas changer grand chose par rapport aux nombres de consultations. En tout cas moi j’en ai eu tout pleins depuis. #00:14:41-4#

MH2: Oui, non mais c’est vrai. #00:14:41-5#

MF2: Moi ça a rien changé, je trouve. #00:14:42-0# Le, les patients que j’ai envoyé chez CL-Psy ou même chez un autre psychiatre, euh… La majorité des consultations que j’ai avec ces patients ça reste autour du sujet psy. Le somatique, des fois je suis là “mais j’aimerai bien intégrer un peu de somatique dans ma consulte, ça fait longtemps que je l’ai plus examinée”. Et pis finalement, on continue tellement à parler de la sph— sphère psychologique que… En fait. #00:15:15-9#

MH2: Le remplacer pour euh.. ?   #00:15:17-1# — Non [MF2]

MF2: Non #00:15:17-1#, c’est pas que le psychiatre me remplace, c’est juste qu’il m’accompagne, ou qu’il accompagne le patient mais… #00:15:22-7#

MF1: Il te décharge #00:15:25-1#

MF2: Il me décharge un petit peu mais… [Acquiescement de MH2 “hum hum”]. Je… Ouais. La, la plupart des patients qui sont suivis par le psy, quand je les revois, ça reste encore psy ma consulte. [Acquiescement de MH2 “hum”]. Donc euh.. #00:15:35-4#

MF1: C’est vrai qu’on a quand même beaucoup, beaucoup de consultations qui sont, au moins en partie euh, basée sur des problèmes psychiques hein. C’est quand même une grande, grande, grande partie de notre consultation. Même pour Manu et moi #00:15:49-1# [rires de MF2 et MF1]. Mais toi par exemple… C’est vrai que, quand même hein. Moi je vois mon agenda ce matin, y en a quand même pas mal, enfin. Et pis même s’ils viennent pour tout autre chose, et ben euh… ils évoquent quand même… Là j’ai un patient qui venait parce que il avait une toux et il m’a parlé de son burn-out qu’il avait fait en 2003, il éprouvait le besoin d’en reparler. C’est quand même souvent hein. #00:16:07-6#

MF2: Ouais. #00:16:11-8#

MF1: C’est quand même vraiment une des — un — une des causes de … Pis pas que de consultations, mais surtout de suivi. Parce que finalement [Acquiescement de MH2 “hum”].

#00:16:23-3# ben voilà les gens ils ont une angine, ils vont pas revenir nous voir cinq fois. #00:16:25-7#

MF2: Non. C'est pour ça que j'aime bien de temps en temps être de garde parce que là ça fait un peu plus du somatique. Et moi de psy. #00:16:37-3#

MF1: … Tellement contente de pouvoir vraiment soigner quelqu'un de manière plus définitive [rires de MF1 et MF2] #00:16:41-1#

MF2: Et en quinze minutes. [rires de MF1 et MF2] #00:16:41-7#

[pause — toujours rires de MF1 et MF2] #00:16:45-2#

MH1: [inaudible]… #00:16:46-0#

MF2: Hein ? #00:16:47-2#

MH1: C'est compliqué les [inaudible] de psy ou? #00:16:48-9#

MF2: mais moi j'ai jamais fait de psychiatrie pendant ma formation. J'ai fais ce CAS de… psychosociale mais voilà — je suis pas allée au bout. #00:16:53-0#

MF1: Moi j'ai fais que de la psychogériatrie [acquiescement de MF2 "oui"] #00:16:57-4# c'est pas la même chose hein. #00:16:58-2#

MF2: Et pis moi je me sens, ben j'ai fais le CAS de psychosomatique pour avoir quelques outils parce que je me sentais tellement … [pause] à côté de la plaque #00:17:03-9# Intervention de MF1: moi ce que je pensais… #00:17:05-8# … Pas comme médecine générale, j'aurai autant de problèmes psychiatriques. Franchement, je pensais pas. #00:17:11-7#

MH2: Euh c'est vrai que pour… #00:17:11-4#

MF1: Ben voilà c'est comme ça, c'est comme ça. On va pas non plus le changer. #00:17:13-6# [acquiescement de MF2 "non"] #00:17:13-4#

MH2: Ouais. Je pense pour moi les, les, les régler, suivre le patient pendant ses périodes difficiles et l'épauler, ça aide beaucoup dans la, la, la relation thérapeutique pour après [acquiescement de MH1 "hum"] #00:17:21-5#. Pour les étapes d'après, parce que, il se souvient qu'on était là à ce moment-là, qu'on, qu'on était ensemble pour l'aider à passer ce cap. Et pis, je pense pour le, l-la-la confiance, le lien thérapeutique [acquiescement de MF1 "c'est vrai" ; acquiescement de MF2 "hum hum"] #00:17:30-1#, c'est bien quoi de d'avoir ça. #00:17:32-4#

MF1: Mais quand on voit ça patine… #00:17:33-8#

MH2: Mais… #00:17:33-6#

MF1:C’est vraiment #00:17:34-0#

MH1: Voilà #00:17:34-2#

MF1: …Bien d’avoir quelqu’un #00:17:34-7#

MH1: Sur le moment, quand ça patine… #00:17:35-9#

MF1: Sur qui on peut… Ouais. #00:17:37-2#

MH2: ….Qu’on comprend pas trop si c’est pour un arrêt de travail… Si c’est pour euh… #00:17:40-0#

MF1: Ben voilà, je voulais parler de ça, les arrêts de travail. [acquiescement de MH2 “aussi”] #00:17:41-3# Moi j’étais aussi souvent contente [acquiescement de MF2 “oui”] d’avoir un autre avis [acquiescement de MF2 “oui”] #00:17:42-7# [acquiescement de MH2 “ouais”], Qu’CL-Psy me dise “non mais tu as raison, pour le moment, elle ne peut pas travailler”. Parce que c’est vrai que quand ça se prolonge [acquiescement de MF2 “hum hum”] #00:17:48-6# au-delà plusieurs mois et tout ça #00:17:50-4#

MF2: Qu’il faut justifier #00:17:51-1#

MF1: On est mal à l’aise… #00:17:52-0#

MH2: Qu’il faut justifier, t’as pas de diagnostique psychiatrique que… #00:17:52-0#

MF1: …On rempli les trucs pour la perte de gain etc. #00:17:54-1#

MH2: Complètement embêtant #00:17:55-8#

Acquiescements de MH2 et MF2 “ouais”] #00:17:56-5#

MH1: Comment ? #00:17:57-2#

MH2: Ben, on doit justifier, on a des assurances après un mois d’arrêt, puis on, ils nous demandent le code SIM, [acquiescement de MH1 “hum”] et puis on n’en a pas, si on en a pas … [inaudible] #00:18:05-8#

MF1: Alors moi je, je… [inaudible] #00:18:06-9#

MH2: On met ce qu’on pense nous, #00:18:08-5# mais du coup c’est pas béton béton. #00:18:10-6#

MF2: carrément pas du tout. #00:18:13-6#

MH2: est-ce qu'on a vraiment la légitimité de l'avoir mis [acquiescement de MF1 et MF2 "hum hum"] #00:18:17-9# euh,... #00:18:19-9#

MF1: c'est vrai que moi quand j'ai du remplir certains de ces rapports et pis que c'était des patients qui étaient suivis conjointement par CL-Psy, on a la petite question est-ce le patient est suivi par un autre spécialiste #00:18:30-4#

MH2: Voilà, t'es bien content de pouvoir mettre...[inaudible] #00:18:32-1#

MF1: Ouais, on se sent, on se sent plus à l'aise de pouvoir dire "non, non mais c'est pas que moi qu'estime que voilà, vraiment..." #00:18:38-4#

MH2: "C'est pas moi qui fait durer les choses quoi" #00:18:39-6#

MF1: Ouais, hum. #00:18:39-7# [acquiescement de MH1 "ouais"]. Pour ça moi, des fois même les expertises des pertes de gains, avant que y ait CL-Psy, j'étais presque contente de voir que les psychiatres, ils me suivaient [acquiescement de MH1 "hum"]. Même une fois [rires] y en a un qui m'avait appelé en me disant "mais il faut absolument faire quelque chose, ce patient, il va se suicider" moi j'étais là "quoi ? Aaaah — " [imitation de peur puis rires] #00:18:52-4# — "Panique! " #00:18:53-3# Donc ouais c'est vrai que franchement, moi même des fois les expertises des assurances, je suis contente d'avoir un peu un autre avis, de voir que je suis un peu dans le juste quand même aussi par rapport aux arrêts de travail [acquiescement de MH1 "hum"]. Parce qu'on se sent quand même aussi un peu mal à la société — #00:19:04-2#

MF2: Mais, c'est ça, c'est que, tout, tout... Quand ça touche la sphère a-sécurologique[? -pas bien compris], AI et tout ça, moi je me sens complètement démunie. #00:19:10-8#

MH2: Pis pour le patient lui-même [acquiescement de MF2 "oui"], des fois c'est délétère de le mettre trop à l'arrêt parce que [acquiescement de MF2 "absolument"], il a be... De toute façon il doit retourner au travail un jour. Et puis, des fois, on se dit, ouais est-ce que c'est maintenant que je dois le remettre un petit peu à 50, ou c'est trop prématuré, pis un échec de plus ça va le... Le tirer encore plus en bas [acquiescement de MF2 "hum hum"] #00:19:28-9# Euh... C'est des choses qu'il faut sentir et puis qui sont pas toujours facile. #00:19:32-4#

MF1: En sachant —Ouais, voilà, en sachant que voilà, si, si tu leur met [inaudible] pis que c'est pas le moment, ben voilà,...ça t'façon... #00:19:41-7#

MH2: Ce sera un échec, mais ouais, non c'est...
#00:19:41-4#

MH1: Mais du coup pour le patient [pause]...Qu'est-ce que vous pensez, quel sont les bénéfices pour le patient d'avoir un psy dans le cabinet ? #00:19:53-7#

MF1: Mmmh, je pense multiples, je dirai déjà ben d'avoir un autre regard, peut-être aussi d'avoir une légitimité par rapport à leur maladie. Euh, aussi quand même le traitement médicamenteux, je pense que c'est quand même bien. Et pis l'aspect que disait Manu,

justement euh,… Le côté réseaux, mieux connaitre le tissu euh, des, du traitement des maladies psychiques neuchâtelois, ça je pense qu'aussi c'est vraiment bien. #00:20:29-7#

MH2: Puis qu'ils sachent qu'on communique, je crois que ça les rassure aussi, de savoir qu'on en a parlé, qu'ils sont au courant et puis que c'est pas juste loin, et comme ça. Il me semble que ça ça les rassure aussi de dire, quand on leur dit "je vous la conseille, on a vraiment des bons échos, c'est une personne bien", ils y vont déjà en confiance généralement, pis on peut le dire, parce que c'est une personne bien. Et puis que, quand ils reviennent "ah est-ce qu'elle vous a parlé de?" et puis ça on peut dire "oui, oui, elle m'a dit qu'elle avait changé de traitement et tout ça" [plusieurs acquiescements de MF2 "hum hum"] #00:20:53-3#. Eux, ils se sentent bien pris en charge, donc je pense que ça c'est aussi un point positif ici dans le cabinet. Pis bon après y a quand même la disponibilité faut dire, qu'est, qu'est, [plusieurs acquiescements de MF2 "ouais"] qu'est, qu'est, qu'est, qu'est beaucoup plus #00:21:05-1# …

MF2: Ouais parce que y a pleins de patients #00:21:05-7#

MH2: Ils ont des rendez-vous beaucoup plus rapidement, avec un suivi #00:21:06-5#

MF2:Qui… qui sont… Avant qu'on ait CL-Psy qu'étaient d'accord d'entreprendre un suivi psycho-thérapeutique #00:21:10-9#

MH2: c'est ça [inaudible] #00:21:12-0#

MF2: Pis on leur a dit "ben écoutez, non j'ai personne à vous conseiller. Prenez la liste de téléphone pis vous essayer". Pis quand après trois mois, ils ont toujours fait chou blanc [acquiescement de MH2 "c'est ça"], bah, ben ils laissent tomber. Pis du coup ils passent à côté d'une prise en charge qu'ils auraient accepté [acquiescement de MH1 "hum hum"] si elle avait été disponible donc euh… #00:21:25-7#

MF1: Faut déjà réussir à joindre quelqu'un. #00:21:26-4#

MF2: C'est ça. #00:21:27-3#

MF1: Genre Gern [?], faut appeler le… #00:21:28-1#

MF2: le mercredi entre huit heures et quart et neuf heures #00:21:30-7#

MH2: c'est impossible. #00:21:30-9# [acquiescements de MF1 "ouais"] #00:21:31-6#

MF2: ouais ou dans le genre [rire suscint] #00:21:32-8#

[pause] #00:21:32-9#

MF1: Faut se mettre un rappel, parce que sinon tu le rates [rires] [acquiescements de MF2 "hum hum"] #00:21:36-7# ouais c'est pas évident #00:21:36-7# [intervention de MF2

“inaudible”] pis ben on peut pas non plus en vouloir aux psychiatres, ils sont sous l’eau, ils font ce qu’ils peuvent #00:21:39-1#

MF2: oui [pause], oui. #00:21:39-6# #00:21:41-6#

MF1: Mais bon, voilà ils sont pas assez nombreux #00:21:42-7#

MF2: Mais je pense c’est la disponibilité #00:21:43-2#
MH2: Pi bon la proximité #00:21:44-1#

MF1: Et pis après c’est vrai que je compte aussi beaucoup, je compte aussi beaucoup sur les psychologues [acquiescement de MH2 “hum”] #00:21:46-6# mais c’est vrai que pour mes patients c’est quand même une #00:21:50-0#
MF2: la question du remboursement #00:21:50-3#

MF1: charge financière qui est quand même importante. [acquiescement de MH1 “ouais, pis la… ouais”] #00:21:53-5# j’en sais quand même beaucoup qui le font, malgré tout. Malgré le fait qu’ils aille pas des revenus mirobolants et pis que ça leur coute cher, parce qu’ils en sont contents, [acquiescement de MF2 “hum hum”] parce que ça les aide. Mais c’est vrai que ça me fait toujours un peu mal au coeur de me dire “non mais franchement, je veux dire euh, y a des trucs abracadabrant qui sont payés qui coutent un bras, et pis euh… là [rire étouffé de MF2]… Non mais c’est vrai! Et pis là euh… Il, il, il leur faudrait, je sais pas, un pauvre mille francs pour entreprendre quelque chose de vraiment sérieux. Là “non euh… écoutez…” Une fois j’avais entendu un argument comme quoi, s’ils payaient de leurs poches, ils allaient mieux adhérer au travail sur eux même… J’veux dire quand même j’étais un peu là “pouuuf… Ouais bah ça faut le dire vite”. Enfin bref, donc ouais c’est… J’pense de ce point de vue-là nous on a des bons psychologues. Moi y a des psychologues en lesquels j’ai vraiment confiance [acquiescement de MH1 “hum”]. Mais c’est vrai que je suis toujours un peu heurté au côté financier, je trouve que c’est pas très très juste quoi. Franchement, ni pour le psychologue, ni pour le patient, je trouve que c’est pas très correct quoi. #00:22:54-1#

MH2: La, la proximité, je pense c’est quand même une bonne chose, parce que ça veut dire que les, les patients qui sont chez nous en suivi, ben… Ils habitent assez proches. Ils se déplacent facilement chez nous, mais après de leur dire “il faut aller jusqu’à Chaux-de-Fonds” suivant qui ça peut aussi être une barrière. #00:23:04-6#

MF1: Chaux-de-Fonds, c’est [inaudible], t’es gentil hein… #00:23:06-4#

MH2: et pis ouais #00:23:06-6#

MF1: Mais c’est plutôt Neuchâtel #00:23:08-2#

MH2: Ou Neuchâtel. #00:23:08-6# [acquiescement de MH1 “hum hum”] Donc euh… Voilà, alors je sais pas. C’est vrai que on… J’sais pas, [inaudible] on nous a envoyé beaucoup de

psy— psycho-gériatrie ? Moi pas beaucoup. C'est beaucoup des jeunes, j'ai envoyé à CL-Psy. #00:23:15-9#

MF1: À CL-Psy ? Non moi aucuns, parce que de toute façon, toute la psycho-gériatrie que j'ai, je fais avec l'équipe de psychologie. #00:23:19-7#

MH1: Donc euh, ouais on en envoie pas. C'est vrai on envoie que des jeunes. #00:23:21-9# Enfin,… J'ai l'impression, j'en ai peut-être une ou deux mais… Très peu. #00:23:25-8#

MF1: Moi, moi je me suis vraiment, je me suis vraiment basé sur la psychiatrie adulte #00:23:26-4# … #00:23:27-3#

MH1: ouais c'est vrai. #00:23:26-5#

MF1:…Donc euh. #00:23:27-2# Mais pour la psycho-gériatrie… #00:23:28-8#

MF2: C'est quoi, soixante-cinq ans ?#00:23:29-9#

MH2: Ouais [inaudible] #00:23:29-3#

MF1: L'âge AVS ouais. #00:23:30-8#

MF2: Ouais. #00:23:32-0#

[pause] #00:23:33-3#

MH2: Ah on en a quelques-uns mais très peu. #00:23:34-1#

MF2: Nan, c'est surtout des… Des adultes. #00:23:37-6#

MH2: Donc ouais, ils peuvent quand même se mobiliser plus facilement déjà, c'est vrai. C'est pas un argument force. #00:23:40-6#

MF1: Bon ça dépend, certains ils sont pas… Enfin, ils ont pas beaucoup de ressources, quand même hein. #00:23:43-1# [acquiescement de MH1 “hum hum”]. #00:23:42-0#

Mais c'est ça, y en a qui ont pas beaucoup de ressources, qui vont par exemple, pas le permis de conduire, pas la voiture, et pis du coup c'est quand même pratique [acquiescement de MH1 “ouais”] de pouvoir venir… [acquiescement de MF1 “hum hum”] #00:23:51-4#

MH2: Ici quoi. #00:23:52-1#

MF2: Jusqu'ici #00:23:52-6#. Et pas d'aller… #00:23:54-8#

MF1: Et pis c’est vrai que voilà hein, si y a fait des, des choses qui prennent beaucoup de temps qui étaient à entreprendre, pour ça, CL-Psy elle l’a toujours fait [acquiescement de MF2 “hum hum”] même si c’était pas les tâches euh… Ben justement comme cette patiente qu’a du être hospitalisée, elle a, elle a fait tous les [acquiescement de MF2 “ouais”], tous les papiers [acquiescement de MH1 “hum”], elle a téléphoné à [inaudible], elle a appelé l’ambulance, elle a tout fait euh… [acquiescement de MF2 “hum hum”] #00:24:14-8# Ouais c’est des choses…

MH2: ouais ça te décharge bien. #00:24:17-6#

MF1: Ben ouais. [acquiescement de MH2 “ouais”] C’est des choses, si moi j’avais du le faire euh, ça m’aurait pris la demi-journée. #00:24:22-6#

MH2: Ouais. #00:24:22-9#

MF2: On peut ouvrir, mais [Intervention de MH2 “y fait chaud”] j’ai peur que ça fasse trop de bruits [MH2 en même temps: “trop de bruits hein”] #00:24:26-4#

MF1: Ouais pour le…. #00:24:26-5#

MF2: On essaye ou? #00:24:27-9#

MH2: Oui. [intervention de MF1 “wauuhh…”] #00:24:30-5# Si ils déchargent pas, [inaudible] #00:24:32-8#

MH1: Si, si on revient un peu sur la question que on…Que vous avez déjà parlé avant #00:24:41-4# C’est la question de comment vous collaborer, concrètement ? Comment vous, vous… Comment se passe la démarche, comment… Vous communiquez, concrètement ? #00:24:51-2#

MH2: Alors… #00:24:51-9#

MF2: Ben ça commence en général par uuuune lettre [acquiescement de MH1 “hum hum”], parce que… Rien que pour des questions administratives. Donc en fait, quand nous on voit un patient qu’on veut lui adresser, on lui fait un petit courrier, mais… En trois lignes [acquiescement de MH1 “hum hum”], on lui explique la situation, on lui met les antécédents, les traitements. Pis comme ça euh… Ellll— elle à tout ce qui faut d’un point de vue administratif, pis elle sait un petit peu de quoi il en retourne #00:25:17-9#

MH2: C’est vrai que dans un monde idéal, ça aurait été bien qu’elle ait accès à notre euh… #00:25:20-8#

MF2: Ouais qu’elle ait accès directement à nos… #00:25:22-9#

MH2: à notre dossier #00:25:23-0#

MF2: … À nos dossiers. #00:25:23-3#

MH2: Comme ça elle pourrait lire nos… [acquiescement de MH1 “hum”] Nos notes [acquiescement de MF2 “ouais”], et nous on pourrait lire les siennes. #00:25:25-8#

MF2: Exactement. #00:25:26-7#

MH2: Ca serait beaucoup mieux. #00:25:26-6#

MF1: Moi ce que je faisais, c’est que j’écrivais une lettre, pis je reprenais les dernières consultations. C’était hyper vite fait avec le programme [acquiescement de MH2 “hum hum” ; acquiescement de MF2 “ouais”] #00:25:32-1# Et pis du coup, elle pouvait voir un peu ce que j’avais noté, alors euh… Je lui disais hein “tu regardes pas trop, parce que des fois on note des choses un peu vite fait ou comme ça”. Mais elle me dit ça lui donnait quand même un peu une idée [MF2 en même temps: “une idée”]. Aussi de la fréquence [acquiescement de MH2 “ouais”] des dernières consultations etc. [acquiescement de MF2 “hum hum”] #00:25:42-8# Donc euh moi souvent euh, je faisais, j’mettais les, les quatre-cinq dernières consultations. #00:25:46-5#

MF2: Mais en gros c’était ça, c’est qu’on lui fait un courrier [acquiescement de MH2 “hum hum”]… Euh. Enfin, on… Comme c’est nous qui pouvons prendre un rendez-vous euh, dans sa consultation, #00:25:54-0# bah on prenait le rendez-vous.

MH2: Pour un premier, j’crois… #00:25:55-0#

MF2: Pour le premier. [acquiescement de MH2 “ouais”] On prenait le rendez-vous, pis on lui mettait une lettre… Euh, comme ça elle savait euuuuhm… Quel patient, pourquoi elle le voyait. Et pis après, bah justement euh… on… Communication plutôt informelle autour de la table du midi. #00:26:10-3# Ou… Dans le couloir #00:26:11-9#

MF1: Sauf pour moi, où on me demandait beaucoup pas mail ou par téléphone #00:26:14-2#

MH1: hum hum. #00:26:14-2#

MF2: Mais même moi hein, y a des… Parce que, comme elle vient que une fois par semaine et pis que elle est à l’autre bout du couloir, des fois on ses voit même pas pendant toute une journée, donc euh… Y a… Même, [acquiescement de MF1 “hum hum”] même si je suis là le même jour qu’elle [acquiescement de MF1 “hum hum”], y a aussi de la communication par mail. Euhm… #00:26:29-1#

MF1: Mais ça, je trouvais pas que c’était du tout un frein, pas du tout. Pas du tout #00:26:34-8#

MF2: Ah, non pas du tout. #00:26:35-6#

MF1: Ca allait très bien #00:26:36-5#

MH2: Non, ça communique euh… [acquiescement dans ce sens de MF1 “non non”] Du moment que ça communique, on est déjà contents. #00:26:40-3#

MF2: Ouais. #00:26:41-1#

MH1: hum hum. #00:26:41-2#

MH2: [rires] J’crois que … [intervention des 4 donc inaudible] — pause. #00:26:44-5# Ben parce que, bah y a quand même avec des spécialistes ou… Avec qui y a pas de communication [acquiescement de MF2 “ouais”]. On sait pas quels traitements [acquiescement de MF2 “ouais”] ils ont changé… #00:26:54-6# Ce qu’ils ont trouvé…

MF1: Pas que les psychiatres mais… #00:26:56-2#

MH2: Non je parlais… Je disais moi, c’est pour ça que je disais des spécialistes [acquiescement de MF2 “ouais”]. Mais en gynéco [acquiescement de MF2 “ouais”] , en rhumato, on a régulièrement [rire étouffé de MF2] le problème. #00:27:01-9# Un rhumato [MF2 en même temps: “un rhumato”. puis rires]. Et puis en gynéco on a régulièrement le problème, on sait pas quels traitements ils ont changé [acquiescement de MF2 “ouais”], qu’est-ce qui ont mis, qu’est-ce qu’ils ont vu, [acquiescement de MF2 “ouais”], euh… Donc du moment que y a déjà une communication [acquiescement de MF2 “hum hum ”] on est déjà contents. #00:27:10-6#

MF2: Ouais, c’est déjà vachement bien. #00:27:11-7#

MH1: Est-ce que le fait que, que… À quoi cette constellation joue un rôle sur le fait que y a peu de communication #00:27:17-9# À quoi vous pensez ? [peu audible] #00:27:18-9#

MH2: Ah bah c’est peut-être personnel. Peut-être qu’elle elle est réactive à ses mails et pis qu’elle euh… Qu’elle le fait volontiers, peut-être que ça c’est du à elle, je sais pas. Ou c’est dans son cahier des charges. #00:27:28-5#

MF1: Moi je pense que ça doit quand même être dans son cahier des charges, parce qu’il me semble euhm… Que le docteur S. il avait pas mal insister sur le fait que justement il fallait qu’il y ait …euh #00:27:34-7#

MF2: De l’intervision, supervision… [peu audible] #00:27:35-1#

MF1: …Un échange euh… Ouais entre les deux praticiens qui suivent le même patient [acquiescement de MF2 “hum hum”], justement pour essayer d’améliorer sa prise en charge. Moi, de ce que j’avais compris du projet [acquiescement de MH2 “hum hum”, acquiescement de MF2 “oui”], y avait quand même un peu… Enfin, comme dit Sarah, qu’était assez central. #00:27:45-5#

MH2: Et c’est vrai que ça peut être en dehors de la journée de consultation. Mais après d’un point de vue purement financier, elle peut quand même, comme elle a le patient, elle peut

quand même facturer du temps en absence quoi. [acquiescement de MH1 “hum hum”]. Ca c’était une chose qu’on avait discuté [acquiescement de MF2 “hum hum”]. Parce que ça va, ça lui prendre du temps hein [acquiescement de MFH1 “hum” ; acquiescement de MF2 “hum hum”]… De répondre à tous ces mails si y a trois cabinets euh. Ca fait douze médecins qui lui … [bruitage] #00:27:59-6# [acquiescement de MF2 “hum hum”] — rires de MH2. Eh mais même si on se dit j’envoie que un, un mail toute les deux semaines, elle elle en reçoit tous les jour en fait. #00:28:04-6#

MF2: Ouais je pense, ouais. #00:28:05-9#

MH2: Quand on est comme ça, on se rend pas compte mais… [acquiescement de MF2 “hum hum”] Ouais… #00:28:07-1#

MF2: Ouais. #00:28:07-9#

[pause] #00:28:08-6#

MH1: Hum, hum. Et le retour. Qu’est-ce que vous avez comme retour de consultations ? #00:28:13-8#

[pause] #00:28:13-7#

MF1: Moi souvent très positifs. #00:28:16-8#

MH1: Hum hum. #00:28:17-7#

MF1: Mais j’ai quand même une patient qui m’a dit “mais j’aime mieux discuter avec vous, Doctor. J’ai plus l’habitude [rires étouffés de MH2 et MF2] #00:28:25-2#

MH2: Non mais ouais, tout positif hein. [acquiescement de MF2 “ouais”] #00:28:28-8# Moi je, j’ai pas eu trop de… De mauvais retours au fait. [acquiescement de MF2 “hum hum”]. #00:28:34-4#

MF1: Enfin, moi les gens ils étaient très optimistes. Ils étaient vraiment contents. Ils trouvaient vraiment que ça les avait vraiment aidé à ce, à cet euh.. à ce moment de leur vie euh. [acquiescement de MF2 “hum hum”] #00:28:45-8# [acquiescement de MF2 “hum hum”] … [inaudible] #00:28:48-1#

MF2: Mais y en a qu’une ou… Ca s’est pas bien passé mais… Voilà, après, j’ai.. j… j’ai p’tetre pas, j’aurai peut-être pas du l’envoyer. Mais c’est une patiente, ben justement de la gériatrie du coup, [rires] vu qu’elle a soixante-sept ans. Mais… Elle est plutôt petites fleurs, petites huiles euh… Talismans, pendules mais, elle a quand même, elle avait quand même un traitement psychotropes. Et pis ça, ça se passait pas très bien. Pis je me suis dis “mais, vu sa longuuue histoire psy, ben peut-être que ça serait bien de demander à CL-Psy, juste, de la voir, une voire deux fois pour faire le point sur euh, sur les médicaments. Pis elle est allée la voir, pis elle aurait du retourner la voir, pis finalement elle a annulé, parce que finalement elle a trouvé quelqu’un qui fait euh… #00:29:31-4#

MH2: Les énergies. #00:29:33-0#

MF2 : J'sais pas quelles énergies. [rires de MF2 et MH2] Et pis voilà que, que, elle avait pas envie qu'on touche euh… Enfin, qu'on touche aux médicaments [acquiescement de MH2 "hum hum"]. Que finalement elle a tout arrêté mais… C — c'était la seule patiente avec qui euh, ben y a eu une interruption vraiment très précoce et pis, que ça a pas croché. Mais j'pense que parce que c'était pas le genre de patiente qui adhérait euh, à ce genre de suivi. Mais… #00:30:03-8#

MH2: J'en ai une qu'a arrêté parce que y avait du retard. #00:30:06-8#

[rires de MF2] #00:30:07-9#

MH2 [rires aussi]: C'est vrai que voilà… #00:30:10-4#

MF2: Parce que toi t'es toujours à l'heure. #00:30:11-3#

MH2: Elle, elle, elle est, elle est… pas. On a tendance à être à l'heure nous, parce que,… Enfin. Généralement hein, on a tendance à avoir, un quart d'heure, une demi-heure de retard, pis là y avait passé une heure et pis euh, du coup elle a même pas fait le premier rendez-vous. Pis après, [acquiescement de MF2 "ouais"] j'ai pas réussi à la remettre [acquiescement de MF2 "ouais"] dans le, le truc. #00:30:28-4# Donc après, voilà, pour cette patiente là, je me suis dis "ben, faut qu'on prenne le premier rendez-vous du matin ou de l'après-midi pis on aura pas de problèmes quoi." [acquiescement de MF2 "hum hum"] #00:30:37-1# Voilà, j'ai juste eux ça. #00:30:38-5#

MF2: Mais, non. Moi, en tout cas les, les patients euh ils sont toujours suivi. OU alors ils ont été aiguillés quelque part et pis euh. Pis ils vont mieux. J'en ai une en tête qui va vraiment, vraiment mieux. Alors euh… Moi je trouve plus plutôt [acquiescement de MH2 "ouais"], ils sont reconnaissants, ils sont satisfaits et pis euh… Mais ils aiment bien revenir vers nous [rires]. #00:31:00-8# [acquiescement de MH1 "hum hum"]

MH2: Mais c'est vrai que quand ils, que eux ils se sentent en détresse, qu'ils ont un problème, qu'ils veulent nous voir, pis qui, qui aimeraient voir un psychiatre, ou qui sentent que ça suffira pas juste ensemble. Leur dire "ah ben dans deux mois ou trois mois y aura quelque chose", ils … Ils ont pas l'impression qu'on les prend [acquiescement de MF2 "hum hum"] au sérieux [acquiescement de MH1 "hum hum"]. #00:31:12-9# Et pis que si là on dit on "ah ben organise ça, on l'a fait, vous avez votre rendez-vous, il est là, hop dans deux semaines vous la voyez". Là ils se disent "ah bah mon médecin il m'a prit au sérieux, il voit ma détresse" [acquiescement de MH1 "hum hum"]. Ouais pis ouais je pense que ça c'est aussi bien quoi. [acquiescement de MF2 "hum hum"] #00:31:24-5#

[pause] #00:31:26-0#

MH1: Et les restitutions qu'elle vous fait elle. Vous faites une restitution, comment ça se passe ? #00:31:30-4#

MF1: Justement elle fait des rapports [acquiescement de MH1 “hum hum”] #00:31:32-5# [suite inaudible]

MF2: Ouais. #00:31:32-8# Mais elle fait #00:31:34-3#

MH1: Moi ça manque de temps. #00:31:34-4#

MF2: Moi j’ai reçu qu’un seul rapport pour une prise en charge qu’était terminée [acquiescement de MF1 “hum hum”]. Donc euh, enfin j—justement elle avait, elle avait finalement envoyé à l’hôpital de jour, donc elle [acquiescement de MF1 “hum hum”] l’a suivait plus, pis à ce moment-là j’ai reçu le retour. Mais sinon, pour les patients qui sont en cours [acquiescement de MF1 “hum hum”] de prise en charge, j’ai que des retours euh, informel, oraux… #00:31:53-3#

MH2: C’est, c’est vrai que je me dis si c’était, si elle faisait un peu comme un avis de sortie de l’hôpital quand ils sortent euh, d’une hospitalisation après [acquiescement de MF2 “hum hum”] quatre séances par exemple [acquiescement de MF2 “ouais”]. Avec diagnostique, traitement et pis propositions thérapeutiques ou qu’est-ce qu’elle va faire, sans avoir toute un… [acquiescement de MF2 “ouais”] bla-bla. Eh bah ça nous aiderait ouais, [acquiescement de MF2 “hum hum”], je pense. #00:32:08-5#

MF2: Hum hum. #00:32:08-9#

MH2: Ca nous prendrait pas trop de temps. #00:32:10-9#

MF1: Mais ouais moi je pense que j’ai, enfin je pense que j’ai plusieurs chances, parce que justement elle sait qu’elle me voit pas. [acquiescement de MF2 “ouais”] #00:32:14-6#

MH2: Du coup elle t’en fait… #00:32:16-2#

MF1: Parce que du coup, ouais…. #00:32:17-8# J’ai presque les patients [peu audible] #00:32:19-2# … des rapports

MF2: Ouais, mais là on a reçu euh… #00:32:20-2#

MH2: Ok. #00:32:21-2#

MF2: Parce que S. avait envoyé aux différents cabinets de groupes pour savoir euh, tu sais y avait eu cette réunion à laquelle on avait pas pu aller [acquiescement de MF1 “hum hum”], quand t’étais à l’hôpital. Et pis, il nous envoyé le retour [acquiescement de MF1 “hum hum”], et pis y Chantal Simon d’Oberson [vérifier l’orthographe] qui a répondu que globalement, ben ils étaient super contents mais, que, par rapport à ces… Rapports euh ben… Ce — ils estimaient aussi que, y avait pas besoin de faire [acquiescement de MH2 “hum hum”] quatre pages recto-verso. Que… Diagnostic, traitement, prise en charge… #00:32:55-4#

MH2: C'est vraiment comme le, ouais [acquiescement de MF2 "ouais"], les hospitalisations, avec un avis de sortie euh #00:32:57-8#

MF2: Donc je pense que y a, y a pas que pour nous qu'a'est comme ça. #00:32:59-2#

MF1: Ouais. Après je pense que pour euh, les dossiers des patients et tout ça, même simplement d'un point de vue assez curologique, peut-être faire une synthèse complète par exemple justement quand le patient a plus besoin de suivi, ou quand il est adressé à une autre structure qui va avoir besoin de, de, de tous les éléments du statut psychiatrique etc. que elle seule peut leur donner. J'pense que la c'est, c'est important #00:33:17-6# d'avoir un rapport #00:33:18-6#

MF2: Pour le dossier. #00:33:17-9# Pour le dossier je pense. #00:33:19-8#

MF1: Mais du coup effectivement pour euh, pour euh… Juste…
#00:33:21-2#

MF2: Mais pas pour le médecin traitant. #00:33:22-3#

MF1: Voilà. Pour euh, juste une espèce de petite ébauche pour après deux séances. Dire "bon ben voilà, j'ai vu ça, ça, j'pense, je m'oriente plutôt vers ça. On va débuter un truc comme ça, si tu peux faire des tests hépathiques dans deux semaines ça serait parfait [acquiescement de MF2 "hum hum"] pis un ECG". Ca suffit largement [acquiescement de MF2 "hum hum"]. Mais je pense que voilà, elle pourra malheureusement pas complètement euh, abandonner ces grands rapport parce que, bah j'pense que pour #00:33:40-8# [inaudible]

MF2: [peu audible] C'est pour le dossier. Et pis pour le dossier du patient, ouais. #00:33:43-2#

MF1: hum Hum #00:33:43-5#

MH1: Hum. #00:33:45-2# [pause] Et avec, vous arrivez à aborder des questions, des inquiétudes, des estimations pour vos patients ? #00:33:50-6#

MF1: Ah, oui complètement. [acquiescement de MF2 "hum hum"] #00:33:55-1#

MH2: Ouais c'est là que je reviens sur l'idée qu'elle arrive aussi à, pis c'est ça qui m'avait étonné à, à aborder ça avec le staff quoi. Aussi avec les assistantes qui ont pu beaucoup se livrer à elle, quand elles avaient des problèmes professionnels, et puis, euh, ça été vraiment une ressource quoi [acquiescement de MF2 "hum hum"], pour, pour le cabinet [acquiescement de MF2 "hum hum"] globalement. [acquiescement de MF2 "hum hum"] Pour les patients, pour nous, et pis pour tout le staff. J'ai trouvé que [acquiescement de MF2 "hum hum"]… [suite inaudible] #00:34:20-8#

MF2: Non, pis elle est impliquée, parce que bah voilà, on a vraiment eu des coups durs, entre le départ de notre ex-associé, l'hospitalisation de Louise, ensuite maintenant l'hospitalisation de Simone. Euh, elle a toujours … [MF1: qui doit surement lui faire un geste - parle mais inaudible] #00:34:36-7# [rires] Oui, c'est…[rires de MF2 et puis rires de MH2] une bonne série. Euh… #00:34:42-9#

MF1[rires] : Mais on est bien maintenant [rires de MF2 et MH2] #00:34:44-5#

MF2: Mais … On discute, pis "ah ben elle est pas Louise, ou elle est pas Simone ?" "Ben non, elle est à l'hôpital." Ah pis tout de suite "ah est-ce que tu pense que je peux l'appeler, prendre de ses nouvelles ?" Enfin… Elle est. #00:34:56-7#

MF1: Ce qu'elle a fait ? #00:34:58-4#

MF2: Ce qu'elle a fait ? Elle, elle, elle est impliquée, elle se sent… Enfin, elle… #00:35:03-5#

MF1: Elle se sent partie, j'pense… #00:35:05-5# [suite inaudible]

MF2: Elle se sente faisant partie…. #00:35:07-2#

MH2: Ouais c'est ça. #00:35:07-5#

MF2: …Du, du, cabinet. Faisant partie de l'équipe et pis du coup… Du coup ça se ressent, nous de notre côté, on se sent à l'aise avec elle et pis je pense que j'peux parler au nom des assistantes que c'est le cas pour elles aussi. Pis ben justement, face à cette accumulation de… [pause] D'ennuis qu'on a eu ces derniers temps, y en a pour qui ça été vraiment difficile, pis elles ont pu, euh… Entre deux, entre deux trucs discuter ensemble et pis euh, décharger un petit peu leurs fardeaux. Pis CL-Psy elle était là, elle écoute [acquiescement de MH1 "hum hum"]. #00:35:38-3# Et pis euh…

MH2: De bons conseils #00:35:40-4#

MF2: De bons conseils et… #00:35:41-5#

MH2: Parce que c'est vrai que nous elles nous demande des fois les assistantes si on sait pas exactement [rires de MF2] comment les conseiller [acquiescement de MF2 "ouais"] c'est vrai. Et quand on a eu, quand CL-Psy les a conseillé après, ça nous… [acquiescement de MF2 "ouais"] Nous aussi ça nous fait du bien [acquiescement de MF2 "hum hum"]. Parce que… J'… Ouais, [acquiescement de MF2 "ouais"] j'ai l'impression que… [pause] Qu'on aurait pas été si efficace qu'elle quoi, [rires de MF2] dans ces cas-là. [rires] Euh… #00:35:59-3#

MF1: Non. #00:36:02-2#

[pause] #00:36:04-6#

MF1: Pis bon, par rapport aux patients aussi, moi j'ai trouvé assez surprenant de voir que quand moi j'avais des difficultés à communiquer avec une personne, que j'avais de la peine

à, à entrer vraiment dans le vive du sujet, j'en avais parlé euh, plusieurs fois avec elle, j'pense à une patiente en particulier. Et ben euh, c'est, c'était— c'était, c'était rassurant quelque part de voir qu'elle se heurtait aux mêmes difficultés. Donc euh, que, que peut-être c'était aussi quelque chose en lien avec la personnalité de la patiente et puis pas avec ma façon de faire, [acquiescement de MF2 "hum hum"] de mener un entretien ou quoi que ce soit que. Mes difficultés, l'impression que c'était vraiment dur, c'était pas que moi, c'était aussi une autre personne qui avait encore plus l'habitude de mener des entretiens, qui sentait la même chose, et puis euh, c'est vrai que ça aussi c'est très rassurant [acquiescement de MF2 "hum hum"]. On se dit bon ben voilà "je fais pas tout faux" euh… #00:36:49-9#

MH2: Elle sent vachement bien les trucs hein. #00:36:51-6#

MF1: Ah, ouais. [acquiescement de MF2 "hum hum"]. #00:36:53-2#

MH2: Parce que elle pourrait dire "moi ça fait quand même sept ans que j'suis certains patients", euh.. [rires audibles] tout d'un coup elle les voit et elle a senti tout [acquiescement de MF2 "ouais"] de suite le truc quoi. Pis moi il m'a fallu… [rires de MF1] Je lui ai dis "wawouh". Elle, elle a vraiment une [acquiescement de MF2 "hum hum"] capacité de, de sentir les gens, les dynamiques dans les familles euh… euh… J—j'suis, j'suis épatée quoi. #00:37:13-5#

MF1: Ou alors de mettre, l— un… un, enfin alors voilà parce que les diagnostiques, les troubles anxieux, les… Mais les troubles de la personnalité j'trouve que c'est beaucoup plus difficile. Et puis une de mes patientes, elle a mis comme diagnostique "trouble de la personnalité anxieuse-évitante" pis j'étais là "mais oui …" #00:37:27-5#

MH2: Ouais voilà. #00:37:27-9#

MF1: En fait, c'est vrai, c'est exactement ça. [rires de MF2 et MH2]. #00:37:28-5# En fait, voilà pourquoi elle se met tout le temps dans des situations désagréables [rires de MF2]. Parce que c'est vrai, elle, elle réagit comme ça. Elle, elle se met à chaque fois, et c'est une personne adorable, hyper gentille. Mais effectivement, elle a été battue par son mari, maintenant elle est mal traitée par sa fille ainée. Et pis elle se met tout le temps, dans des, dans des situations comme ça, pis je… Ca m'a éclairé [acquiescement de MF2 "hum hum"] #00:37:46-9#

MH2: Mais ouais. #00:37:47-3#

MF1: Je me suis dis "ah oui tiens, c'est vrai…". #00:37:47-7#

MH2: Pis ça, ça …[inaudible] #00:37:48-4#

MF1: "…Je pense que je suis d'accord". #00:37:48-9# [tentative d'intervention de MH1]

MH1: Est-ce que, il y a ... [?? pas compris]votre regard clinique aussi ? #00:37:51-5#

MH2: Ouais, c’est ça. #00:37:52-4#

MF1: Oui, moi ça m’a, ça m’a beaucoup apporté justement [acquiescement de MF2 “hum hum”] parce que, on me disait mais voilà. Pis, pis quelque part, ben oui, ben ça va certainement arrivé encore parce que ben, on va pas non plus pouvoir complètement [acquiescement de MF2 “hum hum”] la changer. Mais… Qu’on pourra peut-être essayer de lui dire “mais bon, pourquoi vous avez réagi comme ça ?” [acquiescement de MH1 “hum”]. Peut-être pouvoir quand même aller plus de l’avant par rapport à cette manière de [acquiescement de MF2 “ouais”] se défendre qu’elle a depuis… Probablement son enfance. #00:38:09-6#

MF2: Ben moi, pareil avec Madame B., quand euh… Quand elle m’a dit “non mais c’est normal que t’en preuve plus, c’est normal que tu t’épuises #00:38:15-2#

MF1: De la personnalité du type trouble de la personnalité [rires] #00:38:16-3#

MF2: parce que c’est typiquement le type de personne qui épuise le personnel soignant. Et pis c’est normal que tu ailles besoin de soutien [rires de MF1 - et intervention mais inaudible] #00:38:21-5# Mais, du coup ça fait du bien de l’entendre, de se dire que c’est pas nous qui faisons tout faux [acquiescement de MF1 “hum hum”] et puis que, on a plus d’empathie et puis, mais que, non c’est, c’est la personnalité qui fait que on, on… #00:38:34-9#

MH2: C’est vrai ça, elle nous permet de mieux comprendre nos patients [acquiescement de MF2 “oui”, acquiescement de MF1 “ouais”], et puis de mieux [acquiescement de MF2 “oui”] mieux, les, les, les soigner aussi quand on a ben… Accès à ça [acquiescement de MF2 “hum hum”], un diagnostic [acquiescement de MF2 “hum hum”] puis qu’on nous expliquait comment il fonctionne. #00:38:46-0#

MF1: Les troubles de la personnalité, moi je trouve c’est le pire, c’est souvent là où c’est le plus dur. #00:38:49-7#

MH1: Et comment ça modifie votre inter-relation ? Ce changement, ce changement de règles ? #00:38:54-7#

MF1: Moi, j’trouve ça me donne plus de patience aussi. #00:38:56-5#

MH2: Ouais! #00:38:56-6#

MF2: Ouais! #00:38:56-8#

MH2: On est peut être plus compréhensifs. #00:38:58-4#

MF2: Mais c’est ça… On se dit, “ouais il fait pas exprès pour m’énerver quoi, c’est juste que… [rires] #00:39:04-0# [rires de MF1]

MH2: C'est son mode de fonctionnement qu'est comme ça #00:39:06-4#

MF2 [rires et encore rires de MF1]: C'est son mode de fonctionnement #00:39:06-8#

MH2: [inaudible] … De notre fonctionnement #00:39:08-6#

MF1: Ouais. Je vais pas lui lancer cet objet lourd à la tête aujourd'hui… [rires de MF2 et MH2] #00:39:12-1# Je vais attendre encore un peu. [rires de MF2 et MH2] #00:39:16-7#

[petite pause] #00:39:18-1#

MF1: C'est vrai que des fois, on tourne en rond et pis de, d'avoir un autre, justement, un diagnostic, bah oui, des fois on avance pas beaucoup plus, mais on comprend [acquiescement de MF2 "oui"] au moins pourquoi on tourne en rond…. #00:39:31-5#

MH2: C'est ça #00:39:32-1# [acquiescement de MF2 "oui"] #00:39:32-1#

MF1: …Et puis on se dit "bon ben voilà, c'est peut-être pas si grave de tourner en rond, après tout". #00:39:36-6#

MF2: Et pis on apprend aussi que cette personne on pourra probablement pas [acquiescement de MF1 "ouais"] l'aider, enfin, l'aider oui, mais pas [acquiescement de MF1 "non"] la soigner. Et pis juste, l'accompagner et pis euh… #00:39:49-3#

MH2: C'est comme le patient qui a tout le temps mal, qui se plaint quand [inaudible] on trouve pas ce que c'est. Pis le jour où on a un nom sur ce qu'il a, on l'a pas soigné, on l'a pas guéri [acquiescement de MF2 "ouais"] mais il sait ce que c'est et puis du coup il supporte mieux sa douleur [acquiescement de MF2 "ouais, hum" ; acquiescement de MH1 "hum"]. Ben des fois j'ai l'impression que c'est un peu pareil quoi [rires]. Une fois qu'on a mit un nom sur euh… Ce qui a ou sur sa façon de fonctionner, ça va pas l'aider forcément beaucoup mais on, on, on comprend mieux [acquiescement de MF2 "hum hum"] le truc, on arrive à intellectualiser et pis euh [acquiescement de MF2 "hum hum"]… Ca se passe mieux après. #00:40:10-0#

MF1: Mais non mais c'est vrai hein, moi j'ai été malade mais le jour où on m'a donné le diagnostic [acquiescement de MF2 "ouais"], j'étais quand même contente hein. #00:40:14-2#

MH1: Ben ouais. #00:40:14-3#

MF2: Oui. #00:40:14-9#

MF1: Franchement, même si y avait rien à faire de plus hein, mais…[rires] #00:40:16-7# C'était quand même bien. C'était un bon moment. #00:40:20-0#

MH2: Ouais, c'est sécurisant quoi. #00:40:21-7#

MF2: Oui. #00:40:21-7# [acquiescement de MH1 “hum”] #00:40:22-1#

MH2: Un peu, de se dire “ah mais [acquiescement de MF2 “ouais”]… Voilà, il font ça comme ça… #00:40:25-4#

MF1 & MF2 (ensemble) : Hum hum. #00:40:25-9#

MH2: …Réagira comme ça… #00:40:29-0#

MF2: Ouais pis qu’ils le font pas exprès, que c’est pas dirigé contre nous, que c’est leur mode de fonctionnement, c’est leur personnalité #00:40:34-2# Et pis…

MH2: Et peut-être la façon dont j’avais de lui parler était fausse [acquiescement de MF2 “ouais”], puisque il y a cette façon d’être [acquiescement de MF2 “hum hum”], fallait peut-être que j’ai, j’ai d’autres clés, que je, je, je… J—je fasse, enfin je lui montre les choses différemment peut-être, puisqu’il fonctionne euh, [acquiescement de MF2 “hum hum”]… Enfin je sais pas, je pense à un hypocondriaque ou comme ça, peut-être que [acquiescement de MF2 “hum hum”]… Une fois qu’on le sait ben, on se “Je… J’vais essayer de changer ma pratique” … Mais à part ça, ça a rien à voir donc voilà. Mais euh… moi je me suis, enfin c’est juste pour nous, mais je me suis aussi dit que d’avoir une pharmacienne une fois qui vient voir les médicaments [acquiescement de MF2 “hum hum” ; [acquiescement de MH1 “hum”]… Parce que alors des fois… pfou… [acquiescement de MF2 “hum hum”] Alors des fois, on a … L’état, enfin on pis on se dit, “je dois faire du tri”. Mais des fois y a des aberrations dans nos traitements, [acquiescement de MF2 “hum hum”], des choses qui sont inutiles, ça serait tellement [acquiescement de MF2 “hum hum”]… Bien. Bref… #00:42:11-6#

MH1: Ouais. #00:42:12-6# [rires de MF2]

MH2: Mais si vous voulez faire après… Un psy on peut mettre aussi un pharmacologue [rires de MF2]-clinicien qui voit nos consulte [acquiescement de MF2 “ouais”] — rires de MH2 #00:42:19-1#

MF2: Ouais #00:42:20-1#

MH2: Mais y a des fois à l’hôpital #00:42:23-8#

MH1: Ouais #00:42:24-1#

MF2: Oui. #00:42:24-4#

MH2: Et [acquiescement de MF2 “oui”] en ambulatoire pas [acquiescement de MF2 “ouais”] et… J’pense que y aurait beaucoup à redire… #00:42:28-2#

MF2: Oui, oui. #00:42:29-2#

MH1: hum hum… #00:42:29-1# Et…. Hum… Dans les situations complexes, vous avez parlé de quelque chose de complexe, est-ce que… La collaboration avec le psy, est-ce que ça change… De comment vous vivez la situation finalement ? Vous ? Votre vécu ? #00:42:49-4#

MF2: Moi je me sens… J'me sens un peu allégée. [acquiescement de MH1 "hum"] J'me sens… Souvent, dans ces situations complexes, ben c'est dur pour le soignant aussi, c'est, c'est [acquiescement de MH1 "hum hum"] pesant, c'est lourd euh. Du coup, on se sent moins seule [acquiescement de MH1 "hum hum"] et pis euh.. [acquiescement de MH2 "ouais"] c'est quelqu'un avec qui on peut partager et pis… Avec qui on, on peut se sentir soutenue [acquiescement de MH1 "hum hum"] donc euh… Oui, ça fait du bien. #00:43:15-4#

MF1: Ouais. Moi j'pense à une chose, c'est que, les médecins qui font des burn-out, parce que y en a énormément, après j'sais pas si c'est le burn-out dépression, mais y en a énormément. Bah moi, j'pense qu— alors y a le côté privé, le côté familial. Mais dans le côté professionnel, les, les situations qui nous marquent le plus, c'est pas forcément un cancer qu'on doit annoncer ou comme ça, c'est souvent des situations psy qui nous, qui nous reste et pis le patient qui va pas mieux, pis qu'on, l'on emporte à la maison, on sait pas comment faire [acquiescement de MH1 "hum hum"]. Parce que les autres choses, c'est assez clair. [acquiescement de MF2 "hum hum"] Alors oui il a un cancer du pancréas, oui c'est triste, oui c'est… Mais on sait que voilà, il a rendez-vous chez son chirurgien, c'est, sa chimio, nous on va l'aider comme on peut pour le soulager au mieux. Mais dans les situations psy, c'est souvent celles-là qu'on reprend à la maison. Et puis je pense que c'est elles qui pourraient nous tirer en bas et nous, nous, nous, nous faire partir de fois en burn-out ou comme ça [acquiescement de MF2 "hum hum"]. Et pis d'avoir un psy ici et pis de savoir qu'on peut lui, lui [acquiescement de MF2 "hum hum"] transmettre nos problèmes, pis nos, nos patients qui, où on sent que ils vont nous épuiser, et bah pour nous même je pense que c'est super quoi. #00:44:03-1#

MF2: Hum hum. Ouais c'est une ressource. #00:44:04-8#

MH1: Ouais. #00:44:06-4#

MF2: C'est.. Moi c'est une ressource, j'ai une situation en tête, alors c'était pas avec CL-Psy mais un autre psychiatre. Mais la… Elle a fait une tentative de suicide et pis euh… Bah, c'est moi qu'elle a appelé quand elle a f — passé à l'acte. Et pis euh, ben parce qu'elle m'a appelé on a — elle a, elle a pu être sauvée, mais heureusement j'étais pas toute seule [acquiescement de MH1 "hum hum"]. Parce qu — heureusement y avait un psychologue, y a avait un psychiatre aussi dans la course parce que, moi je me serais sentie responsable. Que là je me dis "ben là on était quand même trois et pis on a pas réussi à…. À l'aider euh, j'suis pas seule responsable". Donc ça, ça nous déculpabilise aussi, j'pense. [acquiescement de MH1 "hum hum"] Donc euh, dans ces situations lourdes euh, moi j'trouve hyper important d'être épaulé. Parce qu — d'ailleurs ben cette même patiente, après qu'elle ait fait a tentative de suicide pis qu'elle ait été hospitalisé à PF, ben son psychiatre et a psychologue lui ont écrit une lettre pour lui dire qu'ils pouvaient plus la suivre. Et pis que je me retrouvais toute seule avec euh, avec elle, ben j'peux vous dire j'ai flippé. [rires] Elle venait de faire sa tentative de suicide et son psy— chiatre et sa psychologue la laissaient tomber, moi j'étais là

“ben on va vite vous retrouver quelqu’un parce que… [rires et rires de MH2]” J’étais pas bien [rires]. Et pis ben on a retrouvé quelqu’un que je trouve vraiment très bien. Et pis ben, avec lui, c — c’est vraiment [pause] euh… Ben y — bon j’arrive pas à le joindre [rires] quand je veux l’appeler, mais, mais y a un retour, y des réseaux, on se voit, c’est… Donc euh, ouais ça fait du bien. On se sent vraiment moins seule. #00:45:55-8# [Pause] Des situations lourdes, ça va… [acquiescement de MH1 “hum hum”], On arrive à gérer mais… #00:46:03-3#

MH1: hHum hum. #00:46:04-8# Et par rapport aux projets en soi ? #00:46:07-5# Est-ce que…

MF2: Au quoi ? #00:46:09-3#

MH1: Par rapport au projet. #00:46:10-0#

MF2: Au projet ? #00:46:10-6#

MH1: Au projet en soi, le projet de [inaudible] [acquiescement de MH2 “hum hum”], le projet d’installation dans le cabinet, groupe du psychiatre, est-ce que, par rapport à l’offre existante [pause], quels sont le bénéfice ? Par rapport à l’offre déjà existante avant ? #00:46:22-4#

MF2: Ah ben… [soupir de MH2] #00:46:23-5# Monstrueux! #00:46:24-7#

MH2: Y avait quasi pas d’offres. #00:46:25-3#

MF2: Y avait pas d’offres. #00:46:27-2# Fallait quémander… Fallait, fallait être dans les bons papiers du psychiatre pour éventuellement pouvoir lui refiler un nouveau patient mais… Pis le CMP, bah c’est pas qu’il a mauvaise presse, mais comme les médecins changent, les patients ils ont pas toujours envie de, d’aller dans une telle structure. #00:46:42-3#

MH2: Pis toujours ce délai. #00:46:43-0#

MF2: Pis le délai. #00:46:43-9#

MH2: Le CMP il disait “vous nous envoyer une lettre et pis on répond”. Mais encore une fois, c’est entre deux et trois mois. [acquiescement de MF2 “ouais”] Alors on pouvait les envoyer d’urgence [acquiescement de MF2 “ouais”] ils ont une consultation urgente. Mais euh, non ça c’est vrai que … #00:46:56-0#

MF2: Non mais ça a tout changé. #00:46:57-0#

MH2: En fait, ça, ça, ouais, ça a vraiment remplacé une offre qui, enfin ça pas [acquiescement de MF2 “ouais”] remplacé ça, c’est une, une nouvelle offre qui existait [acquiescement de MF2 “ouais”] pas, j’crois. [acquiescement de MH1 “hum hum”] #00:47:03-3#

MF2: Ouais. #00:47:04-5# D’ailleurs là euh, ben elle a diminué son taux d’activité, pis elle nous a dit [intervention de MH2 inaudible] que parce que en plus elle doit malheureusement

quitter le projet d’ici c’te automne. Pis elle nous a demandés de, si possible, ne plus lui adresser de nouvelles situations parce qu’elle aimerait finaliser ce qu’elle a commencé. Pis le jour où elle nous a dit ça, je me suis retrouvé nez-à-nez avec un patient et j’me suis dis “beeeen [rires] je l’adresse à qui maintenant ?” #00:47:35-6#

MH2: Mais pour dire, euh, j’sais pas si t’as fais exprès ou pas [rires de MF2] mais quand on avait, on a dit qui restait, parce qu’on a encore une place dans, au cabinet, dans notre cabinet. T’as dit “ah tiens, on pourrait demander à CL-Psy si elle veut pas s’installer chez nous” [acquiescement de MF2 “hum hum”]. En dehors du projet finalement [acquiescement de MF2 “ouais”], de dire “c’est tellement nécessaire [acquiescement de MF2 “oui”] ce projet [acquiescement de MF2 “oui”] que on était presque prêts à dire [acquiescement de MF2 “ouais”] ben finalement, à la base, c’est, c’est réservé pour des généralistes ici, enfin [acquiescement de MF2 “ouais”] c’est un accord qu’on avait un peu avec la commune. Mais toi t’étais prête à dire “on va vers un psychiatre”… #00:47:57-9#

MF2: Ah mais… #00:47:57-3# Ben d’ailleurs… #00:47:58-5#

MH2: …Parce que on voit q— #00:47:58-7#

MF2: …je le pense encore très fortement [acquiescement de MH2 “ouais”] aujourd’hui [acquiescement de MH2 “ouais”] #00:48:00-3# parce que… #00:48:00-6#

MH2: C’est ça. #00:48:01-6#

MF2: Alors on a besoin de quelqu’un pour remplacer celle qui est partie. Clairement, on a besoin de généralistes. [acquiescement de MH2 “hum hum”] Mais on a encore un bureau [acquiescement de MH2 “hum hum”] de libre. Moi franchement euh, déjà ça diminuera nos charges et pis euh, accessoirement c’est une offre dont on a besoin. #00:48:15-2#

MH2: Mais c— Ouais ça montre vraiment le, l’importance du truc quoi. #00:48:17-6#

MF2: Ouais. [acquiescement de MH1 “hum hum”] #00:48:18-1# Non franchement quand S. nous a approché [acquiescement de MH2 “ouais”] pour nous demander si on était intéressés par un tel projet, j’crois qu’on était unanimement d’accord [acquiescement de MH2 “oui”] que… Que oui on avait besoin de ça. [acquiescement de MH2 “ouais”] Donc euh… #00:48:28-9#

MH1: Est-ce qu’il y a des points moins positifs ? Ou moins pertinents ? #00:48:31-4#

[Pause] #00:48:33-4#

MH2: Ben moi c’était juste le, le fait de, de cette facilité d’accès de faire attention aussi à pas transférer… #00:48:39-4#

MF2: Envoyer tout et n’importe quoi #00:48:40-9#

MH2: c'est une solution [? pas bien compris] de facilité quoi. De, dès que le patient nous dit "voilà j'ai un petit souci" - "ah, j'ai une psychiatre qu'est dans un cabinet, j'vous donne son adresse, euh, bonne journée" et pis je m'en lave les mains. [acquiescement de MF2 "hum hum"] Ca faut faire attention [acquiescement de MF2 "hum hum"] y a cette facilité là où on pourrait, [acquiescement de MF2 "ouais"], on pourrait tomber pis on fait gaffe en se disant "non mais on, on va réserver vraiment les cas qui nous pose problème [acquiescement de MF2 "hum hum"] et pis… Le reste on prend en charge nous quoi" [acquiescement de MF2 "ouais"]. Donc ça, c— c'est à nous de nous réguler un peu pour ça. Ou elle de nous le dire "écoute celui que tu m'a envoie [intervention de MF2 "mais…"] là… " #00:49:28-4#

MF2: …Moi ce que je me rend compte, c'est que y en a quand même quelqu—e uns, voire pas mal pour qui j'ai fais ça. Mais c'était tout des patients de notre ex-collègue [acquiescement de MH1: "hum hum"] Ils n'avaient plu de médecin traitant… #00:49:36-9#

MH2: Ils ne les connaissaient pas à la base et pis… #00:49:37-9#

MF2: que je ne connaissais pas [acquiescement de MH2 "ouais"] pis que, on je pouvais pas assumer— le suivi [acquiescement de MH1 "hum"]. Et pis du coup, je me suis dis "ça tombe bien, parce que on a cette psychiatre [acquiescement de MH1 "hum"], qui peut, du coup, faire ce suivi-là. Mais pour mes propres patients, je crois q— ouais j, j'ai pas eu forcément la… La délégation plus facile. C'était plutôt pour les patients euh, de Valérie. #00:49:57-2#

MH2: Les points à améliorer, c'est ceux qu'on, dont on a parlé y avait… Ben le retour [inaudible] [acquiescement de MF2 "ouais"] et puis euh, peut être une consultation commune, ça pourrait quand même être bien. Mais faudrait qu'on la mette — combien de temps, est-ce que c'est une demi-heure qu'on fait euh… #00:50:07-1#

MF2: Mais ça c'est, tu peux le faire hein. #00:50:08-1#

MH2: Ouais mais, peut-être qu'on devrait le faire d'office. De dire, première consultation on le fait avec elle. Non? #00:50:12-4#

MF2: Moi je pense ça dépend, de cas en cas, du patient [acquiescement de MH2 "c'est vrai"], de la situation euh… Parce que elle elle est ouverte à faire toute les consultations #00:50:15-5#

MH2: Oui j'sais. #00:50:19-3#

MF2: En binôme hein si il le faut. #00:50:19-6#

MH2: Ben ouais. #00:50:19-9#

MF2: Donc euh… #00:50:21-2#

MH2: J'ai pas si c'est de la gêne, ou si j'me dis [rire léger de MF2], si, si c'était obligé, ben je le ferai volontiers pis comme ça si [bruitage de MH1] ça allait pas, je lui demande pas, j'sais pas #00:50:27-3#

MF2: j'sais pas. #00:50:27-6#

MH2: Mais j'pense ça ça serait bien. Et puis, [barbouille un peu, inaudible]. #00:50:30-2#

MF2: Moi j'trouvais bien que, qu'on ait encore un peu plus de temps à, à partager en dehors des consultations. Parce que ben entre son agenda et le nôtre, ben j'te dis y a des fois, des jours où on se voit cinq minutes à midi pis c'est tout. [acquiescement de MH1 "hum"] Que… Comme elle vient qu'une fois par semaine, euh, ben des fois on a pas assez de temps pour vraiment échanger tout, tout ce qu'on aurait voulu échanger. Donc euh, j'pense que ouais, soit de faire deux jours par semaine au cabinet et pis ou alors… Bah on avait aussi c'te possibilité de, de bloquer du temps pour de l'intervision [acquiescement de MH2 "hum hum"] ou supervision mais [acquiescement de MH1 "hum"], sur une journée de consult euh… Ca fait, du coup ça limite très vite le nombre de consultation qu'elle peut faire. #00:51:26-4#

MH2: Mais c'est vrai que si on bloquait un quart d'heure ou une demi-heure dans la journée ça serait peut-être mieux. Parce que là tout d'un coup nous on est entre deux patients, [acquiescement de MF2 "ouais"] on est juste sorti pis tac elle nous attrape [acquiescement de MF2 "ouais"] et elle nous dit "j'ai vu ton patient". Alor déjà faut se souvenir [rires de MF2] qui c'était. Y a un peu du monde, donc au niveau euh… #00:51:38-7#

MF2: ….Confidentialité… #00:51:39-4#

MH2: au niveau secret prof— confidentialité, c'est— pas terrible. On s'sent pas très bien pour lui répondre parce qu'on sait que y a des gens qui sont là au Desk et pis, et du coup c'est pt-être, entre deux portes [acquiescement de MF2 "ouais"] c'est pas forcément le plus confortable. [acquiescement de MF2 "ouais"] Peut-être qu'on devrait avoir, c'est vrai dans nos—mais ça c'est peut-être aussi à nous de [acquiescement de MF2 "oui"] l'organiser hein. Faut pas qu'on soit non plus dépendants de tout le temps [acquiescement de MF2 "ouais"] que des gens nous disent. Peut-être nous on pourrait se dire "ben okay, chacun on bloque un quart d'heure [acquiescement de MF2 "ouais"], on parle personnellement avec elle [acquiescement de MF2 "ouais"]. Un quart d'heure chaque fois qu'elle vient [acquiescement de MH1 "hum"]. de lui demande si c'est quelque chose qui serait envisageable, j'sais pas. #00:52:04-0#

MF2: …Mais tu vois, ça veut dire que, si nous on bloque [acquiescement de MH2 "ouais"] un quart d'heure #00:52:06-3#

MH2: Ca lui fait une heure. #00:52:06-6#

MF2: Elle ça lui fait une heure. #00:52:06-8#

MH2: Ouais ça lui fait… #00:52:07-7#

MF2: Sur une journée. #00:52:08-1#

MH2: Deux patients en moins, ouais. #00:52:09-6#

MF2: Ouais non trois parce que euh… #00:52:11-1#

MH1: Ouais mais, mais… #00:52:12-2#

MH2 & MF2 (en même temps): Louise est pas là. #00:52:12-9#

MH1: …Si on reste dans le proposition, à cette proposition d'amélioration, d'amélioration de projets ou de développement, d'autres développement, vous imagineriez quoi ? Qu'est-ce qui — ? #00:52:21-1#

MH2: En vrai, y avait juste encore, ça m'est revenu [acquiescement de MH1 "oui"], pour les améliorations du projet, cette idée aussi que moi j'étais pas très au clair de, de sa fonction [acquiescement de MH1 "hum hum"], si c'était orienté [acquiescement de MF2 "ouais"], diagnostiqué, nous, nous, nous re—référer le patient assez rapidement [acquiescement de MF2 "hum hum"] pour des prises en charges brèves. Y avait juste ça. Après euh, j'sais pas — #00:52:39-3#

MF2: euh… #00:52:40-1#

MH2: Euh, bon. Alors peut-être, parce que euh—CL-Psy donc, quand elle est arrivée, parce que le projet était tout nouveau, on a cru qu'elle serait là à vie [rires de MF2]. Mais euh, on est un peu naïf. Parce que on savait bien que c'était une assistante et pis, au chef de Clinique et pis que finalement, ils sont pas chefs de Clinique à vie donc euh… Ben ouais [acquiescement de MH1 "hum"] mais ça c—c'est pas possible pour vous. Pis on s'en rendu bien compte… #00:53:02-2# Mais on aime bien [inaudible] … des personnes #00:53:03-4#

MF2: Bon, à priori… #00:53:03-6# À priori, elle aurait du rester plus longtemps. Mais c'est parce que sa formation n'a pas été reconnue #00:53:09-8#

MH2: Qu'elle doit faire dans un hospitalier [pas compris] #00:53:11-8#

MF2: Que, elle doit partir. Donc euh. #00:53:14-6#

MH2: Ouais mais je sais pas, dans ces projets-là, c'est plutôt un assistant ou un chef de Clinique qu'est ?— Chef de Clinique. #00:53:17-9#

MF2: Ouais, chef de Clinique. #00:53:19-0#

MH2: Pis donc c'est… #00:53:19-8#

MF2: À voir … #00:53:20-5#

MH1: … [inaudible] Alors que y a quelqu'un qui reste c'est ça un peu ? #00:53:22-5#

MH2: Non mais si ils restent deux ans, [acquiescement de MF2 “ouais”] j’pense que c’est déjà bien. Là c’était une année ? #00:53:25-6#

MF2: Elle aura fait une année. #00:53:26-4#

MH2: Elle aura fait une année. #00:53:28-4# C’est peut-être un tout petit peu court j’ai l’impression [acquiescement de MF2 “ouais”]. Pour les patients qui l’auront vu, et pis pour nous. #00:53:32-6#

MF2: Pour nous. [Rires] #00:53:33-5#

MH2: Une année ça fait — #00:53:34-3#

MF2: Aussi. #00:53:34-3#

MH2: Si je, je m’imagine dans dix ans, est-ce que j’aurai vraiment l’énergie à mettre chaque fois que [acquiescement de MH1 “hum hum”] y a un nouveau qui vient, pis je sais que neuf moi après il est loin quoi. #00:53:43-0# [acquiescement de MF2 “hum hum”] C’est une question que j’me pose hein [acquiescement de MF2 “hum hum”] . J’espère que je l’aurai et pis que je serai pas autant égoïste que ça durant — De toute façon, il est de passage, j’vais pas trop m’investir. C’est vrai que si on parle de deux ans, trois ans [acquiescement de MF2 “hum hum”], ça nous fait peut-être plus envie aussi d’investir la personne, la relation. Après voilà, j— #00:54:01-7# C’est vrai que #00:54:03-8#

MF2: Ben après ça dépend aussi de la personnalité, parce que CL-Psy [acquiescement de MH2 “oui”] j’crois que le deuxième mercredi qu’elle était là, c’est bon hein, [acquiescement de MH2 “ouais ouais”] on était copains. [rires] C’est aussi une personnalité qui fait que,… #00:54:14-6#

MH2: …Qui parles volontiers #00:54:16-2# qu’est très ouverte, non jugeante #00:54:18-7#

MF2: … Que ça s’est super bien passé très rapidement, donc euh. #00:54:19-7# Mais… Ouais, une année j’trouve que c’est un — peu court [MH2 dit “un peu court en même temps] #00:54:26-8#

MH1: Alors un projet de trouver des gens sympathiques, alors #00:54:31-0# [Rires de MF2 et MH2] #00:54:31-3#

MF2: Si possible ouais. #00:54:32-8#

MH2: Mais moi j’imagine que si c’est des gens qui sont, qui ont déjà, qui on envie d’être en contact avec les généralistes [acquiescement de MH1 “hum hum”] et ben ça se passe déjà bien. #00:54:43-3#

MF2: Ah ben oui #00:54:44-4#

MH2: Parce que c’est des gens [acquiescement de MF2 “ouais”] qui, qui, qui on envie de nous aider [acquiescement de MF2 “hum hum”], qui, qui voient ce que c’est notre métier, et pis… #00:54:49-8#

MF2: Qu’on envie de collaborer. #00:54:50-4#

MH2: …Qui on envie de collaborer. Pis ça se passe bien [acquiescement de MF2 “ouais”]. Si c’est quelqu’un qu’on place. Et pis que lui il veut faire de la psychiatrie pure et dur en hôpital, ben ça va être long pour lui [acquiescement de MF2 “ouais”] et pis on va le sentir et pis ça va pas être bien. [acquiescement de MF2 “ouais”] Mais quelqu’un qui vient déjà proactif [acquiescement de MF2 “hum”], “ah moi ça m’intéresse [acquiescement de MF2 “hum hum”] d’être dans un cabinet de généraliste, voir leurs préoccupations, quels patients ils vont me référer [acquiescement de MF2 “ouais”]”. c’est déjà gagné. J’pense, ouais. #00:55:13-3#

[Pause] #00:55:16-1#

MH1: Très bien. Et par rapport aux, aux offres supplémentaires pour vous ? Les aspects psychologiques à votre pratique encore ? #00:55:25-6#

MF2: Ben que le projet pilote deviennent un projet… #00:55:27-1#

MH2: Définitif. #00:55:28-0#

MF2: Définitif. #00:55:28-8#

MH1: Oui, oui, ça j’ai compris. Mais la question peut-être un peu plus formation, [inaudible] intervention ? #00:55:32-0#

MF2: Ben justement. [intervention inaudible de MH1] J’pense c’est un peu plus de, s’octroyer vraiment du temps, du… J’sais pas, une fois par mois ou comme ça, pour euh, pour élaborer un peu plus. Enfin de se retrouver, médecins, généralistes et psychiatres euh [acquiescement de MH1 “hum”], et pis discuter… #00:55:49-2#

MH2: Moi, j’reviens à la consultation commune, j’suis désolé [rires de MF2] mais… [rires] #00:55:53-3# Mais euh c’est peut-être parce que je l’ai pas vécu là, mai ça me permettrait aussi euh, des fois de voir comment elle fait pour débloquer un truc, comment elle fait [acquiescement de MF2 “hum hum”], quels outils elle a, comment elle—elle aborde la chose pour que—moi je me suis toujours heurté à un mur, pis elle elle arrive à trouver la brèche. #00:56:02-8#

MF2: C’est cinq ans de formation. #00:56:04-2#

MH2: Ouais. #00:56:05-1# [rires de MF2] Mais voilà, ça peut #00:56:06-6#

MH1: Du coup, vous auriez besoin de plus de formation, vous aussi ? #00:56:07-6# comme un psy ?[pas bien compris] C’est une question qui se pose ça. #00:56:09-8#

MF2: Moi je crois. Franchement, un généraliste euh— il, enfin moi j—j'le vois parce que j'ai pas fais de psychiatrie, mais j'crois un généraliste il doit avoir fait un peu de psy dans, dans sa carrière quoi. Toi t'en a fait. #00:56:20-9#

MH2: oui, non mais j'suis d'accord, oui, oui. #00:56:22-2#

MF2: Enfin, je ne sais pas faire un statut psychiatrique c'est— pppp…[bruitage avec la bouche] #00:56:26-6# Incapable. Donc euh, moi j'trouve que ça devrait faire partie de, de la formation d'un généraliste. #00:56:36-4# Parce que encore une fois, y a combien de pour cent de notre consultation qui est d'ordre psy. Et pas, pas que somatique, donc euh, c'est bien joli de, d'être euh… De connaître toutes les guidelines de médecine interne mais… Si on a pas un petit background de psychiatrie euh… #00:57:03-4#

MH2: Mais c'est vrai que à la PMU, moi ça m'a été hyper utile quand, y a, y avait le psychiatre de liaison qui venait pendant les consultations avec moi. Et pis qui était cash et qui me disait "mais t'as vu ta consult ? [rires de MF2] Tu fais fausse route". Il faut noter un quart [pas bien compris], il faut parler "la dernière fois, vous avez tant de temps et pis que vous aller aborder si, ça". Et pis la fois d'après, il venait et pis il me dit "ah ben j'ai vu que t'as, t'as réussi à mettre en pratique ce que je t'ai dis". Et pis, et pis ça m'a beaucoup stimulé, [acquiescement de MF2 "ouais"] et pis ça m'a beaucoup apporté. [acquiescement de MF2 "hum hum"]. Alors c'est pas du tout agréable sur le moment. [rires de MF2] Mais euh, finalement— #00:57:30-9#

MF2: Ca t'apporte quelque chose. #00:57:31-0#

MH2: … ça, c'est un pied au fesse [acquiescement de MF2 "ouais"] qu'est super. [acquiescement de MF2 "hum hum"] Et c'est pour ça que dans c— dans que— dans que, que là si on peut faire des choses comme ça avec elle, peut-être qu'on pourra aussi progresser sur [acquiescement de MF2 "hum hum"] certains, certains… Thèmes. #00:57:40-6#

MF2: Après si tu veux venir au Balint [??] avec nous, tu peux aussi hein. #00:57:43-2#

MH2: Ouais, mais non, j-j… [rires de MF2] #00:57:43-8#

MH1: Proposer le Balint alors ? #00:57:45-6#

MF2: Moi j'pense que le Balint c'est, c'est quelque chose qui devrait aussi faire partie de… [rires] #00:57:50-0#

MH2: Non mais c'est juste. #00:57:51-1#

MH1: Mais ici je dis. À votre groupe. #00:57:52-2#

MF2: Mais pour tous les généralistes? #00:57:56-2# Nous on est deux du cabinet à faire le Balint avec S. #00:57:58-0# Euh, mais… #00:58:01-1#

MH2: Mais c'est juste #00:57:57-0# Mais moi en deux, en trois secondes, j'ai une patiente c'est mon ancienne prof, euh maitresse d'école. Elle est venue avec un mail qu'elle a reçu de son remplaçant, qui lui disait "ben tu vois y a celui-là qu'a fauché la gomme à celle-là, y a celui-là qu'a passé celle-ci" et elle était en pleurs. Elle était en burn-out, elle en pouvait plus. Elle m'a dit "mais tu te rends compte la classe que j'ai". [Pause] Pis... J'ai du réanimer un ami y a trois semaines qu'est décédé. J'avais envie de lui dire "mais c'est quoi ça ?" [acquiescement de MF2 "hum hum"] . Enfin, c'est rien. Mais j—j'me rendais compte que j'devais pas le faire [rires de MF2] parce que c—c'était pas productif dans la consultation. Pis là, je me suis dis "Mais ça j'aurai du l'amener en Balint" ; mais moi je fais pas partie des Balint. #00:58:34-0#

MF2: Tu peux. #00:58:34-9#

MH2: Mais ça j'me suis dis "ça aurait été un cas" #00:58:34-8# Ca m'a dég— tellement gonflé que [acquiescement de MF2 "ouais"], ils se rendent pas compte de, ils font des montagnes avec des détails. Pis toi tu, dans ton vécu [acquiescement de MF2 "hum hum"], t'as des trucs qui sont pires [acquiescement de MF2 "hum hm"]. Mais tu peux pas leur mettre ça à, à la face, j'comprends bien. Mais ça m'a, ça m'a [acquiescement de MF2 "ouais"], ça m'a gonflé quoi [acquiescement de MF2 "ben ouais"], vraiment. J—j'ai pas réussi à, à [acquiescement de MF2 "ouais"], passé par là-dessus. [rires de MF2] J... Ouais c'est des petits trucs, j—j'ai dis "bon...vous, tu" J'sais plus si je la tutoie ou je la voyoie. J'crois que je la vouvoie et elle elle me tutoie quand j'étais son élève. #00:58:57-3#

MF2: Ben oui. [rires] #00:58:57-6#

MH2 [rires]: Donc j'ai dis "vous avez pas l'impression que c'est peut-être l'accumulation de petites choses [rires de MF2], qui a, au fur à mesure du temps, commence à prendre un petit peu trop de place"— #00:59:10-0#

MF2: Il est bien hein [rires]! #00:59:13-1#

MH2: Ouais, mai j'me mets, ouais mais c'était pas, c'était pas— #00:59:18-1#

MF2: C'était pas sincère [rires] #00:59:20-2#

MH2: Elle aurait pu sentir que c'était pas [rires] sincère. #00:59:24-6# Mais j— j— non, [acquiescement de MF2 "ouais"], j— voilà. #00:59:29-4#

MF2: Mais moi aussi ce matin j'— #00:59:33-9#

MH2: Ben ça pour la Balint ça aurait fait du bien de mettre ça sur la table, j'pense #00:59:37-8#

MF2: Moi aussi ce matin j'en ai eu une comme ça avec une orteil qu'elle s'est fait opéré et pis que ça évolue pas aussi rapidement qu'elle voulait. Pis j'étais là "ouais pis nous y a l'autre qui nous a abandonné, [acquiescement de MH2 "ouais"], elle qui est hospitalisé, l'autre qu'est hospitalisé #00:59:47-4#"....

MH2: …On trouve pas un remplaçant. #00:59:48-7#

MF2: Nous aussi on est dans la M, et pis [rires] “mais oui ton orteil va pas très bien”. [rires de MH2] C’est— Ca fait du bien de relativiser. Ca fait du bien de partager pis, ouais moi j’trouve euh, enfin pourquoi y autant de médecins en burn-out, pourquoi y a remède ? Enfin, moi j’trouve que ça— ça apporte. Alors, nous, j’trouve qu’on a beaucoup de chance parce qu’on a un cabinet de groupe. On est pas tout seul avec notre patient, notre assistante médicale. Et pis que, au-delà d’être collègue, j’crois qu’on est tous amis. Et pis on, on a des occasions de, de décharger, de discuter, de partager #01:00:21-0#

MH2: Tous les mardis, on prend une heure de temps, on discute ensemble. #01:00:21-8#

MF2: Tous les mardis matin on discute. Donc, déjà on a cette chance-là, mais, malgré tout, moi j’fais partie de Balint, alors bon, c’était aussi parce que j’avais besoin de pleins d’heures de supervision pour mon CAS que je n’ai pas fait. Mais euh… #01:00:31-7#

MH2: Non mais t’as continué. #01:00:34-1#

MF2: Mais j’ai continué [acquiescement de MH1 “hum hum”]. #01:00:33-9#

MH2: Elle sait que tu pourras pas faire ton CAS, [acquiescement de MF2 “oui”] donc ça t’apportes vraiment quelque chose. #01:00:35-5#

MF2: Mais j’ai continué. [acquiescement de MH2 “ouais”] Et pis euh, là j’ai eu le dernier mardi soir, où je me disais “ah mais ça m’embête, j’serai tellement mieux à la maison, j’suis fatiguée et tout”. J’y suis allée, c’était avec Vanotti [?? nom de médecin] et pis c’était juste tellement fantastique, j’me suis dis “nan mais, j’ai pas besoin de [acquiescement de MH2 “ouais”] ces heures de supervision, mais ça fait tellement du bien” #01:00:50-4#

MH2: Mais j’pense que le Balint, c’est bien aussi pour du long terme, parce que cette situation-là que tu vas peut-être [acquiescement de MF2 “ah oui!”] revivre une fois ben tu pourras l’utiliser [acquiescement de MF2 “mais oui”]. Moi quand j’commence à saturer, j’prends mon vélo [rires de MF2], j’pars en VTT, pis ça me fait un bien fou. [acquiescement de MF2 “ouais”] Mais je sais que ça, que cette situation-là elle va se #01:01:04-6#

MF2: Elle va revenir. [rires] #01:01:05-9#

MH2: reproduire une autre fois, pis que je devrais reprendre [rires de MF2] mon vélo. Pis que j’aurai pas réussi à la, à la régler. Et donc en fait… [rires] #01:01:12-7# La politique de l’autruche #01:01:15-4#

MF2: On a de la place encore hein mercredi midi. #01:01:16-9#

MH2: C’est pas, c’est pas la mieux. Mais euh… Mais ouais, mais moi j— ouais ouais, [acquiescement de MH1 “hum hum”] J’sais pas, j— j—ouais… #01:01:23-9#

MF2: Réflechis… #01:01:24-1#

MH2: Ouais. J, j— voilà. Je, je sais pas [rires]… #01:01:29-2#

MF2: Y a S., y a A., y a S., V., tu connais… #01:01:35-7#

MH2: Ouais, ouais, ouais, nan, nan, mais c'est, c'est— #01:01:38-6#

MF2: On est un bonne équipe. #01:01:37-5#

MH2: Oui, c'est une bonne équipe. C'est, c'est chouette. Mais j'sais pas si c'est ça dont j'ai besoin, [acquiescement de MF2 "ouais"]. Ou, si c'est justement de me changer la tête mais… [acquiescement de MF2 "hum hum"] On prend un peu des deux [pas bien compris]…

MF2: Mais moi j'trouve ça bien en tout cas. Franchement, pour les, les généralistes qui sommes tellement confrontés aux situation psy, ou psychosociales hein, euh, franchement y a… Y en a des pas tristes. #01:01:54-6#

MH2: Normalement, on a quand même une séance de réseaux une fois par mois, où on peut parler de nos problèmes. #01:01:59-4#

MF2: Ah ouais ? #01:01:59-8#

MH2: Pour moi c'est, ouais. #01:02:00-5#

MF2: Ah, nous on aborde pas du tout ces questions-là. #01:02:02-0#

MH2: Ouais, ça devrait— enfin ils disent que ça pourrait être aussi une place, [acquiescement de MF2 "ouais"] plage ouverte pour parler de nos, de nos doutes, de nos problèmes. Et les assurances demandent qu'on parle aussi des, des erreurs médicales qu'on aurait faites [acquiescement de MF2 "oui"] pour pas que ça se reproduise. C'est, c'est demandé. [acquiescement de MF2 "hum hum"]. Donc euh. Normalement, au début, enfin, ils—on fait comme on veut dans le réseau, mais quand on en avait parlé, ils disaient, au début du réseau, ça pourrait être un moment de plage d'échanges [acquiescement de MF2 "hum hum"], pour voir un petit peu dans notre réseau qui va pas bien euh… [acquiescement de MF2 "ouais"], qui a un problème, qui veut parler d'une situation… #01:02:25-5#

MF2: Mais, y a pas — #01:02:26-1#

MH1: Là y a la présence de psychiatre ? #01:02:26-4#

MF2: Ben c'est ça, y a pas de psychiatres. #01:02:27-6#

MH2: Ouais y a pas de psychiatres …[inaudible]. #01:02:28-7#

MH1: Et est-ce que ce serait intéressant ? #01:02:29-6#

MF2: Parce que moi [acquiescement de MH2 “oui, oui c’est vrai”] c’est ça que je trouve super intéressant. #01:02:31-8#

MH2: C’est juste. #01:02:32-1#

MH1: Ce serait un projet de, de réfléchir à ça [inaudible] … #01:02:35-3#

MH2: Ouais de se dire, on participerait au réseau la première demi-heure et pis après ben ça… Pas forcément mais [acquiescement de MF2 “hum”]. C’est vrai que ça serait intéressant de mettre un psychiatre dans un réseau. #01:02:39-3#

MF2: Mais moi je trouve que la même manière qu’on fait un cercle de qualité [acquiescement de MH1 “hum”] pour faire partie du réseau, pourquoi est-ce qu’on ferait pas un Balint euh, dans, j’sais pas… enfin que ce soit pas imposé, bien sur que non, parce que y a toujours des réticences #01:02:53-1#

MH1: Même si c’est pas un Balint, une présence du psychiatre. #01:02:54-3#

MF2: Mais, un psychiatre qui soit là, qui,… #01:02:56-5#

MH2: Ouais, parce que on a une pharmacienne, [acquiescement de MF2 “ouais”] mais c—ça nous aide pas beaucoup. #01:03:01-2#

MF2: Ouais pis le psychiatre, j’peux te dire, il voit pas les choses comme nous. #01:03:03-7#

MH2: Pis ça, ça aiderait bien la pharmacienne. #01:03:04-4# Comment ?

MH1: On revient en pharmacienne. …[pas bien compris] #01:03:07-6# [rires] #01:03:07-5#

MH2: Ouais, mais non mais… #01:03:07-6#

MH1: C’est une compétition [rires] [pas bien compris] #01:03:09-3#

MH2: Ouais,… [rires de MH1 et de MF2] Non, mais c’est uniquement parce que le réseau euh, bah c’est les assurances qui le paie [acquiescement de MH1 “ouais”]. Et pis ça doit être cost effective, et du coup ils mettent un pharmacien parce que [acquiescement de MH1 “hum hum”], voilà [acquiescement de MF2 “ouais”] il nous dit quels sont les médicaments les moins chers mais… [rire étouffé de MH1] C—ça nous aide pas beaucoup au cours. #01:03:26-6#

MF2: Non. #01:03:29-1#

MH2: Alors un psychiatre ça serait… #01:03:29-3#

MF2: Alors que le psychiatre, il a vraiment une vision #01:03:30-7#

MH2: Ca serait top ouais. #01:03:31-4#

MF2: Des choses qui, enfin [acquiescement de MH2 “ouais”], moi je l’ai vu avec euh, S., ou avec Vanotti, mais… Tu dis “ah ouais j’avais pas vu les choses sous cet angle-là” [acquiescement de MH1 “hum hum”]. Pis du coup, pouf [acquiescement de MH2 “ouais”], tu, tu, tu te mets dans une autre position [acquiescement de MH2 “ben oui”], pis tu vois les choses différemment. Pis, [acquiescement de MH2 “ouais”], ça décante des situations. #01:03:47-4#

MH2: C’est juste. #01:03:46-6#

MF2: Non franchement euh, merci d’exister. [rires de MH2 et rires de MF2] #01:03:50-6#

MH1: Très bien, mais merci à vous. #01:03:52-7# J’pense qu’on peut s’arrêter là… #01:03:53-8#

Focus Group 2 :

MH1: Alors on va commencer par rapport- par rapport à des questions autour, qui concerne plutôt vos patients, votre Clinique pure si on peut dire un peu ça. Et on se posait la question finalement, dans quelle situation, dans quel cas vous faites appel au psychiatre ? [silence] #00:01:22-0#

MF3 : Euh, faire appel au psychiatre euh. Dans les cas où on se sent le plus démuni je pense euh, la plus grande sit- enfin la plupart du temps c'est euh, des gens qu'on voit plus ou moins en urgence pis on commence à faire un bout de chemin avec eux et puis on se rend compte que ben voilà la situation est un petit peu plus complexe et nous dépasse un peu ou on va avoir des arrêts de travail qui se prolongent [acquiescement de MH1: "hum"] ou euh… Des- enfin je sais pas. Des- #00:01:58-7#

MH3: -Médications. [acquiescement de MF3: "médications"] Médications qui se complexifient. #00:02:00-9#

MF3: Ouais, qui se met en place et autres. Euh, avec bien sur une grande réticence de ces-ces patients-là [acquiescement de MH1: "hum"] que, en temps normal, on continue à essayer, je crois que c'est un des buts de ce projet, c'est de quand même continuer à travailler avec les autres psychiatres existants et autres et puis si on arrive à les référer plutôt directement plus loin, moi je continue à essayer de le faire disons, de garder- de garder le psychiatre au cabinet dans un contexte euh, un petit peu plus compliqué euh, qui fait [acquiescement de MH1: "hum"] que, ben qu'il est là seulement pour ça, pour les gens d'ici, qu'on aurait pas pu mener chez un psychiatre normal. #00:02:37-7# [acquiescement de MH1: "hum"] Ça je pense que c'est des situations. Euh… Après moi j'ai plusieurs patients euh, voilà, des gros troubles pychiatriques, notamment des problèmes bipolaires et autres euh, avec une poly-médication et autres euh… Où voilà, il faudrait mettre plus, moins et autres où des fois je m'y perds. Alors, c'est vrai que ceux-là ils sont souvent difficile à amener quand même jusqu'au psychiatre. Et des fois là je travaille plus par avis conseils ou choses comme ça [acquiescement de MH1: "hum"] parce que on va pas forcément les voir parce que souvent, eux justement on arrive pas à les aiguiller plus loin [acquiescement de MH1: "hum"]. Et puis rarement, dans l'urgence, ça m'est arrivé une ou deux fois, où voilé, c'était euh, euh, des situations du genre voilà, suicidaires et autres que j'aurai envoyé euh ben, aux service d'urgences [pas compris]. Pis ben voilà, CL-Psy était là juste cet après-midi, elle avait des disponibilités, enfin elle a prit de la disponibilité c'est- pour les voir ensemble. Euh, et puis qui on pu éviter euh… Voilà les hospitalisations, les choses comme ça [acquiescement de MH1: "hum"] enfin, elle a prit, [acquiescement de MH3: "hum"] elle a prit ce relai-là en aigu euh, alors que moi, en tout cas une fois, j'aurai pas voulu gérer moi toute seule en ambulatoire. [acquiescement de MH1: "hum"] #00:03:53-3# Et puis après un patient, voilà, qui a des problèmes [pas compris] de long long terme qui étaient hyper réfractaires à voir un psychiatre que j'ai essayé d'aiguiller et puis je crois qu'il l'a vu une ou deux fois, mais, voilà, la peur du psychiatre est quand même trop importante pis ça s'est arrêté assez vite donc là

on est pas aller trop loin dan ces démarches. [acquiescement de MH1: “ hum hum”] Je laisse la parole à mes collègues, je réfléchis encore [rire général] #00:04:16-6#

MH3: C’est vrai que y a ceux qui- qui, qu’on tient un peu à bout de bras comme ça, avec une partie d’une médication, moi je pense aussi, les médicaments et les interactions c’est, c’est aussi une, une motivation d’aller plus loin. Et l’activité, enfin la question qu’on peut faire sous, sous forme de, de mail ou sous forme d’échanges téléphoniques ou ponctuel comme ça mais ça peut être aussi une prise, une prise en charge très, très ponctuelle comme ça. Le, le coup de pouce un peu. Le, le petit truc où on est à un moment- moi je me trouve confronté à une impasse et j’ai besoin d’un avis pour sortir de cette impasse [acquiescement de MH1: “hum”] Après y a des gens qu’on sent vraiment profondément euh… Nécessiteux d’un, d’un soutien et d’un approfondissement de ce soutien et, et là c’est un bon, un bon moyen de l’adresser pour euh. Mais ça se travaille. Ils sont pas, ils sont relativement réfractaires [acquiescement de MF3: “hum hum”], ils sont pas tous, et puis je sais pas si c’est partout comme ça, mais en tout cas dans les campagnes vaudrusiennes [pas bien compris] euh, les gens ont encore pas mal d’à priori vis-à-vis [acquiescement de MF3: “ouais”] de la psychiatrie quoi, ça reste euh, ça reste les mêmes concepts un peu ancêstraux c’est-c’est [acquiescement de MF3: “hum hum”]. Mais avec une bonne explication souvent ils s’ouvrent. Je pense particulièrement, t’as évoqué un-un ou une patiente [acquiescement de MF3: “hum hum”] moi je pense à quelqu’un qui, qui vient de terminer le suivi avec la doctoresse [pas compris] #00:05:50-4# avec CL-Psy. Euh, j’aurai jamais pensé qu’il collerait dans une, dans une consultation quoi. Mais voilà la proximité à permis de faire ça quoi. #00:06:02-3#

MF3: Moi je crois - je t’interromps mais euh. On discute psychiatre, les gens c’est “non”. Pis après, on peut faire une phrase qui dit “ah mais y a un psychiatre qui travaille ici au sein du cabinet, il pourrait vous voir ici à [pas compris] #00:06:13-9# Et là, “ah oui d’accord”. Ça c’est fou. Ça c’est les choses qu’on a, que si [pas compris]. Pis voilà ça, des fois une ou deux consultations qui permet de, ben voilà de voir le psychiatre un peu autrement. Mais quand même le fait qu’il soit ici, pis qu’on le connaisse et puis qu’il soit- c’est-c’est une porte d’ouverture du “oui”. #00:06:30-2#

MH4: Ah oui, ça c’est un- très très impressionnant. [acquiescement de MF3: “ouais”] Cette euh, cette réaction. C’est vraiment, c’est vraiment une porte ouverte vers la psychiatrie. Là on parle un peu d’autres choses, mais de, dans cette, cette proximité d’CL-Psy, même, même voilà qu’elle soit là une fois, quand on dit aux gens qu’il y a quelqu’un qui vient une fois par semaine euh ici, vous pourriez aussi la voir si vous êtes d’accord. Les, les- ça change complètement. [acquiescement de MH1 et de MH3: “hum hum”] Y a- ça, ça fait tomber le bloc. Le bloc psychiatre ou psychologue euh, j’essaye toujours d’être, être large. [acquiescement de MH1: “ hum hum”] Juste moi, moi je me rends compte comme ça en vous écoutant aussi j’aime bien le mot “démuni”. C’est, c’est d’une part quand je me sens démuni mais peut-être que je le suis assez rapidement, j’ai pas fait de psychiatrie et je sais que j’ai pas de, disons, de, de base de psycho-thérapie et je suis toujours, je suis toujours de l’avis, il y a des patients qu’on arrive à suivre et je sais qu’on peut, qu’on généralise jusqu’à un certain point notre- voilà. On, on a notre poids aussi et pis faut pas, tout, tout ce qui est psychologique ou psychique, plutôt psychiatrique le renvoyer. En, en même temps, qu-quand un traitement médicamenteux dure aussi et pis que, que je vois pas d’évolution ou

bien que le patient n'en ressent pas, et que j'ai l'impression elle ou il a besoin d'aller plus loin dans une euh, pour- pour surmonter un problème ou pour- pour en sortir d'un, là j'aime bien adresser au psychiatre. Ça, Ça c'est en général hein, j'pense pas spécifiquement maintenant à la situation qu'on vit, mais en général au point de vue, au point de vue psychiatre. Et pis les cas plus lourds, moi j'en ai un peu moins eu ces, ces temps des cas vraiment, vraiment lourds de, de psychiatrie disons de voilà - bipolaires qui viennent et qui ont, qui ont, qui font leur, leur grande crise. J'en, j'en avais plus y a une euh, une dizaine d'année [acquiescement de MH1: "hum"] mais… #00:08:25-0#

MF3: Parce que moi j'en ai hérité de pas mal. #00:08:30-2#

MH4: Oui, oui apparement. #00:08:30-8#

MH1: Et- est-ce qu'il y a une situation auxquelles vous penser ? Est-ce que ? #00:08:34-0#

MF3: Par rapport de ? #00:08:36-9#

MH1: Une situation type comme ça ? #00:08:37-1#

MF3: De gros troubles bipolaires ? De- de, par rapport- #00:08:38-2#

MH1: Par rapport de problématique où vous aller appeler le psychiatre ? Qu'est-ce qui vient comme ça comme, comme cas, comme situation concrète ? #00:08:48-0#

MH4: euh… #00:08:49-9#

MF3: Alors moi j'ai une grosse situation actuellement. J'pense que je vais en parler avec CL-Psy, mais j'pense qu'elle pourra pas m'aider. [acquiescement de MH3 [pas sur que ce soit lui]: "ok"] Une dame euh. De gros gros trouble bipolaire qui est à domicile euh, qui a fait de multiples hospitalisations, que j'ai tenu à bout de bras pendant deux ans mais qui a rien autour d'elle parce qu'elle refuse tout. Euh, elle a fini par faire trois mois et demi là d'hospitalisation à Cery. Elle vient de sortir euh, euh, avec en deux semaines euh, plus rien autour d'elle. Donc, elle était censé y avoir des infirmières, des infirmières en psychiatrie, un psychiatre et autres et en deux semaines elle a tout, tout, tout mis en plat. Enfin tout - il y plus personne excepté moi et, et voilà, j'ai passé une heure et demi chez elle lundi ; une heure mardi avec elle et je sais pas très bien ce que je vais en faire. Et voilà. On essaye d'appeler un petit peu "au secours" autour de nous mais il y a personne. Tout le monde - [acquiescement de MH1: "hum"] à part nous dire qu'on peut la renvoyer à l'hôpital. Mais… voilà. C'est pas si facile que ça, y a- euh, y a pas grand monde sur qui on peut compter. Alors ça c'est des choses voilà. Ça se sera un regard extérieur parce qu'elle va pas vouloir l'avoir et puis euh… Enfin la patiente va refuser de voir CL-Psy et autres, mais ben question médications et autres. Parce que là bien sur elle peut arrêter ses médicaments [inaudible] par quoi je pourrai les remplacer, quoi, comment. C'est une situation que lundi je vais prendre un petit moment si elle- euh elle est disponible pour en discuter avec elle, parce que moi je suis, je suis un petit peu perdue là au milieu [acquiescement de MH1: "hum hum"]. Et puis en même temps c'est, c'est toutes ces situations où, quand même en temps que généraliste, on est tout seul. [acquiescement de MH1: "hum"] Les psychiatres euh, moi j'ai

une réponse concrète d'un psychiatre qui me dit "non mais la [pas compris] c'est trop compliqué pour moi je peux pas la suivre en ambulatoire". Ok. Mais au moins vous êtes psychiatre pis vous connaissez un peu mieux que moi- #00:10:40-2# enfin voilà [acquiescement de MH1: "ouais ouais ouais"]. Donc ben y a plus personne, y a nous pis on fait, on fait comme on peut, du mieux qu'on peut disons. Mais euh… #00:10:47-4#

MH4: Pour reprendre la médecine de premier recours et d'ultime recours par rapport à - #00:10:51-7#

MF3: Mais oui. [acquiescement de MH1: "hum hum"] Des fois c'est un petit peu ça. #00:10:54-0# [acquiescement d'MH4: "ouais"] Donc c'est vrai que là je vais remettre en cause, en question ces médicaments, pis ben je vais profiter de cette aide d'CL-Psy. Voir si elle a un conseil à me donner. Bon c'est une situation un peu extrême hein que je vous expose. [acquiescement de MH1: "hum"] Mais c'est… - #00:11:07-2#

MH3: Mais c'est vrai, on a le sentiment d'être - #00:11:08-3#

MF3: Voilà, c'est, c'est mon vécu de cette semaine on va dire ça comme ça. #00:11:10-5#

MH3: - sentiment, j'ai pas, je pense pas particulièrement à une situation donnée. Mais le sentiment d'être pris euh, un peu, en otage. [acquiescement de MH1: "hum"] ou en tout cas euh, d'être prisonnier du, du, du patient. [acquiescement de MF3: "ouais"] Déjà de sa demande, de, de pas vouloir enfin, de, de respecter, on doit respecter ses, ses souhaits et pis on peut pas l'obliger à aller chez un, chez un collègue, quel qu'il soit. Qu'il soit cardiologue ou neurologue ou psychiatre quoi. [acquiescement de MF3: "hum hum"] Donc, euh, c'est vrai que des fois #00:11:43-1#

MF3: Des fois on fait… [soupir, rire étouffé] #00:11:43-5#

MH3: … On fait avec ce qu'on a quoi. #00:11:47-2#

MF3: Ouais. #00:11:47-2#

MH3: D'où l'avantage du cabinet de groupe. [acquiescement de MF3: "hum hum"] on peut partager un peu. [acquiescement de MH1: "oui"] Sans que ça sorte entre guillemet de la maison. [acquiescement de MF3: "hum hum"] #00:11:56-9#

[silence] #00:11:59-4#

MH1: Hum hum. #00:12:01-3# Très bien. Est-ce qu'il y a des fois alors que vous hésiter ? De demander euh, à Madame [pas bien compris], à CL-Psy de l'aide ? Une hésitation vous savez pas est-ce qu'il faut l'appeler ou pas ? [silence] #00:12:19-6#

MH3 : Je pense la proximité géographique permet de, de, de passer cette, cette barrière. [acquiescement de MH1: "hum hum"] De plus hésiter. Je pense que j'aurai plus d'hésitation à, à solliciter un collègue en ville, un collègue dans un, un des centres. #00:12:34-8#

MH1: Pourquoi ? #00:12:39-2#

MH3: Euh… Par téléphone, comme ça peut être la-la… #00:12:41-0#

MH1: Pourquoi ça serait pourquoi ces hésitations ? #00:12:43-3#

MH3: Je sais pas c'est peut-être plus facile de taper à une porte juste à côté que, que de prendre un téléphone, de tomber parfois sur un répondeur parce que voilà. De, de rappeler plusieurs fois ou de, euh… Ouais. [acquiescement de MH1: "hum hum"] J'pense que la proximité géographique et que… [silence] #00:13:05-8# Ça veut pas dire qu'il n'y a pas de disponibilité de la part de, de, [acquiescement de MH1: "hum"] des autres collègues mais- [silence] #00:13:11-9#

MF3: On les connait- enfin là je parle pour moi mais, moi je les connais pas en fait [acquiescement de MH1: "hum hum"] très peu… #00:13:15-3#

MH4: C'est peu hein. #00:13:18-9#

MF3: Par rapport à… tous ces autres réseaux de spécialistes où on communique et on connait, on a un petit peu quand même les spécialistes avec qui [acquiescement de MH3: "hum hum"] on travaille plus, pis on, on peut prendre le téléphone pis les appeler [acquiescement de MH3: "hum hum"]. Le psychiatre c'est plus compliqué parce que c'est vrai qu'on a très très très peu de communication. #00:13:35-3#

MH3: Très peu de contact hein, ouais. #00:13:35-1#

MF3: y a des gynécologues et des psychiatres avec qui ont parle pas beaucoup. [acquiescement de MH3: "ouais"] Enfin ça, c'est toujours ce que je me suis dis. #00:13:42-5# [rires de MH4]

MH3: C'est vrai. #00:13:42-7#

MF3: En tout cas moi, donc j'ai plus de peine à [acquiescement de MH3: "hum hum"] prendre un téléphone, d'interrompre quelqu'un que je connais pas pour… [acquiescement de MH1: "hum hum"] #00:13:49-0#

MH4: Donc, donc à votre question j'aurai répondu d'emblée, comme ça "non, il y a pas de, il y a pas d'hésitation" mais, mais il y a quand même des patients que j'ai pas envoyés ou pas demandés à CL-Psy, mais que j'ai envoyé à un autre psychiatre surtout si c'est des patients qui sont là, qui, qui ont besoin ou j'ai l'impression qu'ils auront besoin d'un long terme. Parce que, je, je la voyais vraiment comme [acquiescement de MH3: "hum hum"], comme voilà u- une aide ou un appui, un complément sûre pour une prise en charge de, plutôt de court terme ou d'orientation ou de… [inaudible] Moi j'ai peu eu l'occasion de discuter directement mais parce que je, quand elle est là je consulte euh, je consulte [pas compris] #00:14:30-3# je l'ai rarement vu ici mais donc pour, de lui dire "mais écoute voilà un problème est-ce qu'on peut, est-ce qu'on peut en parler ?" Vous vous, l'avez un petit peu plus, un peu plus eu ces situations, cette situation. #00:14:40-6#

MH3: c’est pas du tout, c’est, c’est- ça résonne pas avec ce que tu disais avant, où toi c’est plutôt pour des prises en charge plus longues. #00:14:50-6# Non ? #00:14:51-0#

MF3: Huuuum. Non. Si c’est, si c’est des prises en charge plus longues, j’essaye de les aiguiller à l’extérieur. #00:14:55-2#

MH3: Ah, ouais ok. J’ai mal compris alors. Ouais. #00:14:56-1#

MH4: Après les lourdes, voilà. Les très très lourdes, alors là. #00:14:58-8#

MF3: Y a ces gens où, voilà #00:14:58-5# Où tu dis “c’est une première étape” [acquiescement de MH3: “hum hum”]. Notamment y en a une, où ben voilà elle l’aura vu pendant six mois pis maintenant je pense que la patiente est prête à continuer, qu’elle arrive à le remettre plus loin [pas sur, car pas bien compris]. #00:15:07-1#

MH4: Elle- #00:15:10-7#

MF3 : Pis dans ce sens ce sens-là on a réussi. [acquiescement de MH3: “hum hum”], mais… #00:15:12-7#

MH4: Elle a, elle a, elle a baissé un peu une- un une, une- j’sais pas hésitation face à la [acquiescement de MF3: “hum hum”], à la psychiatrie elle a, elle a eu confiance. Mais ça c’est aussi toute la personnalité de, de la personne, du psychiatre en question, ça c’est l’autre chose. On a eu une chance phénoménale moi j’trouve d’avoir une psychiatre qui, est- voilà qui a un très très bon accès pis qu’on, les, la plupart des gens, moi j’en connais pas qui ont pas dit que c’était bien, qui on un bon accès avec lequel elle a eu. Elle a eu un accès. Alors que, ils sont en ville, à l’extérieur ça, ça arrive régulièrement et pis faut aussi attendre que on prépare les patients à ça aussi, en disant “voilà vous essayez. Et pis, et pis c’est comme toujours. Peut-être que vous tombez bien, peut-être que vous tomber mal [acquiescement de MH3: “hum hum”] Et puis la même personne, le même psychiatre pour une autre personne sera, sera la bonne, le bon psychiatre. Et elle est très, voilà. Dans son, dans son rôle-là très très large je dirai, dans, dans, dans l’accueil, dans l’échange et pis après dans sa psychothérapie. Je me suis toujours demandé si c’est en lien avec le type de psychothérapie qu’elle- l’école disons plutôt. Son orientation cognitivo-comportementale plutôt que, que autres. [acquiescement de MH3: “hum hum”] Systémique, freudienne ou je sais pas quoi [acquiescement de MH1: “hum”] #00:16:32-3#

[acquiescement de MH3: “hum”] #00:16:33-5#

[silence] #00:16:34-2#

MH1: Est-ce que vous aurez une situation où vous avez hésité ? Et vous avez dit “Ah tiens, est-ce que je l’appelle ou pas ou est-ce que je gère moi-même ou est-ce que… ” #00:16:45-1#

MF3: Euh… #00:16:49-4#

MH1: “…J’attends ?” #00:16:50-2#

MH4: Ben moi hésiter non, mais par exemple une situation où je n’ai pas fait appel à elle mais j’ai dis à la personne, j’ai pas parlé de la présence d’une, d’une psychiatre ici [acquiescement de MH1: “hum”], quelqu’un qui a un- conflit de couple de longue date et, et que, je, je suis depuis, depuis je sais pas dix-quinze ans et qui très régulièrement a des phases de, de grosse euh, panique, d’angoisse et puis, et puis ça va ensemble avec le couple qui va moins bien mais c’est malgré tout un couple qui tient. Où c’est vraiment du plus longue terme et il est dans une phase où ça va moins bien où je, où voilà il y a pas que avec les médicaments on peut aider et tout mais y- y a besoin de plus et il est aussi d’accord. Là, j’en, j- voilà. Je n’ai pas hésité de ne pas faire appel à CL-Psy. [rires] Pas à cause de ses compétences, mais parce que c’est justement pour, c’est pour illustrer ce que je disais avant. C’est, c’est pour, c’est quelque chose sur le plus long terme qui me, qui me semblait. Où c’était pas pour résoudre juste cette situation. #00:17:51-3#

MH1: [pas compris] fait appeler ? #00:17:53-5#

MH4: Non, j’ai pas [accentue le “pas”] fait appel à CL-Psy, pardon. #00:17:57-3#

MH1: Vous avez fait appel à un autre psychiatre. #00:17:57-1#

MH4: À un autre psychiatre, à un autre psychiatre. #00:17:58-0#

MH1: Mais vous avez fait appel à un psychiatre. #00:17:58-9#

MH4: J’ai fais appel à un psychiatre [acquiescement de MH1: “hum hum”]. Mais pas à elle. Mais pas à cause de ses compétences du tout, à cause de la situation même pour la personne où je me suis dis “voilà” [acquiescement de MH1: “hum hum”] #00:18:06-2#

MF3: Moi je pense où j’hésite des fois c’est- qu’on pourrait avoir la tentation de lui redonner des choses qu’en fait nous on gérerait. [acquiescement de MH1 et MH4: “hum hum”] D’avoir des situations euh, aigues ou pas aigues, compliquées et pis d’avoir cette facilité de se dire “ah ben on a psychiatre ici”. [Réaction de MH1: “ah”] #00:18:25-9#

MH4: Et on avait- #00:18:27-0#

MF3: Donc là, moi je fais vraiment, je- chaque fois je me pose, des fois j’aurai envie de le dire, pis je me dis “non, mais tu peux commencer un bout de chemin, la voir une-deux-trois fois pis, ça reste une situation, pis de pas tomber dans cette facilité de, finalement de, voilà, d’avoir euh, on envoie quelqu’un pour un burn-out #00:18:42-0#

MH3: C’était la question, ouais. #00:18:43-7#

MF3: Ce qui me vient à l’esprit de se dire “ah ben on a un psychiatre ici”, en se disant “non mais y a quand même un bout de chemin que je peux faire, que je peux faire moi”. Et ça me semblait important de justement- que ce psychiatre là ben reste là et pis que ça devienne

pas euh, non plus normal d'envoyer tout le monde tout le temps chez le psychiatre [acquiescement d'MH4: "hum hum"] entre guillemets parce qu'elle est là [acquiescement de MH1: "hum hum"]. #00:19:02-4#

MH3: C'est la question qu'on se posait au départ hein, quand on discutait de ce projet. [acquiescement de MF3: "ouais"] C'était de se dire [acquiescement de MF3: "hum"] que on a une offre est-ce qu'on va pas augmenter la demande quoi, ouais. #00:19:11-3# [pause] Donc on peut avoir des barrières dans les deux sens [acquiescement de MF3: "hum hum"]. On peut avoir des réticences et pis on peut avoir- tomber sur un oreiller de paresse quoi. [acquiescement de MF3: "hum hum"] J'pense. #00:19:23-2#

MH4: En, en même temps moi j'ai une ou deux fois donné, en tout en gardant ça en tête parce que c'était aussi l'autre, juste en ce point de pas vouloir la surcharger en sachant voilà, on est six, elle vient une fois par semaine et pis, et pis elle fait ça. Mais j'ai eu, y a une ou deux situations, où j- je me suis dis après coup "mais peut-être que si elle avait pas été là, j'aurai pas envoyé la personne". Je sentais que la personne avait besoin mais, c'est, j- j'ai utilisé cette facilité quand même avec beaucoup alors à ce moment-là de- parce que c'était pas des problèmes psychiatriques que je dirai profonds ou des grosses pathologies, mais des situations à dénouer quand même où le, le côté ouais, le côté psychothérapie, le côté psychiatre a, a aidé à quand même, à faire avancer, à faire avancer la situation avec, avec quelques euh… Avec quelques séances. En, en cernant aussi très bien voilà, le problème voilà comme on est, voilà comment l'aborder et puis, et puis aller de l'avant. Non, moi je pense, en autres, dans une situation d'un jeune qui, qui avait un problème de genou et, et qui reprenait le travail pendant un jour et demi et puis après une semaine plus tard on avait un téléphone "ouais j'ai besoin de [pas compris] parce que ça fait une semaine que je suis pas retourné" et puis on le revoyait et puis on avait- y a quelque chose qui joue pas et puis, finalement voilà elle, elle a, bien aidé à, à- maintenant il fait tout autre chose, et puis il est dans, il fait un autre apprentissage et puis il est content. #00:20:51-1# [acquiescement de MH1: "hum hum"] Alors j'ai, je l'ai revu, je pense à lui parce que je l'ai revu il y a deux jours avec un problème de bas de dos, douleurs dans le bas du dos. [rires] J'aime bien votre regard [rires]. Mais, mais voilà. Y a déjà tout, tout ce cheminement qui, qui a été fait pis ça c'est, j'pense ça été, ça, ça a bien [pas compris]. J'ai eu, j'ai comme ça une ou deux citations où j'étais, quand même très content et c'est grâce à cette proximité que j'ai, que j'ai demandé parce que, parce que la personne, de nouveau, serait, serait, n'aurait pas bénéficié pis, et puis en a bien bénéficié de cet appui psychiatrique, psychologique. #00:21:28-6# [acquiescement de MH1: "hum"]

[silence] #00:21:33-7#

MH1: Vous m'avez dit déjà un peu, mais, mais si on prend la globalité des patients qui est le compliqué que vous avez envoyé en psychiatrie ou à CL-Psy. Comment résiste le patient ? Mais si on prend- enfin vous dites ce serait bien on a un collègue psychiatre, il reste comment ? Quelle est la première réaction comme ça ? #00:21:56-2# [petite pause]

MH4: C'est avec une courte hésitation de dire "ah dans ce cas-là, oui d'accord je vais essayer". Mais il vienne de nous dire "non". [rires] Ils viennent de- on leur a, c'est-à-dire on, dans la situation je dis "mais je pense que ça serait, ça serait utile que vous contactiez un, un

psychiatre, ça pourrait vous aider parce que le psychiatre, voilà, à une autre formation que nous. Spécialisé là-dessus, je, je souligne un peu ça, pour essayer de, de voilà. De passer outre ce, cette barrière de, de tout ce qui est psy, qui est pas pour moi. Et puis, et puis là, “non, non, je suis pas prêt à, et puis je pense pas que c’est pas la bonne chose pour moi, c’est, c’est une des réponse que je, que je reçois. Et puis, là, si pour moi c’est suffisamment important pour la personne de, de [pas compris] #00:22:56-3# et que je, je lui dis “mais, mais on a quelqu’un qui vient une fois par semaine ici, une, une psychiatre, est-ce que ça pourrait être une possibilité ? Et là l’hésitation elle est, elle est brève. C’est, c’est impressionnant. Les, les gens me disent- alors moi je sais jamais si c’est parce que ils m’ont dit “non” avant et pis maintenant je leur tend une deuxième perche alors ils osent pas dire non mais, en plus c’est, c’est un peu comme un soulagement moi j’ai, j’ai- et j’ai rarement eu une autre, je me souviens pas d’une réaction où la personne a dit non [acquiescement de MH3: “hum hum” ; acquiescement de MH1: “hum”] #00:23:27-4#

MF3: Moi j’en ai plusieurs. #00:23:28-6#

MH4: qui on dit “non” quand tu as?- #00:23:30-0#

MF3: Qui on dit “non” les deux fois. #00:23:31-0#

MH4: Les deux fois, ah oui ? #00:23:31-8#

MF3: En plusieurs qui on dit- qui on e- [pause] pour lesquels ça leur a permis de dire “oui” mais j’en ai quand même beaucoup qui- #00:23:39-7#

MH4: qui disent “non, non”. #00:23:40-0#

MF3: qui restent “non, non”. #00:23:41-0#

MH3: Qui continue à dire “non”, [acquiescement de MF3: “ouais”] Malgré la… #00:23:41-8#

MH4: Ouais. #00:23:43-4#

MH3: …La disponibilité. #00:23:44-3# [acquiescement de MF3: “ouais”] Je suis en train de me demander si, si les gens ont une petite difficulté déjà, à- quand on leur propose de voir des confrères spécialistes, des collègues que ce soit dermatologues ou autres, ben voilà, c’est déjà difficile parce que c’est Neuchâtel, la Chaux-de-Fond si ils ont déjà de la peine à, à franchir un premier pas pour un spécialiste pour un confrère, pour un collègue qu’ils connaissent pas. Et pis après est-ce que y a un autre niveau qui est lié au psychiatre qui, qui serait déjà menuisier [??] parce que finalement c’est juste lié au statut de spécialiste et pis après est-ce que y a une histoire géographique, de, d’aller euh, simplement sans à priori ou sans réticence à aller voir un spécialiste ou un psychiatre mais qui est lié à, à la géographie quoi. Au fait de devoir se déplacer, de se parquer, aller, je sais pas, je sais pas s’il y a des choses comme ça qui sont, qui sont extra-médicales. #00:24:38-8#

MH1: Vous avez l’impression aussi que vous devez le convaincre ? Comme dit … ? #00:24:45-8#

MH3: Si… [pause] Oui. Oui. Oui. Chaque fois que je, je leur propose d'aller chez un- mais, peut-être chez un cas violeur [pas sur d'avoir compris] #00:24:59-2# aussi quoi [acquiescement de MH1: "Hum"], en tout cas, mais c'est peut-être du à ma personnalité, c'est peut-être moi qui, qui veut leur expliquer et puis argumenter pis non pas leur proposer une pilule blanche comme ça à prendre, mais bien leur expliquer que c'est un anti-hypertenseur qui a des effets secondaires qui peuvent, voilà c'est un petit peu, alors de toute façon, probablement encore plus, d'être un peu plus avec le psychiatre, ouais. [acquiescement de MH1: "hum hum"] #00:25:21-8#

MH4: Mais, mais si tu dis à une personne "je pense que ce soit utile que vous alliez chez un cardiologue. Les gens vont beaucoup plus vite dire "oui, ah bon [acquiescement de MH3: "ouais!"] si vous pensez, vous croyez ?" et pis on leur explique [acquiescement de MH3: "ouais"] et pis bon, ils, y vont beaucoup moins… [acquiescement de MH3: "ouais"] Après- #00:25:35-1#

MH3: ouais, moins besoin d'argumenter, ouais. #00:25:38-2#

MH4: Ouais, après si tu prends- #00:25:39-2#

MH3: De, de développer ouais. #00:25:40-7#

MH4: Si tu penses aux, aux gens plus âgés, ça c'est encore- [acquiescement de MH3 et MF3: "hum hum"] ça c'est effectivement qui ont ce frein de plus vouloir trop aller en gros se déplacer ou comme ça #00:25:49-1#

MH3: C'est ça, ouais. #00:25:50-0#

MH4: À gauche à droite #00:25:50-1#

MH3: Ouais peut-être que plus âgés… #00:25:50-9#

MH4: Mais… Mais, mais ce, ce frein pour le cardiologue ou le gastroanthérologue il est, [acquiescement de MH3: "il est moins-"] il est beaucoup moins là [acquiescement de MH3: "hum hum"] que si on dit psychiatre. Et moi, parfois, [rires léger] en leur proposant je, je, voilà, y a aussi les psychologues. Y a psychiatres et y a psychologues, et, et j'essaye de faire la différence. D'expliquer la différence aux, aux gens, [acquiescement de MH1: "hum hum"] quand y a, quand y a moins un problème, disons là, là, thérapeutique, médicamenteux, je, euh, moi j'aime bien aussi proposer un psychologue même si, voilà. On, en leur disant qu'il y a pas de prises en charge, après c'est des questions financières qui peuvent jouer un rôle. Mais aujourd'hui on a d'autres, d'autres possibilités, mais ça ça va changer de toute façon. #00:26:30-4# [légère pause] #00:26:32-2# [acquiescement de MH3: "hum hum"] On a d'autres possibilité à travers les cabinets de, de psychiatres qui ont, qui ont des psychologues, c'est à ça que je pense. #00:26:39-7#

MH1: Mais, mais vous avez l'impression que le recours en psychiatrie, elle va, consulter en un jour pour les relations, entre vous et le patient ? D'appeler, de pas appeler le psychiatre.

De dire le mot “psy”. Est-ce que ça change quelque chose dans votre relation ? #00:27:00-7#

MH4: Moi j’ai pas cette impression, non. Non, on peut en revenir, non. Quand on a une bonne relation avec la personne et pis que- moi j’ai pas cette impression que, que le fait d’avoir mentionné un psychiatre vous ferait du bien ou un psychologue vous ferait du bien, alors là je le, je le mentionne pas comme ça [acquiescement de MF3: “hum”] Mais, [rires] mais j’ai pas trop l’impression. #00:27:24-3#

MF3: Moi je pense quand même que- #00:27:26-3#

MH4: C’est vrai ? #00:27:27-2#

MF3: Que quelques fois, quand on est dans ces espèces de [pas compris] douloureux chroniques et pis qu’on les a déjà envoyé un peu près partout. [Réaction d’MH4: “Ah”] Et puis qu’il nous reste plus rien et qu’on doit commencer à évoquer cette piste [acquiescement d’MH4: “hum hum”], c’est quand même, ben beaucoup plus difficile d’en parler avec eux que de leur dire comme euh, ben de l’envoyer chez un cardiologue ou chez quelqu’un autre. [acquiescement de MH3: “hum hum”] Finalement, il y a tout d’un coup, on a fait un peu tout le reste, il nous reste quand même le volet psychiatrique et on a quand même en face de nous des gens qui sont peu prêts à l’entendre ou peut-être ils l’ont déjà entendu [acquiescement de MH3: “hum hum”] et ils veulent justement pas qu’on leur dise [acquiescement de MH1: “hum”]. Et euh, peut-être moi c’est parce que aussi ben aussi je [acquiescement de MH3: “hum hum”], j’arrive maintenant, en reprenant des nouveaux patients à ce moment-là, peut-être que vous, vous avez plus de [acquiescement de MH3: “hum hum”] d’années dernières #00:28:05-8# qui fait que [acquiescement d’MH4: “ouais”] vous avez passé ce cap. Mais où c’est difficile de leur dire ça. Et des fois ils le prennent mal et on le voit. [acquiescement d’MH4: “ouais”] Enfin, je pense ça peut casser un bout de relation enfin, en tout cas… #00:28:15-7# [acquiescement de MH3: “ouais”] Pis en même temps, ben voilà, on a fait tout le reste et pis c’est aussi notre rôle de les amener là-bas mais… #00:28:21-2#

MH3: J- je m’entend leur dire “mais vous savez, y a forcément, quand on a des douleurs [acquiescement de MF3: “ouais”] et pis voilà. Pis c’est, c’est le début de la négociation, c’est le début de, de l’amener sur un territoire, qu’on sent un petit peu comme ça marécageux, un peu difficile [acquiescement de MH1: “hum hum”], ça va être incertain quoi. #00:28:41-6#

MF3: C’est quand même plus compliqué que, que pour des choses physiques, ouais. #00:28:49-2#

MH3: Oui. #00:28:50-4#

MH4: Mais en même temps, parfois, parfois d’être très directs aide aussi. D’être très direct en disant “écoutez, moi je pense, je pense que vous, que, que on peut, que ça serait, que ça serait chouette pour vous, vous bénéficieriez de voilà. D’une aide, d’un psychiatre ou de l’avis d’un psychiatre”. Mais peut-être que je suis un peu trop dans la théorie maintenant. Mais ça m’est arrivé une ou deux fois où j’étais étonné de la réaction de la personne qui

disait “ah bon, vous croyez?” Là, on- oui, oui, on l’a pas toujours [rire léger de MF3] et pis c’est, c’est un très bon exemple les douloureux-chroniques. C’est, c’est très très bien. Mais de temps en temps très, mais, mais voilà, ça c’est vraiment enfin, au bout de, quand y a une relation qui est là, qui est là depuis, depuis longtemps. [acquiescement de MH1: “hum”] #00:29:32-7# Moi je pense. #00:29:35-9#

MH3: Ouais, je peux quand même [pas bien compris], facilement confiance. #00:29:37-8#

MH4: Ouais. #00:29:38-6#

MH3: Quoi que, quoi que, qu’on leur propose quoi [acquiescement de MF3: “hum hum”] Avec, avec beaucoup de recul. C’est étonnant, c’est déjà une- j’étais étonné en arrivant ici les propositions que je pouvais faire aux patients, euh, et puis euh, qu’étaient rapidement acceptées quoi. Par rapport au milieu hospitalier où c’est différent effectivement. Mais euh, je pensais aussi en dehors des douloureux chronique ou de ces gens. Y a quand même toute une population de patients qu’on va adresser qui ont tout un cheminement où il y a beaucoup de souffrance quoi. Donc cette souffrance à un moment, si on leur propose quelque chose, je pense qu’ils sont prêts à le prendre. Euh, on arrive aussi dans une- alors pour nous t’es dans une impasse, mais pour eux aussi quoi, c’est dire “ok mais on avance un petit peu, mais je veux sortir cout que cout de ça” et l’acceptation là elle est quand même beaucoup plus facile pour cette population-là. #00:30:38-4# [pause] #00:30:42-7# Une souffrance euh, vraiment euh, pathologique ou socio- socio professio- familiale ou comme ça ouais. #00:30:53-2#

[pause] #00:30:54-6#

MH1: Et le bénéfice finalement des patients concernés, est-ce qu- qu’est ce que vous voyiez comme bénéfice finalement ? Qu’est-ce qui- de quoi bénéfice le patient ? De- de la présence d’CL-Psy ? Du fait qu’il- que vous la consulté ? #00:31:12-4#

MH4: Le sourire quand ils reviennent nous voir mais ça c’est moi qui bénéficie [rires]. Parce que quand ils remercient qu’on l’a envoyé et puis d’avoir fait cette démarche et, et parfois en disant même de, de m’avoir poussé à y aller, et que je réalise [pas compris] #00:31:34-0# je croyais que vous, que c’était, c’était comme ça. Vous aviez dit oui. Je le dis pas comme ça mais- et puis que y a plusieurs personnes qui disaient qu’ils étaient très contents d’avoir eu ça et puis d’avoir, voilà. Voilà d’avoir pu franchir en fait, ce, ce pas-là, ça c’est, ça c’est chouette. C’est, c’est là vraiment qu’on se rend compte que c’est, que c’est utile et que c’est un chouette travail qui a été fait. #00:31:59-1#

MH3: Hum hum. Ça prend un petit- ouais le travail. Ça reprend l’approche que t’avais par rapport à sa manière à elle d’approcher aussi les patients. Je pense que c’est pas interchangeable [rires d’MH4]. C’est pas tout psychiatre peut se trouver dans sa situation [acquiescement de MF3: “hum hum”]. J’ai envie d’évoquer ses compétences et puis peut-être son approche aussi, voilà. J’imagine que chaque région, ou que chaque population a sa manière de fonctionner et puis euh, peut-être ce qui est ici effectif, le sera peut-être pas à Neuchâtel de la même manière [acquiescement de MH1: “hum hum”], certainement que y

va y avoir des différences entre le haut et le bas et, et ce sera pas identique à Genève, à Lausanne ou à Berne, ou voilà, ouais. #00:32:48-0#

MH4: Mais- #00:32:50-9#

MH3: Une compétence [pas bien compris] c'est- de ce fondre un peu aussi dans, dans l'approche et puis ça c'est gage de, de réussite quoi. C'est clair. C'est clair. #00:32:59-0# Du point de vue, voilà, histoire de culture [acquiescement de MH1: "hum"], une manière culturelle de la médecine, de pratiquer la médecine peut-être. #00:33:08-8#

MH4: Pis- pis, il y a tout le côté, moi, pour moi, la vision du psychiatre, c'est, c'est de, d'amener la personne à se- à une meilleure prise en charge ou en tout cas avoir une, une ouverture et puis à pouvoir- à trouver des moyens pour avancer. Alors ça dépend dans, dans quel, quel genre de pathologie, c'est vrai. Mais, mais c'est là où son approche, moi je trouve a été très chouette. Parce que, parce que- chouette et efficace effectivement. [acquiescement de MH3: "hum hum"] Dans, dans la, on voit, il se passe quelque chose. La personne évolue dans son- voilà. Dans, dans- et trouve des moyens pour en aller, pour aller, pour dépasser son problème, ou en tout cas pour faire avec ou en tout cas pour aller de l'avant. #00:33:58-1# Les gens, moi j'ai entendu plusieurs fois les gens, ils ont pas peur mais on une grande hésitation de, de, d'aller chez le psychiatre et de parler, parler, parler pis après on se revoit deux semaines plus tard et on parle, on parle, on parle. Ils aimeraient le— l'échange [acquiescement de MF3: "ouais"] et c'est ça qu'ils apprécient entre autres aussi justement [acquiescement de MF3: "hum hum"]. #00:34:20-1#

MH3: Ouais.. #00:34:21-9#

MF3: Pis c'est un retour. Ouais, un retour assez direct je pense [acquiescement de MH3: "hum hum"], ouais. #00:34:27-3#

MH3: Ouais c'est ça, je pense pas qu'une approche psychanalytique soit- soit… Forcément facilement acceptée [acquiescement de MF3: "hum hum"] a ce premier contact quoi. #00:34:39-1#

MF3: Alors c'est vrai que ça peut être une problématique ben on peut [pas compris] ben ça on va voir. Bah ces changements de psychiatres qu'on va avoir, on va les vivre tout bientôt. [acquiescement de MH3: "ouais"] Mais, je pense que ça ça va être peut-être une des limites de- enfin on verra. En tout cas un des problèmes de ce projet. #00:34:55-5# [acquiescement de MH1: "hum"] C'est comment, comment vont se passer ces rocades… #00:34:58-6#

MH3: Si on change. Ouais. #00:34:59-8#

MF3: Ben oui. Il y a quand même des gens qui ont livrés beaucoup de choses et autres, on leur a proposé pis, "ah ben tiens on change". Je sais pas comment ça va se passer d'ici quelques mois, comment les gens vont vivre ces changements de médecins. [acquiescement de MH1: "hum"] #00:35:13-8#

MH4: En même temps, en même temps, j'ai l'im- mais peut-être c'est moi qui ait ça en tête, mais j'ai l'impression qu'elle, elle les oriente. Soit elle termine des- voilà. Elle, elle permet le, le passage qu'elle a fait avec le partage ou le projet qu'elle avait avec le, le patient soit elle les réoriente déjà maintenant #00:35:34-3# sur la- [pas bien compris] c'est une porte ou bien il me semble qu'il y a un ou deux patients…

MF3: -On va [parle en même temps donc pas bien compris] prendre que des- que des nouveaux patients ? #00:35:38-5#

MH4: Huuuuum. Peut-être pas "que" mais… Mais, mais beaucoup. #00:35:43-6#

MF3: Ok. #00:35:43-6#

MH4: Ça, ça, c'est ce qui est ressorti un peu de son rapport. Si j'étais, là j'étais, j'étais étonné mais, mais- voilà. C'est, c'est de toute façon c'était la vision aussi qu'on avait pis que, que j'ai encore toujours. Il y a pas de, de, de traitements ou long court avec elle à part quelques exceptions. [acquiescement de MF3: "hum hum"] #00:36:02-2# Et ceux-là [acquiescement de MH3: "hum hum"] elle essaye de les, de les réorienter [acquiescement de MH3: "hum"] si j'ai, si j'ai bien compris. Mais après- voilà. Il y a les cas lourds, qui nécessiteront de toute façon qui, qui, [acquiescement de MF3: "hum hum"] t'arrives pas au bout [acquiescement de MF3: "ouais"], qui nécessiteront toujours un, un accompagnement. #00:36:15-0#

MH3: C'est se prendre quoi. Aussi. On peut se laisser prendre dans une consultation [acquiescement de MH1: "hum"]. De se retrouver dans une situation long court. #00:36:27-4# [petite pause]

MH1: Et concrètement, comment vous faites ? Comment vous appeler ? Comment vous envoyer chez le psychiatre ? Que le patient soit par téléphone, par mail, toquer à la porte ? Vous le voyez ensemble ? #00:36:38-1#

MH3: Ouais. Avant d'aborder ça, j'aimerai juste ajouter, c'est vrai que les gens quand ils viennent ici, ils viennent pour du long court. Ils ont choisi un médecin, un médecin traitant et, et, et ils l'ont choisi et ben ça peut changer mais ils, ils se projettent dans vingt, trente- ils y en a même qui choisissent des jeunes pour être sûr qui va assurer jusqu'à sa retraite. La, la pérennité jusqu'à la fin de sa vie. Donc, nous on est un petit peu représentatif de ça où on se porte un peu garant de cette relation [acquiescement de MH1: "hum"] qui devrait peut-être aussi pouvoir perdurer et je pense que, comme tu dis, ça va être très intéressant, parce qu'il va y a voir un choc quoi. Il devrait pas, mais ça pourrait y avoir un, un impact [acquiescement de MF3: "hum hum"] assez fort dans le changement, très fort. Ouais. Pardon. [acquiescement de MH1: "oui"] C'est le petit ajout que je voulais [acquiescement de MH1: "oui oui"] faire avant de… #00:37:24-5#

MH1: Mais la question de, de contact. De quelle manière vous… Vous contacter la psychiatre ? Est-ce que- comment vous, vous, comment vous référer le patient ? Par mail, par téléphone, par toquer à la porte ? Vous le voyer en simple [pas bien compris] ? Qu'est-ce qui ?… #00:37:43-5#

MF3: Moi j'essaye de la voir si j'ai l'occasion de la voir, avant de, avant qu'elle voit le patient. Euh… Bon moi j'ai la chance de, d'être le lundi matin avec elle à D.. Et puis en général elle dîne là, donc on dîne plus ou moins ensemble, donc #00:37:59-2#

MH4: Ah c'est chouette #00:38:01-4#

MF3: on arrive pas si on fait pas que, on fait pas que parler psychiatrie non plus. Mais euh, mais finalement, peut-être que moi j'ai un petit peu plus de, de temps avec elle aussi. Euh, pour ce genre d'échanges. Donc j'essaye de le faire avec une voix. Et puis sinon, par, par mail ou un petit courrier ou autres. #00:38:16-7# Plutôt qu'un téléphone je dirai. C'est rare que je l'ai appelé. [acquiescement de MH1: "hum"] #00:38:20-6#

MH4: Mais elle a prit son rendez-vous en soin. Si le patient sort de la consultation où il a dit "oui d'accord je, je prends rendez-vous" [acquiescement de MF3: "hum hum"], on- il y a un agenda avec nos assistantes et pis elle pré- elle prend le rendez-vous comme un rendez-vous chez nous en fait. [acquiescement de MF3: "ouais"] Alors ça été un petit peu compliqué parce que c'est pas le même système, ce- c'était compliqué au niveau ordinateur, au niveau informatique. Parce que c'était pas, c'est pas le même système. Mais ça c'était- bon voilà. C'est des détails. Moi j'avais apprécié au début, j'avais accès à ce, cet agenda. Donc on pouvait même directement nous, mettre un, mettre quelqu'un, voir quand elle était là et pis si elle avait une place et puis, et puis directement- voilà. C'était même moi qui avait donné le rendez-vous à la, à la personne pis finalement c'est, ça ça c'est, j'ai pas insisté parce que y a eu tellement de changements et de, de tout. Et puis après la plupart du temps j'ai aussi essayé d'envoyer un petit email et de temps en temps j'ai oublié et je l'ai, et je l'ai fait après mais on a jamais voulu [rires]. Non, non mais par un- je crois que c'était ça [pas bien compris] quand on pouvait pas la voir théoriquement pour lui, pour lui dire. Une, une ou deux fois je lui ai lancé un coup de fil le, le- avant qu'elle voit le patient, parce que j'avais pas eu le temps de, d'écrire et puis je lui proposais de m'appeler ou je l'ai appelé pour juste lui dire deux mots sur la, la personne et la raison de l'envoi. [acquiescement de MF3: "ouais" ; acquiescement de MH1: "hum"] #00:39:46-8# Et puis… [inaudible] #00:39:47-7#

MH3: Moi j'ai, j'ai [se racle la gorge] surtout euh, prit les rendez-vous en tout cas avec le patient, voilà mis, mis sur un agenda comme, comme tu l'as- comme ça. Ce qui casse déjà un peu les codes. Les codes des autres psychiatres [acquiescement de MF3: "hum hum"] installés qui demandent que le patient lui-même téléphone et, et prenne lui rendez-vous [acquiescement de MH1: "hum"] et, et fasse les démarches. Et puis, bah après pour la transmission, mea culpa, c'était un peu chaotique [rires]. C'était des fois des emails, des fois des, des transmissions orales directes ou bien un petit bout de page A4 avec un résumé médical. Enfin ça été très prêt [?], ça s'est toujours fait, pis, pis voilà après, ça fait très bien aussi euh… #00:40:37-4# Dans, ce qui compte c'est- après les questions peuvent être posées hein, on a deux bureaux de différences donc euh [acquiescement de MH1: "hum hum"], c'est vrai voilà, que le fait de passer du temps avec aussi à midi elle a été lundi à midi assez souvent ici aussi, donc euh. [acquiescement de MF3: "hum hum"] #00:40:55-7# [pause]

MF3: C'est différent. #00:40:57-9#

MH4: Ce qu- ce qu'on a jamais tellement discuté euh, [souffle] et pis ce qui, ce qui était- moi j'ai trouvé un peu dommage, c'est qu'elle n'avait, en soit elle avait pas accès au [acquiescement de MH3: "hum hum"] dossier médical, du patient. Mais [acquiescement de MH3: "ouais"] de nouveau, [acquiescement de MH3: "ouais"], plus pour des questions informatiques. Et je trouve ça dommage. Parce que ça aurait été, ça aurait facilité aussi pour elle qu'elle jet un [acquiescement de MH3: "hum hum"] coup d'oeil [acquiescement de MH3: "hum hum"]. C'est parce que c'est vraiment deux systèmes complètement différents. Nous on a notre euh, notre système ici euh, interne, sécurisé et puis elle, comme elle elle fait partie du, du centre psychiatrique neuchâtelois elle devait avoir, un autre système. Voilà, le système extérieur et puis- #00:41:34-0#

MH3: Mais est-ce qu'elle aurait pas pu prendre un ordi, un code et puis- #00:41:38-0#

MH4: On aurait peut-être pu insis- plus euh, je me demande si on lui a posé une fois la question et pis elle nous a dit "non, non mais que c'est bien comme ça". #00:41:45-8#

MH3: Ouais, moi j'ai plus ressenti un désir de… #00:41:47-7#

MF3: [intervention inaudible] #00:41:47-7#

MH4: Qu'elle avait pas absolument besoin d'entrer [acquiescement de MH3: "ouais"] dans, elle l'a dit hein une fois.,que dans, dans le, dans le dossier médical que nous on fait du, du patient [acquiescement de MH3: "hum hum"] dans ce sens-là. Mais de temps en temps je me sui- je me disais ça aurait été- voilà, ça lui faciliterait les choses, si elle peut lire en diagonal et puis se faire une petit idée. #00:42:07-3#

MF3: Après je sais que Chantal Simon l'a, a vu une ou deux fois les patients avec elle directement. [acquiescement de MH1: "hum"] Elles ont fait des consultations ensemble, ça je me rappelle. #00:42:14-3#

MH3: Ah ouais.. #00:42:16-6#

MH4: Ouais, c'est vrai. #00:42:16-6#

MH3: Juste. #00:42:17-2#

MF3: Moi j'ai essayé deux fois, mais au dernier moment les patients se sont retirés. Enfin… Voilà. Cette première entrée en matière en disant "mais je serai avec vous, on fait la consultation ensemble et autres". [acquiescement de MH3: "hum hum"] Et puis euh, soit le jour d'avant ou autres, ils ont appelé pour, pour annuler [acquiescement de MH3: "hum hum"], donc ça s'est jamais fait. #00:42:31-3#

MH4: Ah, oui. #00:42:34-0#

MF3: Je sais que Chantal l'a fait. #00:42:37-2# [pause] #00:42:37-3# Et puis une fois je l'ai fait, c'était cette jeune en urgence où vraiment là j'ai, j'ai toqué pis là on a fait la consultation ensemble. #00:42:43-9# Euh, enfin je l'avais vu un bon moment pis je, voilà je

lui ai demandé la permission, j'ai dis "mais euh voilà, il y a la psychiatre qui est là est-ce que vous permettez ?" Pis on a continué ensemble… #00:42:52-6# Ça c'était, ouais..

MH4: Mais c'est chouette d'avoir cette possibilité #00:42:55-5#
#00:42:55-4#

MH3: Ouais, ça c'est incroyable. #00:42:56-5#

MH4: Ouais. Ça c'était, ouais. [acquiescement de MF3: "hum"] #00:42:58-0#

MH1: Hum. #00:42:58-5#

[silence] #00:43:00-7#

MH1: Elle, qu'est-ce qu'elle fait comme retour ? #00:43:03-6#

MH3: Par mail ? Par mail, ouais. Par mail ou, ou oraux. Il y a souvent des échanges orales et puis effectivement, par mail. [acquiescement de MH1: "hum hum" ; acquiescement de MF3: "hum hum"] #00:43:15-9#

MH4: Ouais, elle a fait des chouettes petits… Petits résumés par mail. Très brefs mais voilà qui nous donne un petit cadre et puis, et puis souvent quand même quand on avait l'occasion de la voir, oralement [acquiescement de MF3: "hum hum"]. Très, elle, elle sait être très succincte et claire et, et voilà. #00:43:33-7#

MF3: Ouais. Ouais, vraiment enfin… [inaudible] #00:43:35-7#

MH4: Très, elle est, elle est très synth- sythétique, [acquiescement de MF3: "ouais"] mais, mais bien. [acquiescement de MF3: "hum hum"] #00:43:38-7# où on est, où on est au clair. #00:43:40-5# [petit silence] Elle a cette, cette capacité qu'on attend. [acquiescement de MH1: "hum"] #00:43:42-5#

MF3: Et c'est vrai que ça c'est, ben de nouveau, c'est un retour précieux, parce qu'on a, on a bah des fois des ébauches de diagnostic, l'idée de, ben pour nous, pour la suite, on a, on a jamais quand on envoie à un psychiatre euh… #00:43:56-7#

MH4: Ah, ça, ouais. #00:43:58-2#

MF3: Euh, installés [acquiescement d'MH4: "ouais"]. Ben finalement c'est nous qui les envoyons, ils font leur prise en charge et ils reviennent. Mais nous on reste euh. #00:44:04-2#

MH4: Ouais, là t'as- c'est bien que tu dises ça. #00:44:07-4#

MF3: Sans ça, sans pistes aussi. [acquiescement d'MH4: "ouais"] #00:44:09-2# Voilà, [pas compris] son approche [acquiescement de MH3: "hum hum"], son regard et puis peut-être une manière pour le futur de, d'aborder les patients un peu différemment. #00:44:15-6#

MH4: Ouais. #00:44:16-9# [silence] #00:44:18-0# Ouais, ouais et puis en sachant aussi, et puis en, on a été très, très libre moi je trouve dans, dans les aussi, dans la médication. Qui pour moi, perd un peu son importance auss- c'est-à-dire ça fait partie mais voilà. C'est arrivé qu'elle change où elle a dit "mais moi je, je préfère ça dans ça, est-ce que, est-ce que ça joue ?" Une fois elle a, elle m'a téléphoné pour me demander et puis sinon c'était clair que si c'était elle sur la partie psychiatrique, c'était elle qui- qui changeait si elle voulait changer ou bien qui, qui gardait la même médication ou qu'il le, la, la renforçait si c'était nécessaire. Même là. Mais ça c'est une chouette collaboration qui s'est, qui s'est bien faite. #00:44:56-5#

MF3: Ouais, quand même. #00:44:58-0#

MH3: On est a mi-chemin vraiment entre euh les psychiatres euh installés et puis, et puis le médecin généraliste quoi. [acquiescement de MF3: "hum hum"; [acquiescement d'MH4: "ouais"] Je trouvais, on se retrouve là dans une [acquiescement de MF3: "hum hum"], dans un mode de fonctionnement et une approche qui est, qui est atypique. Par rapport à ce qu'on a juste l'habitude, et [acquiescement de MF3: "hum hum"] et probablement ce que vous avez aussi l'habitude, ouais [pas bien compris]. #00:45:23-4#

MH1: Et dans cette [pas compris] est-ce que vous vous arrivez à parler, de vos… de [pas compris] démuni, dépassé, vos inquiétudes, vos hésitations [acquiescement de MH3: "ouais"]. Avec elle vous arrivez euh en parler de votre souffrance aussi [inaudible] #00:45:43-9#

MH3: Euh…Oui je sais qu'elle avait aussi euh- moi j'ai, j'y ai eu peu recours. Chantal un peu plus. Une sorte de supervision [acquiescement de MH1: "hum hum"], d'une, d'une forme de, d'échange euh. Mais oui. Oi, oui. Alors y avait de la disponibilité pour, pour, pour nous aussi quoi [acquiescement de MH1: "hum"] pour, pour partager. #00:46:02-8# [pause]

MH1: Hum hum. #00:46:09-3#

MH4: Moi peu. Mais ces, ces questions de nouveau- voilà. Ce que je disais [acquiescement de MH3: "ouais"] tout à l'heure parce qu'elle était- c'est, c'est pas un manque de disponibilité de sa part, j'étais, je suis ailleurs ce, [pas compris] quand elle est, quand elle est là le lundi [acquiescement de MH3: "hum hum"] mais les, je sais que les, les fois où j'étais ici les lundis, y a chaque fois eu un échange ou l'autre mais pour parler de mon côté démuni [rires] je crois qu'on a plutôt toujours discuté du côté démuni du patient. [rires] [acquiescement de MF3: "ouais"] C'était implicite. [acquiescement de MH3: "ouais mais je-"] Je sais pas, moi j'ai, j'ai une patiente qui m'a dit, qui m'a dit, je, je- elle était au bout au travail ça allait pas du tout, elle était, pis elle était profondément dépressive et je pense qu'elle était suicidaire elle m'a dit "Je, j'attend la mort". Et c'est une dame qui a cinquante-huit ans ou soixante ans, "je suis à la maison, j'ai toujours aidé tout le monde, j'ai toujours fait pleins de trucs et maintenant, j'ai plus rien et j'attend la mort". Et ça, ça m'a fait peur.

Donc ça, ça j'ai- voilà, elle a vu le cup [??] et puis, parce que c'était un vendredi et puis le lundi d'après elle a pu voir, elle a pu voir CL-Psy qui était, ce qui était une bonne chose [acquiescement de MH1: "hum"]. Alors que elle avait pas de plan particulier, elle avait pas, c'était voilà. Mais… Je- comme je l'avais, comme je l'a suivais ou je la connaissais de, des dernières fois que je la- voyez quelqu'un qui dit "Je suis là et puis j'attend la mort" c'est, ça m'a beaucoup inquiété. Ça je me souviens, ça j'ai partagé avec elle, que c'était [acquiescement de MH1: "hum"] ça mon inquiétude parce qu'en fait, elle avait pas, elle avait pas fait de menace de suicide mais en même temps euh. Ouf, je la sentais pas du tout [rire léger]. En disant, je, je- voilà. #00:47:53-3# [acquiescement de MH3: "hum hum"] La patiente a su m'exprimer quelque chose de, de très noir dans… Dans sa situation, dans sa vie, dans son, dans ces émotions vécues, pis ça, ça j'avais partagé avec euh. Donc là j'étais aussi un peu impliqué. #00:48:07-2#

MH3: Mais c'est un partage plus informel mais. [réaction d'MH4: "non"] Formellement euh, ouais. #00:48:12-1#

MH4: Oui, plus informel, oui. Juste. [acquiescement de MF3: "hum hum"] #00:48:14-2#

MH3: Informel, ouais. #00:48:15-5#

MH4: Juste. #00:48:16-0#

MH3: Ouais c'est aussi ce que, ce que j'ai vécu et pis… #00:48:19-3#

MH4: Mais pour ça une grande disponibilité [acquiescement de MH3: " ouais. hum hum" ; acquiescement de MF3: "hum hum"]. Quand elle avait la porte, quand elle a la porte entre ouverte cette disponibilité alors, très très très appréciable. #00:48:25-9# [acquiescement de MF3: "hum hum"] Ou c'était plutôt moi de temps en temps qui disait "mais euh, voilà. Euh, t'as aussi ton horaire et puis moi aussi [acquiescement de MH3: "hum hum"] mais c'est vraiment vraiment chouette". #00:48:35-2# [acquiescement de MF3: "ouais"]

MH3: Elle s'est fondu dans notre fonctionnement de, de cabinet de groupe. [acquiescement de MF3: "hum hum"] #00:48:40-2#

[silence] #00:48:42-6#

MF3: Euh… C'est plutôt moi je pense- Ouais. #00:48:44-9#

MH3: Parce que c'est vrai qu'on échange aussi #00:48:45-8#

MF3: Je pense c'est plutôt quand on, présente des patients qu'on peut faire par une autre, une autre- [acquiescement de MH3: "ouais"] c'est le fait d'être démuni parce que ben, justement, la plupart du temps on les envoie parce qu'on est, on est démuni. Moi j'ai l'impression que c'est quand on arrive, enfin moi quand j'arrive à lui transmettre oralement pourquoi je lui envoie que là je, [acquiescement de MH1: "hum"] je peux plus partager [acquiescement de MH4: "oui"] ça. [acquiescement de MH3: "hum hum"] quand c'est par

mail, ce sera plus le résumé du, du patient [acquiescement de MH3: “hum hum”] seul en fait. #00:49:10-0# [silence] #00:49:13-3# [acquiescement de MH3: “ouais”]

chuchotements : “ouais c’est vrai, c’est juste”

MF3: Et puis après on avait fait une fois un entretien de manière assez formel alors là on s’était bloqué à un moment donné pour une patiente qu’elle a pas vu. Qui avait… Pas mal de médicaments et je voulais en retirer certains car c’était une période où elle allait bien. Et finalement pourquoi avec elle, comment faire un premier et autres. Euh, on avait pris un moment voilà de discuter de ce suivi-là. On avait bloqué une demi-heure [acquiescement de MH1: “hum hum”] où on avait vraiment fait ça un petit peu, sur, sur dossier. Mais sinon c’est vrai que c’est plutôt entre deux. #00:49:43-1# [silence]

MH1: Et cette collaboration, est-ce qu’elle modifie, votre pratique clinique ? Est-ce que vous avez l’impression que votre pratique clinique avec le patient est modifiée un bout ? #00:49:55-2#

MH4: Ça c’est difficile comme question. [silence] #00:49:57-5#

MH1: Votre regard finalement ? Votre- #00:50:01-6# Avec le psychiatre.

MH3: Mais la collaboration ponctuelle, ponctuelle d’échanges ou bien dans sa globalité ? #00:50:05-6#

MH1: Global. #00:50:08-8#

MH3: Global. #00:50:10-0#

[silence] #00:50:13-8#

MH4: Bon dans, dans très- généralement, pas avec les patients spécifiques avec, que, qu’elle a vu mais en général, c’est ça que vous voulez dire ? #00:50:18-8#

MH1: Les deux. #00:50:21-1#

MH4: Les deux. #00:50:22-2#

MH1: Oui. #00:50:22-7#

[silence] #00:50:25-3#

MH3: Je pense qu’elle, qu’elle peut l’influencer comme on l’a soulevé avant. La facilité, enfin voilà de, de, de transmettre rapidement au psychiatre une situation qu’on aurait gérer de manière différente si, si y avait pas eu cette disponibilité. Après euh, globalement, t’as raison, c’est difficile à répondre. Y- dans ces interactions y a forcément un enrichissement, donc forcément euh, un apprentissage, une expérience et, et quelque chose qui, qui, qui, qui améliore notre pratique, de manière absolue. #00:51:05-0#

MH1: Et ce serait traduit comment dans l'intervention alors [pas compris], si on prend la clinique? #00:51:08-8#

MH3: C'est pas par rapport à, à des questions de médicaments par exemple assez précise. Ou bien euh, une approche relationnelle d'une situation, mais qui pourrait se calquer sur d'autres situations [acquiescement de MH1: "hum"] #00:51:23-3# voilà, la, la [inaudibe] de- ouais, d'une situation ou d'un, d'un moment relationnel médecin-patient, on a pu discuter pis qui pourrait être changé [acquiescement de MH1: "hum"]. Ouais, j'pense. #00:51:38-2# Dans une- voilà. Dans un échange assez direct quoi quelque part. [acquiescement de MH1: "hum"] #00:51:42-0#

[silence] #00:51:44-9#

MF3: Je pense ça nous permet plein d'échanges de manière générale, qui, qui change probablement notre, notre regard pis qui font comme des espèces de petites formations qu'on, qu'on fait [acquiescement de MH3: "ouais"] au fur et à mesure. Après j'en ai pas forcément un exemple comme ça concret. De quelque chose que j'aurai modifié dans ma consultation, mais euh, l'impression que c'est- oui, un apport qu'on peut nous emmagasiner, au fil, au fil du, du temps probablement. [acquiescement de MH1: "hum"] Mais c'est peut-être encore un peu trop court pour euh, [acquiescement de MH3 : "hum hum, hum hum"] #00:52:16-9#

MH4: Peut-être. #00:52:19-0#

MF3: Pour avoir euh… #00:52:20-3#

MH4: Moi j'ai un, une ou deux personnes où, où je me suis après, beaucoup plus concentré sur le, le côté physique. Et sur, sur, des, des problèmes de, de dos ou d'articulations ou comme ça. Et je sais que [rire léger], que en, en sentant libéré quelque part. Libéré entre guillemets, mais parce que voilà la personne était contente de la prise en charge, et y avait cette [inaudible] #00:52:41-5# et je m'en suis, je m'en suis un peu moins occupé du côté, du côté psychologique et plus du côté, du côté somatique aussi. [acquiescement de MH1: "hum"] #00:52:50-5# Euh, ce que les gens apprécient à part ça de toute façon [acquiescement de MH1: "hum"]. Parfois on quand on part longtemps avec, disons quand y a une bonne partie de la consultation, qui est, qui est en discussion, l'examen manque ou, ou est un peu à court et puis, et puis les gens, les gens, je crois, apprécient, dans- en majorité, d'être examiné. #00:53:12-3#

MF3: Bon, peut-être que ce que j'ai changé c'est qu'elle m'a permis de, ben d'accepter du fait que certains patients avaient besoin de temps et que j'étais là aussi [acquiescement de MH1: "hum hum"]. Alors pas en tant que psychiatre mais finalement ils restaient chez moi, et euh, que finalement j'ai des gens que je dois voir toutes les semaines, ou toutes les deux semaines pendant ce, un certain temps et autres. Des fois je me disais "mais, j'arrive pas à les lâcher", enfin voilà. Ou, ou je me remettais moi en question dans ce suivi en disant "mais maintenant, euh, il faut- je peux pas continuer à les suivre comme ça sur, sur le long terme et autres". Pis que finalement c'est aussi une partie de leur fonctionnement et que chez moi-

alors après faut les trouver dans voilà, les caser [pas sur, pas bien compris] #00:53:53-2# dans un agenda et autres mais que euh, ça fait vraiment partie de cette prise en charge pis que on a le droit nous aussi de, ben de, voilà de prendre plus ce temps-là que je mettais avant en question [pas bien compris] #00:54:05-9# Je m'exprime peut-être pas très bien. #00:54:07-4#

MH4: Si, si. #00:54:09-1#

MF3: Que… #00:54:10-5#

MH4: Si, si. #00:54:10-7#

MF3: Que oui c'était normal de les revoir, de les revoir, de les revoir. Pis que ça faisait partie du traitement finalement. Simplement le fait d'être là des fois je lui disais [acquiescement de MH3: "hum"] j-j-… "En fait, ces consultations elles sont tellement importantes pis j'ai l'impression que la patiente ben voilà elle attend cette prochaine consultation et autres mais moi contrairement je fais rien. #00:54:27-2# J'ai l'impression de rien faire". Ben non. On est là, on suit, on est juste présents, et ça fait partie de la prise en charge. Et ça je pense que du coup je le- voilà. Je suis moins tout le temps de me dire "non mais allez on passe à trois semaines, on fait un petit peu [rires] plus, on espace un petit peu plus". Peut-être c'est ça que j'aurai changé. #00:54:45-7#

[silence] #00:54:49-6#

MH1: Et votre vécu, si on revient un peu sur votre vécu. Dans des situations plus complexes que vous avez décrit, est-ce qu'il- est-ce qu'il le modifiait avec la présence de, du psychiatre dans le cabinet ? #00:55:01-2#

[silence] #00:55:08-0#

MF3: Par rapport au fait si on avait, si ils étaient pas allé ou ? #00:55:11-3#

MH1: Face à une situation complexe, quand vous êtes, quand vous discuter avec le doctoresse ou quand vous envoyer le patient à la doctoresse, est-ce que votre vécu face au patient, est-ce qu'il- vous avez l'impression qu'il est modifié ? #00:55:24-5#

[silence] #00:55:27-3#

MH3: … Finalement et pis émotionnellement c'est clair que, c'est une porte de sortie quoi. #00:55:32-8#

MH1: De quoi ? #00:55:37-4#

MH3: Dans le sens que, dans ces vécus difficiles, ou ce sentiment de, de prise d'otage ou en tout cas de, voilà de, de, d'impasse que, que j'ai évoqué avant et bah euh, si on a accès à un con-frère, une con-soeur qui puisse effectivement quoi. #00:55:54-9# Et, et je crois que y a pas autant d'émotions, autant de, de poids relationnels que, quand on est dans ce domaine-

là. Euh, parce que quelqu'un qui est en train de faire un infarctus, effectivement, il faut qu'on puisse le transmettre quelque part. #00:56:11-6# Mais les structures sont pas en place, on appelle un [??], on appelle- voilà quelqu'un qui fait une crise d'épilepsie, de nouveau on se retrouve face à des, alors que ces situations qu'on sent vraiment limites, euh, bah elles sont de la même envergure qu'une pathologie somatique. Mais euh, y a moins le sent- voilà, il y a plus de sentiment d'emprisonnement et puis le fait d'avoir quelqu'un [rires] à proximité c'est quand même assez, ça, ça nous soulage un peu dans ces émotions quotidiennes. #00:56:43-4#

MH4: C'est ça. C'est aussi le mot qui me vient [intervention de MF3 inaudible] c'est un grand soulagement [acquiescement de MH3: "ouais"] de le savoir. Et, et je me rends compte maintenant où je sais qu'elle, qu'elle va plus être là et pis que y a pas encore de, de successeur dans certains, ça- dans certaines situations, ben voilà, on, on, on a plus cette porte, cette porte qui est, qui est là, qui, qui est pour moi plus une porte de facilité#00:57:07-7# Mais c'est [acquiescement de MH3: "oui"], c'est vraiment, c'est vraiment une porte de soulagement. [acquiescement de MH1: "hum"] #00:57:10-7# De, de savoir- et même si parfois, j'ai-je me suis dis "non c'est pas encore le- je vais pas faire appel à, à CL-Psy, je, on, on [pas compris] mais je sais qu'elle est là. Je sais que si ya, la, que si la situation devient moins, ou si soit moi j'arrive moins à faire avec cette situation ou, que la situation pour la patiente devient plus intolérable ou plus difficile si je pense [acquiescement de MH3: "hum hum"] à quelqu'un aussi avec des, des douleurs et des, des- qui revient régulièrement et ça brûle de plus de plus et tout. De savoir qu'elle est là, ça, c'est, c'est un grand, grand plus. Et qu'elle est, qu'elle est là, qu'elle est accessible. Et que, si, et que, y a une bonne chance que ça- voilà, que ça aille mieux après pour la patiente [rires]. C'est tout, tout, tout ça ensemble. Qui, qui, n'est pas donné, si je… lui dis, "prenez le téléphone et puis voilà, je vous donne quelques noms ou bien regardez dans le bottin téléphonique et puis vous verrez si ça vous va et puis…" Non. Dans deux mois, voilà. C'est tellement différencié, c'est, c'est un autre monde quoi. C'est une autre façon de, d'aborder, mais qui est, qui est très, très, très- c'est plus qu'agréable, c'est- ouais. #00:58:18-1#

MF3: Donc on a l'impression de les- ben d'arriver à continuer à les s-suivre [acquiescement d'MH4: "oui"] Ou à les- [acquiescement d'MH4: "c'est juste"]. Qui sont toujours [acquiescement d'MH4: " ce que tu disais"] dans des bulles de protection hein [acquiescement de MH3: "hum hum"] un peu, dans le sens que… #00:58:33-8# En fait quand on les envoie chez le psychiatre, ben voilà ils sortent de, de notre réseau, on les voit plus et autres. Et pis des fois euh, on les, ben on les récupère #00:58:45-2#, on fait [pas compris] ou ça a pas fonctionné le temps a passé, et autres. Tandis que là, ben voilà, ils font, ils font encore partis du cabinet et on- voilà. #00:58:52-7#

MH3: Dans l'espace. #00:58:55-1#

MF3: Enfin moi j'ai l'impression qui- voilà. Qui restent dans- #00:58:56-2#

MH3: Dans l'espace de confiance. #00:58:58-3#

MF3: Oui. #00:58:59-6#

MH3: Dans cet espèce de- #00:59:00-7#

MF3: Non mais je veux dire, si, si y vont mal, si, enfin je sais pas. [acquiescement de MH3: "hum hum"] #00:59:03-7# Ben que moi j'aurai quand même un retour ou une manière d'agir ou autres [acquiescement de MH3: "hum hum"]. C'est que sinon, on, on les perd, on les perd. #00:59:13-8#

MH4: Mais c'est dans- #00:59:15-2#

MF3: C'est trop, contrôlant ce que je dis. Mais- #00:59:17-7#

MH4: Nan, nan, mais c'est dans cette vision d'accompagnement [acquiescement de MF3: "ouais"] du patient qu'on est, qu'on est comme, comme généraliste euh, justement et qu'on sait, on est, des, des pas spécifiquement des thérapeutes, des soignants, mais vraiment des accompagnements- accompagnants. Et dans cet accompagnement, c'est, c'est de ça que tu parles [acquiescement de MF3: "hum hum"]. #00:59:35-2# C'est, c'est vrai, c'est chouette. Y-y-y-voilà ça, ça #00:59:39-5#

MF3: On avance quand même. [pas bien compris] #00:59:39-8#

MH4: c'est vraiment une, une, une aide très très appréciable. #00:59:43-2#

MH1: Hum. #00:59:46-7# Et par rapport à l'offre, on revient un peu au début de, de la discussion qu'on avait avant, avant l'interaction. Par rapport à l'offre existante jusqu'à maintenant, jusqu'à avant qu'elle arrive. Finalement, quels sont les bénéfices d'avoir un psychiatre ici ? #01:00:04-7# Plus globalement, si on prend le bénéfice comme ça, on parle beaucoup de différents bénéfices, mais, par rapport à l'offre d'avant, qu'est-ce qu'il y a de plus ? #01:00:14-0#

[silence] #01:00:18-0#

MF3: Moi j'ai l'impression je- je reviens toujours à ces histoires de communication, mais qu'on communique [acquiescement de MH1: "hum hum"]. Moi j'ai quand même [acquiescement de MH3: "hum"] plusieurs patients, où, je suis pas toujours très- où finalement, au bout d'un, d'un certain temps et autres, j'ai réussi à les envoyer chez un psychiatre mais finalement ils reviennent toujours chez nous. Parce que le psychiatre ils le revoient dans deux semaines, dans un mois et autres. Pis finalement quand ils vont pas bien et pis qu'ils vont vraiment pas bien, c'est chez, [acquiescement de MH3: "hum"] c'est chez nous qui sont. Et puis, on sait pas où- ben voilà, où on en est [acquiescement de MH3: "hum hum"], ils savent pas les médicaments [acquiescement de MH3: "hum hum"] qui prend nous- moi j'ai l'impression souvent, des gens que je peux avoir envoyé plus loin, me retrouver coincé. Parce qu'avant je faisais, je faisais peut-être pas bien, mais j'avais cette marge de manœuvre, de, de changer un peu de médication quand ils allaient pas bien, bien et autres, de, de pouvoir agir. #01:01:05-8# Et finalement le fait de déléguer, ben on remet ça un petit peu à une autre personne. Et entre deux moi je vois les gens qui vont pas bien mais j'ose plus tellement changer les choses parce que y a le psychiatre entre deux qui, ben qui, qui gère ça. Et pleins de fois je me dis "mais finalement, ben, je vais pas dire que je

regrette de les avoir envoyé mais je me sens un petit peu pris dans ce milieu-là". Pis ça c'est quelque chose qu'on a pas. Alors peut-être qu'avec les autres j'prends pas assez mon téléphone, je sais pas euh, probablement, faut aussi que je remette ça en question. Mais c'est vrai que j'ai l'impression- voilà qu'il y a un suivi en fait. On arrive mieux à les- voilà. Gérer ces, ces entre-deux [acquiescement de MH3: "hum hum"]. Parce qu'elle elle pourra se mettre entre deux, j-j- enfin voilà. #01:01:46-5# [silence] #01:01:49-6# Mais c'est vrai que je trouve que souvent- mais c'est aussi valable pour un cardiologue hein. [acquiescement de MH3: "hum hum, hum hum"] euh, y a un cardiologue, on a un peu de peine des fois à changer beaucoup de choses parce qu'il vient d'aller chez le cardiologue qui a dit autrement donc c'est pas que spécifique sur euh- du psychiatre. mais c'est vrai que voilà on a ce retour et quand même souvent, y a quand même un vieux réflexe qui fait que ils vont pas bien, souvent ils viennent ici euh, plutôt que, que appeler le psychiatre. Ou peut-être que nous ben en tant que cabinet de groupe on a cette disponibilité qui fait qu'on, on arrive à les voir plus rapidement aussi parce qu'on les voit entre collègues et autres et puis, une urgence on arrivera à lui trouver une place #01:02:26-4# [acquiescement de MH1: "hum hum"]. Je sais pas. #01:02:26-7#

MH3: C'est pas toujours- #01:02:30-1#

MF3: Mais souvent je trouve que c'est compliqué [acquiescement de MH3: "ouais"] quand même [acquiescement de MH3: "hum hum"]. #01:02:32-0#

MH4: Donc, il y a vraiment cette, cette barrière en moins et tout à l'heure on parlait de la barrière pour le patient, mais finalement c'est aussi pour nous cette euh, disons, c-c-cette barrière vers la, vers la psychiatrie. Moi, moi je n'en ai pas en soit, mais, mais la, la mienne, elle est- c'est pas la même que celle du patient, c'est, c'est, c'est une, une barrière d'organisation et d'aller et de retour, comme, comme tu le dis bien, dans, dans la communication, qu'on, qu'on a pas et qui tombe avec la, la présence de- voilà. D'une, d'une, de quelqu'un dans l- qui vient dans le cabinet qui est présent, qui est là, qui nous connait, que nous on connait, avec la, on a, on a un échange, c'est vraiment, vraiment ça un des grand grand points sur le plan de la, de la- pour moi sur le plan de la psycho-thérapie de la prise en charge j'ai appris une ou deux choses dans, dans ce qu'elle fait mais finalement ça serait si, si elle restait plus longtemps ça serait un des points que, que je serai intéressé à, à, à mieux- ouais à mieux comprendre aussi avec certains, avec certains patients. Et peut-être que ça vient justement du fait que toi, toi, toi t'étais, tu pouvais plus en parler avec elle [acquiescement de MH3: "hum hum"] #01:03:40-6# Moi j'ai été moins, moins un peu moins présent ici. #01:03:46-0# Toi je sais pas comment tu, tu l'as vécu en étant- toi t'étais là normalement le lundi. #01:03:49-9#

MH3. J-j'étais là effectivement mais- [pause] #01:03:53-5# De nouveau pas, pas peut-être pas assez profité, ouais effectivement. Je, je garderai aussi cette impression que on pourrait optimiser cette partie-là où on peut plus apprendre. Plus apprendre, plus approfondir, plus- avec des situations bien précises quoi. Des, des vécus d'histoires bien précises. #01:04:13-8# [pause] J'étais en train de me demander avant comment vivait le, le psychiatre parce que nous on est contents de pouvoir se soulager, on est référé. Pis le psychiatre qui est tout seul dans son cabinet pis qui peut plus référer plus loin [rires] #01:04:29-3# ces situations parfois

cauchemardesques ou angoissantes. De, de risque, de [pas compris] je sais pas. #01:04:36-4#

MH1: Ouais c'est intéressant vous ouvrez aussi la question où on est, des choses qui vous ont manqué un peu, plus de formation, par rapport aux psychothérapies ou que vous avez pu profiter, ça ça manquait un peu. Est-ce qu'il y a d'autres choses plutôt négatives que vous direz euh, ou moins positifs de, du projet ? #01:05:01-4#

MH3: Parce qu'on a été jusqu'à maintenant très positifs [rires d'MH4] #01:05:05-1# On est enthousiastes et positifs de nature [rires d'MH4, MF3 et MH3] c'est pour ça. [rires] #01:05:09-4#

MH1 [avec le sourire]: C'est pour ça que je pose la question. #01:05:12-7#

MH3: Ouais, ouais. #01:05:14-3# Ouais, je suis, je me suis souvent demandé qu'est-ce qu'il y a avait de négatifs, qu'est-ce qu'il y avait de qui- qu'est-ce qui pourrait être amélioré. Euh… Bon une semaine c'est long. Peut-être, ouais. Une semaine c'est long. Ça fait un [acquiescement de MF3: "hum hum"] long intervalle entre les, les deux. Euh. #01:05:33-7# Mais voilà, quoi. Ça peut pas être tous les jours, ou deux demi-journées purement pratiquement, pratico-pratique. Deux demi-journées reparties sur la semaine, je sais pas. #01:05:40-7#

MF3: Hum, hum. #01:05:45-9# Non, je dois réfléchir. Euh… #01:05:51-7#

MH4: Non, moi non plus. Dans, dans le cadre du, du projet, non. [acquiescement de MH3: "hum hum"] Le frein, le frein de l'informatique et d'avoir deux systèmes différents où elle était pas pleinement incorporée dans le- [acquiescement de MH3: "hum hum"] voilà, dans le dossier. Moi j'ai trouvé agréable que- alors je sais pas comment ça s'est passé avec un- on a un collègue qui n'utilise pas l'ordinateur qui a encore des dossiers papier et je, je sais pas exactement comment lui, il est, il est absent. Mais comment lui a fait, comme il a eu des- voilà. Comment il faisait pour, pour lui transférer, pour avoir un transfert d'informations si elle avait, ses, ses dossiers. Je- je pense pas qu'elle a eu des dossiers en main mais- #01:06:31-4# euh… Ça, mais ça c'est un détail. Bon c'est un détail technique qui- puis elle, elle a été embêtée aussi plusieurs fois, on pouvait pas faire appel à notre informaticien, elle a du, elle a du- ouais #01:06:42-1#. C'était compliqué [acquiescement de MH1: "hum"] pour elle aussi, mais elle a, elle a su faire avec et puis, voilà. #01:06:46-0# Mais au point de vue, au point de vue projet même et comme ça, comme ça s'est déroulé, moi j'ai, j'ai de la peine à- [acquiescement de MF3: "ouais"] toi, c'est comme tu dis. #01:06:57-1# On est des optimistes probablement, mais j'ai, vraiment- #01:06:59-3#

MF3: Moi je pense c'est juste- Ces, ces changement de médecins, [acquiescement de MH1: "hum"] de savoir, voilà, combien de temps ils vont rester. Alors là on a eu la chance d'avoir un peu plus d'une année est-ce que des fois on va avoir des rocades rapides, enfin voilà, moi c'est un peu l'interrogation que j'aurai pour euh, pour l'avenir si on a quelqu'un, ben je pense qu'il faut quand même, ben voilà, avoir [acquiescement de MH3: "hum hum"] pour s'investir dans le projet, comprendre notre fonctionnement et autres [acquiescement

d’MH4: “ouais”]. Donc c’est vrai que si, voilà on a des gens qui viennent pour six mois et autres est-ce que ça vaut la peine ? Enfin ce- comment va se passer ce tournus ? #01:07:26-0# [acquiescement d’MH4: “Et oui, oui”] Comment sur, sur le long court en fait. [acquiescement de MH3: “ouais”] #01:07:29-5#

MH4: Moi j’étais peu conscient que ça serait, euh, euh en fait ça se serait dans un, dans un an et demi en fait. C’est vrai. Maintenant que, maintenant que tu le dis. Pour moi c’était clair que c’était sur le long terme. [acquiescement de MF3: “ouais”] Et on était un peu surpris d’apprendre qu’elle, qu’elle- voilà, elle allait être forcée de, de, d’arrêter. C’est dans, dans ça c’est dans le cadre de son planning à elle et puis ça, ça- voilà. #01:07:53-1#

MH1: Et plus globalement pour le projet #01:07:54-4#

MH4: Sa, sa formation. #01:07:54-9#

MH1: Est-ce qu’il y a des, possibilité d’amélioration, de développement, qu’est-ce que vous auriez comme, comme idées ? [silence] De votre point de vue? #01:08:04-7#

MH4: Ah moi je- c’est très clair. Si de temps en temps avec un psychiatre, comme ça, qui nous connait, avec qui on est, on peut faire un groupe à limite [?], comme ça, moi je trouverai génial, dans le cadre d’un, d’un cabinet. Ça c’est, ça c’est pas- voilà. Non, avec nous, mais, mais. Peut-être un peu espacé, mais, mais avoir la possibilité, une fois quand même de, de se rencontrer, et puis de se connaitre aussi autrement et puis d’avoir- voilà. Là de bénéficier effectivement aussi du, du côté du, du côté du psychiatre et puis de l’échange. Alors on a des échanges sur des cas [acquiescement de MH3: “hum hum”] sur ceci [acquiescement de MH3: “hum hum”], sur cela, mais, mais moi je trouverai ça très très chouette, dans un- euh… Dans ce cas-là. #01:08:46-0#

MH3: Il existe hein des groupes [pas compris] #01:08:47-3#

MH4: Oui, oui, il y en a, y en a. #01:08:49-0#

MH3: Il existe. Mais c’est vrai, beaucoup plus- hétérogène, plus, plus grand. Peut-être ouais, c’est intéressant. C’est très intéressant, ouais. #01:08:59-|0#

[silence] #01:09:04-2#

MF3: Je sais pas, je sais que quand on avait discuté, ben y avait quand même discussion justement par rapport à la temporalité ou autres [acquiescement de MH1: “hum hum”] de venir deux fois par semaine et autres. Nous on était quand même un petit peu resté au fait que, on voyait pas forcément euh le besoin, l’utilité qu’ils viennent deux fois par semaine [acquiescement de MH3: “hum hum”], malgré tout. Alors c’est vrai que j’vois que ses agendas sont bien pleins et autres, mais en même temps, euh, c’est un fonctionnement qui est- [silence] #01:09:28-2# Ben j’pense qu’on reste un petit peu avec cette crainte de, ouais, de se poser la question si on envoie trop ou on enverrait trop ou autres. Pis ça ben, je pense qu’on a besoin d’un petit peu de temps mais finalement d’ouvrir une deuxième plage ça parait pas forcément nécessaire ou, [pas compris] donc je pense qu’on en resterait là, enfin,

rediscuter ensemble mais [acquiescement de MH3: “hum hum”]- Par rapport aux deux autres cabinets qui étaient plutôt il me semble à dire “on aurait besoin de plus” [acquiescement de MH1: “hum”]. Finalement ben nous ça nous laisse une porte de sortie, mais- qui, voilà- Qui semble bien fonctionner comme ça actuellement. #01:10:06-3#

MH4: Ouais, c’est vrai. #01:10:09-5#

MH1: Ah, et dans la pratique de la collaboration, est-ce qu’il y aurait des choses à développer ? À proposer ? Pour améliorer la collaboration? #01:10:19-4#

[silence] #01:10:21-7#

MF3: Alors suivant la personne, peut-être. D’avoir un moment euh… Plus fixe d’échanges et autres. Parce que là c’est fait entre deux portes, qu’elle laisse grandes ouvertes ou à midi ou autres [acquiescement de MH1: “hum”]. Suivant qui vient [acquiescement de MH3: “hum hum”], qu’a pas cette ouverture-là. On peut avoir relativement peu d’échanges donc à voir euh- je sais pas dans quelle manière hein mais euh, euh, un peu plus de plage d’ouverture pour euh, [acquiescement de MH1: “hum”], pour discuter entre nous finalement [acquiescement de MH1: “hum”] #01:10:46-9#

[silence] #01:10:51-0#

MH4: Oui, oui. Moi- moi je vois ça si, si c’était sur- réparti sur deux jours on aurait peut-être plus l’occasion d’avoir un- enfin quelque chose comme une supervision [acquiescement de MF3: “hum hum”] ou quelque chose- de, de vraiment un échange, la, la présence d’avoir une, une consultation à, à deux euh- avec elle ou ça sent. Au, au tout début, donc, quand on a parlé du projet on avait un peu peur [acquiescement de MH3: “hum hum”] on, on se disait “mais on aura pas, est-ce qu’on aura assez pour remplir euh [acquiescement de MH3: “hum hum”] une journée”. Euh- voilà. Pour elle, on savait, on savait pas [acquiescement de MH1: “hum hum”] vraiment [acquiescement de MH3: “hum hum”]. Et puis après on après on a pas du se forcer [rire léger]. On était très, très très contents. Là où je disais que j’étais content de moi de voir son- de pouvoir avoir accès à son agenda, c’est de voir en même temps de me, de réaliser à quel point il était plein ou pas plein. Ou, [acquiescement de MH1: “hum”] est-ce que sur deux semaines y avait de la encore, y avait encore de la place, est-ce que sur quatre semaines y avait plus de place, ce qui a, ce qui a jamais été, jamais été le cas. Mais, mais ça me permettait aussi de savoir quelle est la, la charge de travail et les, la disponibilité #01:11:55-1# [acquiescement de MH1: “hum”]. #01:11:55-8# Euh… [pause] Voilà. #01:12:03-0# [pause] Oui. [acquiescement de MH3: “hum”] #01:12:04-3#

MF3: Ouais de profiter un peu plus de sa présence je pense. #01:12:06-3#

MH4: Oui. Tout à fait. #01:12:06-6#

MF3: Parce que là on en fait quand même beaucoup profiter les patients [acquiescement de MH1: “hum” ; acquiescement de MH3: “hum hum”]. Nous un- de manière indirecte de toute façon [acquiescement de MH3: “hum”], mais peut-être qu’on pourrait plus en profiter. #01:12:14-9#

MH4: Moi je pense que oui. #01:12:17-5# Oui, oui.

MF3: C'est vrai que, ouais. #01:12:19-0#

MH4: Même au niveau de ce qu'on fait nous même, au point de vue, au point de vue utilisation de médicaments et puis au point de vue de la, effectivement, de la, de la psycho-thérapie elle-même aussi. [acquiescement de MF3: "hum hum"]   Qu'est-ce qui fait que… Je revois la patiente un mois plus tard et piselle a le sourire [rires] #01:12:30-9# Qu'est-ce qui a changé ? [rires] #01:12:33-4#

MF3: Après c'est pas faire de la place que sur son agenda à elle.   #01:12:35-8#

MH4: Non, et c'est pas non seulement pour faire sourire les patients non plus mais— [rires] #01:12:39-7#

MF3: Non, non mais, voilà, Non mais… #01:12:41-3#

MH4 [en rigolant]: Tout à fait #01:12:41-6#

[long silence] #01:12:43-6# #01:12:51-6#

MH1: Est-ce qu'il y aurait d'autres- d'autres besoin ou, pour vous, plus. Vous avez parlé de [pas compris], vous avez parlé de supervision. C'est vrai vous avez parlé plus de votre besoin de vous, finalement, de la présence du psy. Est-ce que vous avez l'impression que les besoins du patient est plutôt, bien complet. Mais vous, vous auriez d'autres besoin concrètement. [pause] #01:13:14-3# Euh, médication, psycho-thérapie, formation plutôt, c'est ça que vous dites ? #01:13:20-2#

MH4: Oui c'est ça. C'est de ça que je parlais là, là à la fin. Dans la, dans- en parlant, en parlant psychothérapie et puis, et puis médication. D'avoir une, oui, oui, une orien- une meilleure orientation, on, on reste quand même sur nos- voilà, sur ce qu'on a appris [acquiescement de MH3: "hum hum"] sur quelques substances qui se… Seraient nouvelles et qu'on apprend souvent à connaitre à travers des [acquiescement de MF3: "hum hum"], des réclames, des représentants, des- pas tellement à travers le monde psychiatrique non plus [acquiescement de MH1: "hum"]. Et là, là ça fait du bien si, si on a cette possibilité-là. Mais là, là le pro- ce, ce- j'ai l'impression que en, en, en parlant comme ça je, j'élargis, on élargis le projet. #01:14:00-1# [pause] Mais, voilà. #01:14:04-7#

MH3: Y a pas, de… Ouais. #01:14:04-7#

MH1: [pas compris] … C'est ça la question. #01:14:05-1#

MH4: Tout à fait, tout à fait. #01:14:05-8#

MH3: Ouais. #01:14:06-9#

MH4: Dans une, dans une collaboration avec un psychiatre disons où on, [acquiescement de MH3: “ouais”] où effectivement un psychiatre est présent euh, plus ou 50% de la, de la semaine, quelque chose comme ça. C’est, c’est ça serait de toute façon très utile. [acquiescement de MH1: “hum hum”] Ça j’en, ça j’en [acquiescement de MF3: “hum hum”] moi j’en suis convaincu parce que, à cause, à cause de la, l’importance que prend le… Cette partie dans, la psychiatrie dans notre euh [acquiescement de MH1: “hum”], notre consultation de tous les jours [acquiescement de MH3: “hum hum”]. #01:14:33-2#

MH3: Je suis pas un assidu des demi-journées de formations continue à Neuchâtel et à la Chaux-de-Fond. Mais ce qui a eu souvent des psychiatres qui… ? #01:14:43-7#

MF3: Non. Moi je vais régulièrement. #01:14:45-6#

MH3: Non ? Souvent des pneumologues, souvent des cardiologues [acquiescement de MF3: “hum hum”], y a des, des domaines différents donc c’est Neuchâtel, la Chaux-de-fond, vous avez peut-être eu l’occasion d’y participer, demi-journées organisées par la SNM et pis [acquiescement de MH1: “hum” ; acquiescement de MF3: “hum hum”], c’est vrai que y a pas beaucoup de, [acquiescement de MF3: “ouais”] ils parlent peu de…Psychiatrie quoi. #01:15:00-8#

MF3: Après c’est nous qui- [pause] mettons les sujets [réaction d’MH4 inaudible] dont on veut [acquiescement de MH3: “ouais”] qu’on parle au prochain donc euh. #01:15:06-0#

MH3: Ah, ouais. #01:15:07-1#

[rires de MF3] #01:15:08-6#

MH4: Ouais. #01:15:08-9#

MH3: Donc euh… #01:15:09-4#

MF3: Je sais pas si c’est, voilà. Mais oui c’est que je [acquiescement de MH3: “ouais”] pense pas que y en a eu beaucoup, ouais. #01:15:15-2#

MH3: Je réfléchis [acquiescement de MF3 : “hum hum”] au congrès de médecine générale, ouais, c’est peu. [acquiescement de MF3 : “hum hum”] #01:15:20-5# [pause] Ouais. #01:15:23-7# [pause] Donc c’est un réel besoin. #01:15:25-3#

MH1: Hum. #01:15:26-7#

MF3: Autre chose- #01:15:28-4#

MH3: Donc une confrontation [acquiescement de MF3 : “ouais”] quotidienne, ou en tout cas très fréquente [acquiescement de MF3 : “ouais”]. En augmentation, ouais. #01:15:32-6#

MF3: Bah ça a rien à voir, mais je pense encore autre chose, où je trouve que y a un, un apport qui est bien intéressant que je commence à utiliser. C’est qu’elle commence aussi à

connaitre un petit peu le réseau ici, de Neuchâtel et autres. Et des fois on a des gens qu'on veut aiguiller [acquiescement de MH1: "hum"], mais c'est vrai que- ben en fait, on, voilà [pas bien compris] connaitre bien les psychiatres, enfin on a quand même des histoires de, ben justement, de différentes approches, personnalités et autres et là elle peut bien nous orienter. #01:15:53-9#

MH4: Ça c'est juste- #01:15:56-5#

MF3: En ayant un bref du patient euh, jeune, pas jeune, de la problématique et autres. Euh, et ça, j'ai, j'ai peu fait jusqu'à maintenant mais c'est vraiment quelque chose d'intéressant à utiliser. #01:16:05-8#

MH4: Hum hum, ça c'est juste c'est son #01:16:07-9#

MF3: Donner des noms aux psychiatres- #01:16:08-8#

MH3: Ouais c'est vrai, moi j'envois, euh le cardiologue, ben je choisis lui parce qu'il est plutôt comme ça [acquiescement de MF3: "ouais"] ou je choisis lui parce qu'il a plutôt cette, cette attitude #01:16:15-1#

MF3: Et elle, elle les connait #01:16:16-8#

MH3: Neurologue aussi, c'est la même chose. Pis pourquoi pas la psychiatre [acquiescement de MF3: "hum hum"] [pas compris] de ses aptitudes, de ses connaissances voilà. #01:16:23-2#

MH4: Et puis après aussi [acquiescement de MH3: "hum hum"] son, son lien avec le, le CPN fait qu'elle a aussi un lien avec les assistantes sociales, avec euh, voilà. Avec euh… Le, le lien social et qu'elle a une ou deux fois pu, aussi, qu'elle a adressé des, des personnes [acquiescement de MH3: "hum hum"] directement, pour une consultation avec une, une assistante sociale ou pour, pour essayer de régler des, des problèmes, voilà. Plutôt, plutôt sociale ou financière ou autres dans, dans ce sens-là et ça c'était, c'était excellent. Là c'est vraiment, on parle d'autres choses, mais ça c'était, c'était très très bien parce que ça, ça nous manque ici, clairement. #01:16:59-4#

MH3: Tout le réseaux psycho-social. #01:17:00-6#

MH4: Oui. [acquiescement de MH3: "hum"] Ce réseau, ce réseau psycho-social et même le [acquiescement de MH3: "hum"] même le savoir aussi quelque fois de, de voir quelles sont les possibilités, qu'est-ce que, comment, comment orienter [acquiescement de MH3: "hum hum"] orienter dans, dans situations aussi voilà- le travail avec les, avec des- des incapacités de travail, ce genre de, de problématiques. #01:17:22-7#

[silence] #01:17:26-2#

MH3: Hum, hum. Hum hum, ouais. #01:17:28-0#

MH1: Très bien. Est-ce qu'il y aurait d'autres choses que vous voulez ? On arrive doucement à la fin. Est-ce qu'il y aurait d'autres choses que… Vous pensez ou ? Par rapport à cette collaboration, par rapport à cette intervention, comme ça, est-ce que il y a des choses qu'on a oublié, qu'on a pas, qu'on a omis euh, à mettre le focus. Qui viendraient comme ça? #01:17:59-2#

MH4: Non je crois qu- si je me re-situe à, à comment c- disons à quoi en repenser avant que ça commence et où est maintenant, on reste vraiment très, très, très posi- disons positifs et [acquiescement de MH1: "hum"] et assez enthousiastes de, de ce projet [acquiescement de MH3: "hum hum"] parce que- et, et d'ailleurs l'autre cabinet de groupe de Cerny [?], dès qu'ils ont entendu que y avait euh, une psychiatre qui venait régulièrement, on dit [pas compris] et nous aussi. [rires] Donc euh, voilà, sans, sans demander beaucoup qu'est-ce qu'elle fait, qu'est-ce [acquiescement de MH3: "hum hum"] qu'elle fait pas mais, mais rien que cette idée de, de, de ramener la- un psychiatre, une, une psychiatre, un, un spécialiste dans, dans le cabinet est vraiment, vraiment bénéfique, voilà, dans nos relations. Dans nos relations avec les patients, dans notre suivi euh, [acquiescement de MH3: "hum hum"], sur le long terme. Pour nous et pour eux. [acquiescement de MH1: "hum hum"] Ça oui. #01:18:50-8#

MF3: Après on a pas encore beaucoup de retour des, des psychiatres eux-mêmes, je sais que c'était quand même une interrogation, de, de savoir la réaction des psychiatres à ce projet [acquiescement de MH3: "hum hum, hum hum"] quand euh, quand euh… Quand il a été mis sur pied. Euh, j'en ai jamais re-entendu parlé, de la part du, du docteur Sagnin [?] ou de, enfin je sais pas, en fait. #01:19:13-0#

MH4: Moi j'ai- j'ai… #01:19:14-8#

MF3: Elle est enfin, voilà, est-ce qu'elle a un retour quelconque euh, positif, négatif [acquiescement de MH3: "hum hum"], est-ce que… Malgré tout. On envoie de moins de- je sais pas. De patients. Non, mais on dit ben c'est vrai, on a une psychiatre à D., est-ce que de manière euh [pause] euh est-ce que y a des gens que je lui aurai envoyé à elle, que je- enfin, je sais pas. #01:19:39-2#

MH4: C'est juste. Surtout là on a pas [pause]. Actuellement, ouais. #01:19:43-7#

MH3: Y a un retour de nos assistantes. #01:19:44-6#

MH4: Hum, hum. #01:19:45-3#

MF3: Hum, hum. #01:19:46-4#

MH3: Enfin ça s'est bien passé pour elles aussi. [acquiescement de MF3: "ouais"] #01:19:48-1# Je sais pas, je leur ai [acquiescement de MF3: "je crois"] pas demandé un feedback [acquiescement de MF3: "ouais"], je pense que, elles se sont fait à l'organisation, à la présence de quelqu'un de nouveau, à l'orientation, l'accueil [acquiescement de MF3: "ouais"], ouais tout ça. Je sais pas. Vous avez un retour ? Un ? #01:20:02-4#

MF3: Disons que en bas elles euh [pause]. Enfin c'est plutôt ici qu'elle gère l'agenda et autres [acquiescement de MH3: "hum hum"] pis elle, elle demande pas grand chose [acquiescement de MH3: "hum hum"], donc à part juste accueillir les gens, sur le bas il y a pas beaucoup [inaudible] [acquiescement de MH3: "hum hum"]

MH4: C'est vrai ? Ah oui. #01:20:15-5#

MF3: De travail à priori, mais. #01:20:18-1#

MH4: Elle donnait pas de, de rendez-vous à D.. Pas tellement. #01:20:20-8#

MF3: Ça a toujours été compliqué, donc [acquiescement d'MH4: "ah oui"] en général, ils appellent ici, je, je pourrai pas te dire pourquoi, mais… #01:20:27-2#

MH4: Ouais, c'est ça le côté, le côté informatique a été un peu, et reste un peu compliqué, de la prise d'agenda et tout. #01:20:34-8#

MH3: Personnalité peut-être aussi quoi [rires] #01:20:37-4#

MF3: C'est vrai que ça c'est peut-être quelque chose qu'on pourrait, ouais- #01:20:37-6# Non, qu'on pourrait améliorer [acquiescement de MH1: "hum"]. D'arriver à savoir facilement quand c'est qu'elle voit les patients sinon on les voit entre deux ou comme ça. [pause] Mais bon. #01:20:47-6#

MH3: Hum. Hum, hum #01:20:52-2#

MF3: J'ai dis quelque chose de négatif comme ça. #01:20:57-5# [rires de MF3, MH4 et MH3] #01:20:57-4#

MH4: Non, mais moi j'ai pas autres chose qui me vient, qui me vient en tête comme ça. #01:21:03-4#

MH3: Non, moi non plus, là comme ça. #01:21:04-2#

MH1: Bon, très bien. Ben merci pour votre euh, travail. #01:21:13-9#

Focus Group 3

MH1: Très bien, merci. #00:01:23-8# [pause] Alors [pause]- moi je voulais ouvrir un peu la discussion autour de la question de- très pratique, quelles situations vous faites appel ? À leur proposer ? #00:01:33-5#

MH5: Moi je peux- les, les, les fois où j'ai fait appel, c'était surtout les questions où il y avait des, des problématiques chroniques avec des questions asécurologiques donc pour les questions d'arrêts de travail, prolongé [acquiescement de MF4: "c'est ça"], dépressions, burnout. Pour les questions de justifications, AI, etc. Les questions de médications qui devenaient plus lourdes- assez lourdes, donc des gens qui ont des traitement psychotropes assez lourds, addictions, des choses comme ça. Pis fallait qu'il y ait, quand même quelqu'un qui puisse aussi- enfin… Un avis un peu plus spécialisé. Et puis, pis après de temps en temps aussi pour les avis diagnostiques, justement pour les situations qui sont, qui sont pas très claires ou, pour les précisions vraiment du diagnostique psychiatrique. #00:02:24-4#

MH6: Oui, je rejoins MH5. Dans ça, effectivement dans, dans ces situations-là- euh… De l'avis diagnostique où des fois on a l'impression "ah ce patient-là normalement il devrait être suivi par un psychiatre". Et lui il met les pieds contre le mur. Et, et, et, et comme on a psychiatre ici qui connait les locaux, il connait où on est, pour lui, c'est euh, c'est plus facile de ve- de, de consulter ici. Et, et, et de, de… De venir. J'ai, j'ai deux ou trois situations comme ça où, où, où finalement les patients étaient d'accord de, de, de venir et puis- où où un début de travail a pu être commencé. #00:03:04-8#

[Pause] #00:03:08-0#

MH7: Moi j'ai des situations où il y - on a réussi [rires], dans la même matinée à passer de mon bureau- directement à, à la psychiatre. Donc, je lui disais "voilà, j'ai une situation qui nécessitera un suivi, il y a eu un burn-out". Une jeune aussi avec des troubles [bruit de sonnerie] #00:03:25-7# , troubles anxieux où, où, la psychiatre était pas prête et pis on a pu réaction de MF4: "C'est ce bruit qui [pas bien compris] je pense"] carrément euh- dans la même matinée avoir l'évaluation, [réaction de MF4: "S."] ça a suivi, ça c'est mis tout de suite en place. #00:03:36-0# [acquiescement de MH1: "hum"]. Donc y avait [réaction de MF4 inaudible] vraiment une continuité- directe quoi [acquiescement de MH1: "hum hum"]. Ça c'était euh… #00:03:40-1#

[pause] #00:03:42-7#

MF4: Et puis aussi la, la, la question du temps parce que les cas psychiatriques ils demandent une heure de consultation. Que nous comme généralistes on peut pas, la, la donner parce que on a d'autres choses à s'occuper. Et puis… Déjà de pas donner ce temps-là, le, il y aura cette sorte de, de, de, coupe de confiance parce que ils ont déjà mal et puis tout d'un coup si on dit qu'on a pas le temps non plus, ils se sentent complètement abandonné, ça aggrave ces problématiques qu'ils ont. Et puis ça c'est un- c'est franchement un aspect qui, qui me fait souvent adresser les gens chez le psychiatre [acquiescement de MH1: "hum"] #00:04:31-

2#. L’insécurologie qu’on sait maintenant parce que j’avais un cas directement que, son psychiatre il est allé pour six mois sabbatique dans un autre et puis les assureurs, ils faisaient des pressions d’avoir des rapports, j’ai fais les rapports dans l’état comment il était le patient, pis tout d’un coup ils m’ont dit carrément que “vous êtes pas psychiatre” de prononcer pour votre patiente. Voilà, carrément comment ça. Jusqu’à le psychiatre elle est venue, parce que elle voulait pa commencer pour six mois avec un autre psychiatre de, de, éplucher tout son passé. Et j’ai dis “ok, je suis d’accord vous suivre six mois”. Et puis quand j’ai eu des assureurs cette sorte de, de [pause] de léger de réponse ça m’a franchement touché hein [acquiescement de MH1: “hum”] #00:05:23-5#. Et puis, voilà le psychiatre quand il est venu, il a seulement [pause]- mis les histoires. Il a seulement euh, rajouté deux choses à sa manière. Et puis, c’était les mêmes, les mêmes chose que moi j’ai constaté qu’il a constaté, et puis voilà. Ça veut dire, ça reste quand même que le psychiatre il doit se prononcer dans le burn-out, dans les troubles d’adaptation, dans les dépressions qui nécessitent un arrêt de travail plus que- trois-six mois ou plus. [acquiescement de MH1, MH7, MH5 et MH6: “hum”] #00:05:57-2# [pause] Ouais. #00:05:58-9#

MH5: Il y a une ou deux fois peut-être pour compléter la chose, la chose où ça- c’était aussi un peu gestion d’une, d’une crise. Gestion un peu de l’urgence. [acquiescement d’MH7: “ouais”] Bon, c’est vrai que comme elle était là qu’une fois par semaine, fallait que ça tombe déjà assez au bon moment mais ça à permis une ou deux fois aussi de juste, un petit peu stratifier les situations qu’étaient un peu en zone grise. Pas urgence absolue mais, juste un petit peu, comment, comment s- comment on gère. Y a une ou deux fois où ça a pu être [acquiescement de MH1: “hum”] utile aussi [acquiescement de MH1: “hum”] #00:06:21-4#

MF4: Et puis l’accès, oui. #00:06:24-2#

MH6: Moi- Moi j’étais je trouvais surtout c’était l’accès facilité euh… #00:06:27-7# Euh, à, à quelqu’un qu’on peut croiser dans le couloir, qu’on peut euh, aussi de, de, de, de savoir que, que le délai il va pas être euh, long. Que, que, que moi ou mes assistantes peuvent presque aller fixer un rendez-vous dans l’agenda de, de, de, euh, de la, de la psychiatre c’est, c’est très- très appréciable parce que euh, ça euh, aide le patient il doit laisser un message sur le, le répondeur et attendre après que le psychiatre le rappel, j- je trouve [réaction de MH5: “oui pas”] #00:07:01-2# …ou pas #00:07:02-8#

MH7: Ils partaient avec un rendez-vous quoi [acquiescement de MH6: “ouais” ; acquiescement de MH1: “hum hum”] Suivant les cas. #00:07:05-0#

MH6: Oui, ils partent ave- ils partent avec un rendez-vous. [acquiescement de MH1: “hum”] #00:07:06-7#

MH7: Pis même à un moment nous on avait quand même [se racle la gorge]. Bon après c’est un peu plus compliqué pour y parvenir, mais on a accès à l’agenda. On peut quand même fixer dans le- [acquiescement de MH1: “hum”] dans le, dans le truc on regarde s’il y a des plages et puis on, on peut fixer le rendez-vous [acquiescement de MH1: “hum hum”]. Donc ça c’est on voit le patient et puis on dit “écoutez voilà je vous [pas compris]”. #00:07:22-0#

MH5: Ça je trouvais très bien au début quand on avait- [inaudible] l'agenda encore #00:07:24-3#

MH7: On pourrait faire encore. #00:07:25-4#

MH5: Ouais. #00:07:26-8#

MH7: On pourrait faire, sauf que y avait les codes d'accès. Quelque fois on se dit "bon demandez en bas [acquiescement de MH1: "hum"] elles vous donneront rendez-vous en bas quoi". Moi je faisais plus directement. [acquiescement de MH6: "ouais"] #00:07:33-3#

MF4: Et puis le, le, le feedback, les communications. Ça veut dire on a le psychiatre sur place, on lui donne euh… Des détails des situations et elle nous donne aussi. Euh. au niveau du traitement aussi, je pense qu'on a pas ça avec les autres psychiatres. On a jamais presque de rapport psychiatre, on doit téléphoner mille fois pour voir ce qu'il se passe hein. #00:07:54-3#

MH5: Bon là y avait le petit bémol, avec elle moi j'ai jamais reçu de rap- ou une ou deux fois un rapport mais c'est plutôt une copie d'une rapport pour l'AI ou des trucs comme ça [acquiescement de MF4: "ouais"]. #00:08:02-8# Par contre que c'est vrai qu'au moins on a un feedback quoi, c'est vrai que la plupart du temps- #00:08:06-1#

MF4: Feedback, on discute, oui. #00:08:07-2#

MH5: Chaque fois que y a des gens qui vont voir un psychologue ou un psychiatre nous on a- enfin moi j'ai jamais de rapport ou de contact [acquiescement de MF4: "non"] direct [acquiescement de MH1: "hum" ; acquiescement de MF4: "non"]. #00:08:11-8# Et puis d'ailleurs, ben si un jour le suivi s'intervient [???]. On sait plus du tout ce qui s'est passé [acquiescement de MH1: "hum"], pis y a plus rien qui- tout qui disparait. Tandis que là, c'est vrai qu'on a des feedback réguliers. #00:08:20-5#

MF4: Non, ça c'est bien parce que moi quelques cas qu'on gère ensemble. C'est bien. Mais aussi j'ai de la chance parce que, à la pause de midi, on est là hein. [acquiescement de MH1: "hum"] Et puis on discute. [acquiescement d'MH7: "hum hum"] #00:08:30-0# Euh… #00:08:32-5#

MH6: Toi, toi tu, tu es là. #00:08:33-0#

MF4: Moi je suis là. Euh, je suis franchement, on- c'est bien, on, on… On a plusieurs patients que- [pause] on les gère ensemble. C'est, c'est excellent. Ça, ça me donne aussi à moi de… Des idées, comment il faut réagir, et puis ça me donne plus des outils, être plus complète dans les cas difficiles [acquiescement de MH1: "hum"]. Qu'est-ce qu'on doit dire, qu'est-ce qu'on doit pas dire. #00:08:55-0#

MH1: Vous pensez à une situation précise ? À une… Situation emblématique comme ça qui pourrait dire "ah là c'est-" ? #00:09:01-6#

MF4: Ouais. Je sais pas après si on doit donner des noms ou prénoms. #00:09:04-8#

MH1: Non, ben non. #00:09:05-5#

MH7 (ou MH6): Non, ben non. #00:09:06-0#

MH1: Juste la situation. #00:09:07-3#

MF4: J'ai, j'ai une patiente euh… Qui a des problèmes d'obésité, avec pas mal de problèmes somatiques associés au niveau de, de arthroses, des [pas compris] liés à son problème du travail. Et puis… #00:09:22-4# Sa obésité dans le cas des crises de boulimie qu'elle arrive difficilement à gérer [acquiescement de MH1: "hum"] euh, candidat de stress plus particulière [pas bien compris]. Et puis… Elle a pas mal des arrêts de travail, du à ce mal-être mais qu'elle a caché longtemps, qu'elle vivait dans un condition déplo- déplorable chez elle. Que y n'avait jamais personne entrée, sauf une qui m'a fait, un petit peu, donner un signal de dire "écoute, au niveau sa maison c'est la catastrophe". #00:09:56-9# Et pis là, je l'ai mis chez elle, chez [pas compris]. Et puis elle a, elle a réussi- j'ai dis "écoute [pause]- pour moi elle est pas normale. J'ai impression qu'elle est un peu une, une euh, une sorte de, de, de, euh… De trouble de développement- inadéquate, mais je peux pas me prononcer exactement de quoi il s'agit, j'ai envoyé chez le neuro-psychologues. Qu'ils ont constaté qu'il y a une petite déficit [acquiescement de MH1: "hum"], mais pas franchement autant ce qu'elle néglige dans sa vie. Et pis que elle a une sorte une personnalité dépendante, un peu parce que, elle arrive suivre que une chose. Si on lui met deux choses à la fois par exemple au niveau de, euh, euh… Au Home [pas bien compris] elle doit gérer un petit peu au niveau d'animation, elle peut pas suivre deux gens à la fois. C'est que un". J'ai dis "mais pour moi c'est pas normal. Il y a, il y a une petite déficit- soit une négligence, soit un déficit, j'ai du mal à… [acquiescement de MH1: "hum"] À, à savoir en exact ce qui se passe". Je l'ai mis chez elle, et puis finalement elle a, elle a, elle a trouvé sa bonne diagnostique. #00:11:07-8# Et puis on a mis sous traitement, on avait réussi un petit peu l'intégrer où elle est. #00:11:13-3#

MH7: C'est quoi le diagnostique ? #00:11:14-3#

MF4: Hum, c'est de, de, des troubles bipolaires. #00:11:17-9#

MH7: C'est une bipolaire. #00:11:19-4#

MF4: Avec un touche schizo-affective euh- #00:11:23-9# [acquiescement d'MH7: "hum"] [pause] associé. Et puis là, finalement euh, un peu [souffle] parce qu'elle était encore une petite comprenante borderline [pas très bien compris], parce qu'elle, elle essaye de profiter du système aussi hein. #00:11:41-8#

MH7: Mais elle a tout là. [rires] #00:11:43-2#

MF4: Est-ce qu'on a fait un [inaudible]- #00:11:43-7#

[rires] #00:11:44-1#

MH5: Un peu affectif, un peu schizo… #00:11:46-7#

MF4: Oui. #00:11:47-4# [rires d'MH7] #00:11:47-2# Et puis là, elle se trouve en arrêt de travail. J'ai dis- #00:11:50-2#

MH5: C'est vaste le diagnostique [rires] #00:11:49-0#

[autres interactions entre MH5 et MH7 mais inaudible] #00:11:50-7#

MF4: -Ok, il a fait une petite chute mais sans aucunes importances. Elle vient avec des symptômes tout à fait bizarroïdes. Parce qu'elle veut profiter du système, qu'elle est en arrêt de travail. Je dis "qu'est-ce que vous arrivez à faire à la maison?" - j'arrive à tricoter, j'arrive à faire ça, ça, ça. J'ai dis "vous arrivez assez faire pour moi, vous avez de travail à 50%". J'ai envoyé chez elle et pis on a constaté la même chose [acquiescement de MH1: "hum"]. #00:12:15-4# On a mis au travail parce que il y a quand même une pathologie qui est derrière, qu'elle peut fonctionner avec cette pathologie-là. Mais finalement qu'elle veut aussi profiter dans une manière [acquiescement de MH1: "hum"]- du sytème. Et là si j'étais toute seule, je n'arrivais pas… Il faut avoir plus de temps pour éplucher des choses, et je ne suis pas capable d'aller jusqu'au bout pour des troubles de personnalité, troubles du caractère c'est des, des domaines que c'est très difficile savoir, nous comme généralistes. #00:12:51-7# Moi- pas moi, je sais pas, vous autres si vous êtes euh, capables d'aller jusqu'au bout diagnostique. #00:12:57-5# Mais je trouve très difficile. #00:13:01-0#

MH7: C'est dur de répondre comme ça dans une généralité quoi. #00:13:03-4#

MF4: Ouais. #00:13:04-1#

MH7: C'est… #00:13:05-9#

MH5 : Bon je sais si ça c'est- en tout cas ça représente un de mes cas emblématique, mais c'est un peu plus complexe là, mais… #00:13:09-4#

MF4: Oui, c'est, c'est- #00:13:10-9#

MH5 : En tout cas moi les, les emblématiques que j'ai eu c'était quand même plusieurs fois, plusieurs fois des burn-outs euh, états dépressifs euh, qui duraient, problèmes d'assurances, etc. où là elle a pu… #00:13:19-4#

MH7: Oh oui, oui, y a ça aussi. #00:13:21-1#

MH5: Y a aussi ça, y a tout, ouais. #00:13:22-6# [acquiescement de MF4: "ouais"] #00:13:23-4# Après y avait des situations [bruits de fond qui empêche la bonne compréhension] où on était vraiment bien, parce que c'était un peu à cheval. y a des personnes pour lequel il demandait EXIT [pas sur car bruit de fond]. Mais pour lequel on était pas sûr qu'il y ait, qu'il y ait une, une [réaction de MF4: "capacité"] -capacité de discernement, pis on venait juste me demander la capacité de discernement #00:13:35-4#, pis il avait une rigidité de l'esprit

sans diagnostiques psy euh, avéré mais pour lesquels du coup j'arrivais pas à me prononcer. Pis comme on était entre la psychiatrie et la médecine générale, bah c'était assez intéressant parce j'avais l'impression qu'autant moi de l'avoir l'avis de la psychiatre c'était super utile. Pis autant elle, elle est revenue plusieurs fois discuter avec moi pour- en mettant la limite de la norme [pas bien compris]. Et je pense que l'interaction- directe d'avoir les deux- je ne pense pas que si je l'avais envoyé à l'extérieur ça aurait donné le même résultat. #00:13:58-5# [acquiescement de MF4: "ouais"] #00:13:59-8# Et euh, on arrive à quelque chose où finalement c'est vrai qu'il était un peu- un peu spécial, avec une rigidité, une psycho-rigidité mais pas de diagnostic et n'empêche qu'en capacité de discernement [pas bien compris]. #00:14:08-7# Et là typiquement je trouvais vraiment utile parce que, je pense que la psychiatre ça avait aidé à poser un peu cette capacité de discernement par rapport à ce que je lui disais. Pis moi ça me rassurait parce que je voyais que y avait quand même quelque chose qui jouait pas #00:14:20-1# [acquiescement de MH1: "hum"] Mais, on, on était un peu sur cette limite, et d'a- d'avoir l'interaction c'était- j'ai [acquiescement de MH1: "hum hum"] trouvé vraiment bien. #00:14:25-2#

MF4: Et puis le deuxième cas, ce qui était intéressant pour moi c'est de, de jeunes, qu'ils arrivent pas à réussir au niveau ses examens. Et puis là on voit que… Il y a les troubles d'attention, et il y a une trouble euh… Ça veut dire euh, des angoisses qui se rajoutent. Mais- on sait jamais euh, quand dans c'est des troubles de l'attention et puis euh, une personnalité angoissée c'est un peu la même chose, c'est difficile à dire. Et là, je lui ai fait tous les tests qu'on fait pour chercher une sorte d'état [pas compris]. Euh, et pis même l'état [pas compris] et devant l'examen d'être comme ça c'est dur à savoir. #00:15:14-0# Et puis, j'ai, j'ai adressé chez elle qu'elle a, elle a pris la patiente. Elle est allée même avec elle à l'examen hein. #00:15:21-4# Euh, pour essayer de gérer ses crises d'angoisses devant l'examen… #00:15:26-0#

MH6: Elle, elle a fait l'examen avec elle ? [rires]

MF4: Elle est allée, oui, oui. Elle est allée, elle est pas entrée à l'examen mais… #00:15:30-4# elle a accompagné…

MH7: Elle l'a accompagnée jusque-là? #00:15:31-8#

MF4: Elle a accompagné, de gérer ses crises d'angoisse… #00:15:35-0#

MH7: Ah ouais! #00:15:35-7# [acquiescement de MH6: "ouais"]

MF4: …Pour faire son examen, parce que sinon il y avait, il y avait- elle pourrait louper complètement. Elle a du complètement faire l'année, mais si elle passait ses [acquiescement de MH7: " ah ouais"] examens seulement théoriques parce que la pratique elle est bonne… #00:15:46-1#

MH7: Je suis étonnée. #00:15:47-1#

MF4: … Elle, elle réussissait, elle réussissait. Elle est allée, je trouvais très sympa. #00:15:53-4# Elle a réussi cet examen, cette dame. Pis maintenant elle travaille avec elle euh… Pour

euh, profondément, pour, pour voir si c'est- une dépression, si c'est une [?? pas compris] Elle accompagne et puis euh… On a plutôt, on arrive à stabiliser avec un résultat qui est- qui est mal. #00:16:12-8#

MH6: En, en tout cas tu dis que c'est quelque chose que toi tu pourrais pas offrir. #00:16:17-7#

MF4: Non. Je pourrai pas lui offrir. #00:16:17-8#

MH6: Tu, te- tu peux pas accompagner, ta patiente… #00:16:21-6#

MF4: Même- je sais pas comment. #00:16:23-7#

MH5: Moi je savais pas que la CL-Psy pouvait. #00:16:24-7#

MF4 & Alaban: Hein ? #00:16:26-2#

MH5: Je savais pas que la CL-Psy elle pouvait faire ça mais… [acquiescement de MH7: "ouais"] C'est bien. #00:16:29-0#

MF4: Ouais, elle est, elle est allée avec elle à l'examen, carrément. #00:16:32-4# [acquiescement de MH7: "ouais"] Et pis elle a, elle a essayé euh, de donner des outils, gérer les crises d'angoisses jusqu'elle est rentrée. Elle a passé l'examen. #00:16:40-3#

MH1: Très bien. Est-ce que y a des fois que vous hésitez ? #00:16:44-4#

MH7: Oui. On hésite à transmettre- #00:16:46-9#

MH1: Oui.

[pause] #00:16:48-2#

MH5: Oui. #00:16:49-2#

MH1: Recourir alors. #00:16:49-6#

MH7: Oui. #00:16:51-3# [pause] Parce que comme elle est dans une durée limitée. [rires] Et que ben voilà, moi par exemple, je sais qu'elle va- ça fait deux mois que je sais qu'elle va s'en aller. Et que ça change, et que je me dis si on débute une prise en charge maintenant qui va s'arrêter directement derrière pour des gens qui hésitent. Alors ils ont- ils vont avoir de nouveau- ils démarrent et pis ça y est, ça change déjà. Alors ça c'est un, c'est un facteur limitant pour moi. #00:17:14-9#

MF4: Ouais. #00:17:15-5#

MH5: Et toi tu penses, hésiter par rapport à [acquiescement de MH1: "hum"] est-ce qu'on a raison de lui envoyer ou pas ou? Ou c'est égal ? #00:17:19-5#

MH1: Sans raison. #00:17:22-6#

MF4: Ça c'est- #00:17:23-5#

MH5: Moi j'me- moi les seuls fois où j'hésitais c'était plutôt pour pas la surcharger parce que j'ai l'impression que c'est une- [acquiescement de MH1: "hum hum"] une ressource tellement précieuse pour nous que je voulais juste essayer de maintenir euh… Un peu l'utiliser comme joker, donc des fois je me disais : [acquiescement de MH1: "hum"] "est-ce que ça vaut vraiment la peine que je lui envoie ou est-ce que, elle pourrait pas s'en charger ?" #00:17:37-8#

MH6: Ouais. Ou, ou, ou des fois j'hésitais de, de, de me dire "voilà là c'est une situation qui- qui nécessite probablement- un suivi plus longtemps [acquiescement de MF4: "oui"]. Et, et, et que je trouvais dommage que, que elle commence et pis après elle, elle doit, elle doit référer l- la situation [acquiescement de MH1: " hum hum"] #00:17:52-2#

MF4: Ça c'est- c'est sûr. Surtout que maintenant qu'on approche à partir. C'est ça. #00:17:56-4# [acquiescement de MH1: " hum hum"]

MH6: Euh, et, et puis aussi euh [pause] #00:18:02-0# Je sais pas, peut-être des situations où je trouverais bien qu'elle, qu, qu- qu'elle fait ça pas ici. Euh, euh le suivi avec le psychiatre, mais que elle, elle- la personne va peut-être dans euh. dans- euh, en ville ou… #00:18:15-7#

MH1: — situations ou ? #00:18:16-9#

MH6: Hein ? #00:18:17-1#

MH1: Cette situation ce serait quoi comme, comme cas ? #00:18:20-0#

[pause] #00:18:21-6#

MH6: Euh… [pause] #00:18:23-3# Ça peut être des, des, des problèmes de proximité aussi, que voilà de la patiente elle travaille en ville etc. et pis pour elle c'est peut-être plus facile aussi de, de, de, de consulter un psychiatre en ville, après le travail ou… Ou des choses comme ça. Ça c'était une fois le cas que… [acquiescement de MH1: " hum hum"] #00:18:40-6# Euh… #00:18:43-1#

MF4: C'est aussi ce qui est bien parce que le, le lien, l'empathie psychiatre et pis-[pause] euh patient. Moi je trouve que- soit elle aime son métier. Beaucoup. Parce qu'elle a beaucoup investie sur son métier et pis euh tous mes patients que je lui ai en tout cas adressé, ils sont, ils sont franchement contentes [acquiescement de MH1: " hum hum"] #00:19:07-5#

MH5: Mais qu'est-ce qui te fait hésiter ? C'est ça le truc. Qu'est-ce qui te ferait hésiter d'envoyer ? #00:19:10-6#

MF4: De hésiter- d’envoyer maintenant parce que on sait plus qui va venir hein. [acquiescement de MH1: “hum”] #00:19:16-1# [pause] On sait pas qui, qui vient. Et puis, euh… Si je vois que, une patient il demande une prise en charge qui va durer plus que deux-trois ans, dans ce cas-là, j’ad- j’adresserai pas chez quelqu’un qui va rester six moi ça c’est sur et certain. [acquiescement de MH1: “hum”] #00:19:36-9# Parce que de commencer à changer, c’est pas évident pour le patient. Ils disent. Ils disent #00:19:43-7#

MH5: Ceci dit, ça c’est un peu le propre de la psychiatre de liaison. Parce que- enfin tout cas moi dans ma conception de quelqu’un qui vient ici, c’est pas justement pour prendre pleins de patients à suivre pendant des années. Je trouve que surtout un intérêt de- [acquiescement de MF4: “ouais”] d’orienter, d’évaluer pis si jamais c’est des longs suivi référer. Donc c’est vrai que si… #00:19:58-1#

MF4: C’est ça. #00:19:58-3#

MH5: …D’emblée on sait que ça va être un besoin d’un long suivi. Moi je les envoie pas chez elle. Par contre- bon après y a souvent, elle peut elle aussi orienter. #00:20:05-6#

MH7: C’est ça aussi. Y a aussi ça. #00:20:06-7#

MH5: Mais après, elle sait chez qui l’envoyer… #00:20:08-1#

MF4: L’orienter #00:20:08-2#

MH5: …Comment, et de trouver des places. [acquiescement de MF4: “oui”]. #00:20:09-3#

MH7: moi j’en ai quelque-uns qu’elle a vu, elle a déjà organisé après le suivi #00:20:13-6#

MH5: Voilà. #00:20:13-5#

MH7: Pour aller un peu plus loin #00:20:14-9#

MH6: Avec la psychiatre hein, c’était avec- #00:20:15-2#

MH7: Ouais. Au CNP. Et pis un peu la facilité de dire “ben voilà, y a une partie qui est gérée”. Parce qu- pour moi le gros problème c’est souvent quand les gens disent “ouais j’aimerai bien un suivi, vous pouvez me trouver quelqu’un? #00:20:25-5# [acquiescement de Philipp: “oui. Vous, vous-”]. Je dis “je sais pas”. #00:20:27-8#

MH6: Vous avez eu des situations- [se racle la gorge] où- elle a référé après à un, à un psychologue ou un psychiatre privé ? #00:20:35-2# [acquiescement de MF4: “oui” ;acquiescement de MH7: “hum hum”] Pas, pas au CNP ? #00:20:36-3#

MH7: Pas au CNP ? #00:20:37-4#

MF4: Oui. #00:20:37-5#

MH6: T'as, t'as eu ça ? #00:20:38-5#

MF4: Oui. #00:20:39-0#

MH6: Ok. #00:20:40-5#

MH5: Moi j'ai pas souvenir. #00:20:42-7#

MH6: Moi j'ai, j'ai- j'ai pas non plus de souvenirs. Donc là, là, là j'ai deux situations que là euh, euh… Où, où, où y a un suivi après qu'est proposé au, au, au CNP [acquiescement de MH1: "hum hum"], au CNP ambulatoire finalement. #00:20:58-1#

MH5: Je pense que c'est le réseau qu'elle connait aussi le mieux. Je sais pas à quel point au CNP ils collaborent beaucoup avec des privés

MH6: Oui, oui, tout à fait. #00:21:02-7#

MH5: souvent des institutions, ben comme y a les outils, ils sont déjà à l'intérieur de l'institution. J'imagine qu'elle les connait bien. Pis c'est plus facile d'organiser ça, je suis pas sûr qu'elle ait les entrées beaucoup plus facile chez les- les psychiatres. #00:21:11-4#

MH7: Que nous, que nous. #00:21:12-5#

Ça doit être un peu comme nous. [acquiescement de MH7: "hum hum"]. #00:21:14-9#

MF4: Moi j'ai un jeune qui est adressé plutôt chez un psychiatre en ville euh. C'est un- il s'agit d'un schizophrène. [pause] #00:21:25-7# Pis là, il y a une psychiatre qui va la suivre parce que c'est pathologie chroni- chronique finalement. Ça c'est elle qui a dit "écoutez, moi je pense cette personne ça va être bon pour lui [acquiescement de MH1: "hum hum"], vu son profil". Parce qu'elle a vu une fois, deux fois. Je pense qu'avec cette personne ça va- il va bien se trouver pour pouvoir arriver permis de travail et pis avoir un résultat. #00:21:51-6#

MH5: À part ça je trouve qu'elle a bien gérer en tout cas cet aspect-là [acquiescement de MF4: "ouais"], parce que jusqu'à la fin elle a pu avoir des places. Moi une de mes craintes au début c'est qu'on lui refile toutes nos situations psychiatriques, pis qu'y ait- tout d'un coup bah, rapidement elle puisse nous dire: "ben moi j'ai plus de places, donc je suis déjà ces patients". Pis que- elle puisse plus avoir ce rôle qui nous nous est surtout utile de, d'orienter, de rapidement évaluer une situation [acquiescement de MF4: "hum hum"], de lancer des choses. [acquiescement de MH7: "hum hum"] Et pis moi je trouve qu'elle a bien fait [acquiescement de MF4: "c'est ça"], ouais c'et quelque chose qu'est pas si facile. #00:22:17-7#

MH6: Tout à fait. #00:22:18-2#

MH5: Et puis le risque c'est que, très vite en étant là qu'un jour par semaine, ben voilà en deux mois je suis plein [acquiescement de MF4: "hum hum"] et pis ben maintenant faut

envoyer ailleurs. Pis nous la plus value c'est justement qu'on puisse rapidement traiter ces situations. [acquiescement de MH7: "hum hum"] #00:22:26-8#

MF4: C'est ça. Comme les cas j'ai discuté, elle a mis déjà une infirmière qui est chez elle, les socio qui ont regardés, elle a organisé tout au niveau de l'hygiène- euh d'appartement et tout le reste. C'est elle qui l'a fait avec [pause] avec ces, ces groupes qu'elle peut mettre [acquiescement de MH1: "hum"]. #00:22:46-4# Ouais, je trouve que c'est pas [inaudible] #00:22:48-2#

MH1: Et le patient, et le patient il réagit comment ? Quand vous…? [pause] Quand vous invoquer la possibilité voilà de… #00:22:55-7#

MH6: Comment le patient [pas compris]- #00:22:57-7#

MH7: Qu'est-ce qui ont, ouais. #00:22:58-5#

MH1: Oui, quand vous invoquer la possibilité de, de, qu'il, qu'il voit un psychiatre. #00:23:02-3#

MH7: Ah bah, le, le, le… #00:23:03-5#

MH5: Donc le fait de voir un psychiatre chez nous ? #00:23:05-6#

MH1: Quand vous évoquer- #00:23:06-7#

MH7: On leur propose #00:23:07-7# [acquiescement de MH1: "oui"] Et comment est-ce qu'ils réalisent en général. [acquiescement de MH1: "oui"] Ouais. #00:23:09-0#

MH5: En général très bien. #00:23:11-2# [acquiescement de MH7 & MH6: "ouais"] #00:23:11-4# Parce que les quelques fois où je leur dis- je voulais leur favoriser en leur disant: "écoutez on a un psychiatre ici, je vais vous envoyer ici". Je leur dit toujours, je penserai bien, si jamais on en a une qu'est là euh- [acquiescement de MF4: "oui"] avec laquelle j'peux- je peux discuter. Systématiquement ça a toujours été: "ah bas alors très bien [acquiescement de MH7: "oui"] volontiers, alors si je peux venir voir, moi ça m'arranges de venir plutôt ici [acquiescement de MH7: "oui"]. Y a jamais personne qui m'as dit "ah ben alors non". #00:23:30-8#

MH7: Ouais pis leur réaction: "ah c'est ici en plus". #00:23:32-4#

MH5: Ouais. [inaudible] … un endroit connu #00:23:33-1#

MH7: Ah ok. C'était plutôt vraiment- "ouais c'est juste l'étage en dessus" - "ah [pas compris] #00:23:38-5#

MH5: Pis on sent tout de suite que y a une barrière un peu moindre. On doit moins se battre, ceux qu'on doit envoyer au CNP, à l'extérieur. Même s'ils nous le disent pas comme ça on voit que l'énergie pour les convaincre elle est souvent beaucoup plus grande. Là, plusieurs

fois quand j'ai dis que ce serait bien de voir un psychiatre, pis qu'on dit que y en a un ici, ça m'a prit étonnement peu de temps pour les convaincre. [acquiescement de MH7 & MH6: "ouais"] #00:23:56-4#

MH6: Et, et, et, combien de fois on a vécu dans notre consultation qu'on a donné trois, quatre adresses de psychiatres [acquiescement de MF4: "ouais"] aux patients. Et puis ils disaient "voilà ben c'est avec ces psychiatres j'ai déjà collaboré" [acquiescement de MH7 : "ouais"] #00:24:07-4# essayer de, de les appeler. Pis à la prochaine consultation les démarches n'ont pas été fait... #00:24:13-2#

MH7: Ouais pis ceux qui disent "j'ai mis un message sur le premier, j'ai mis un [acquiescement de MH6: "exactement"] message sur le deuxième. Et personne- personne ne répond. #00:24:18-8#

MH6: Personne m'a- personne m'a appelé. #00:24:20-8#

MH7: Donc y a carrément zéro réponses #00:24:21-9# Donc les gens ils font ça une fois, je comprends au bout d'un moment, vous êtes bien gentils mais vos adresses euh… #00:24:25-4# Donc là je veux dire quand les gens ont leur dit "he, vous avez rendez-vous ici" euh… #00:24:30-1#

MH6: Ouais- ouais ils, ils apprécient #00:24:31-2#

MH7: Ça c'est… #00:24:32-5#

MF4: Euh… #00:24:33-5#

MH7: Enfin moi j'ai toujours l'impression, ils connaissent le lieux, ils voient où c'est… Je veux dire y a pas encore une incertitude, déjà que faut aller voir un psy c'est déjà un peu [acquiescement de MF4: "hum hum"]. Et en plus dans un endroit on sait pas si c'est bien ou.. [pas compris] #00:24:43-7#

MH5: Aller à l'hôpital psychiatrique ou des choses comme ça. Si on les envoie au CNP [acquiescement de MH6: "ouais"] c'est tout de suite psychologiquement quand même difficile. #00:24:49-4#

MF4: Je pense déjà de pas téléphoner, parce qu'il y a cette sorte de culpabilité hein. "Je suis malade psychique" c'est un peu dur à avaler hein. Déjà de pas téléphoner au psychiatre, de dire "voilà je suis comme ça, comme ça" mais qu'il sorte avec la rendez-vous, je pense ils ont déjà 80%- relaxés. #00:25:09-8# Parce que souvent quand on dit "faut téléphoner au psychiatre" parce que là vous sentez un petit peu le lien comment il va être- une psychiatre et vous-même. Souvent il y a plusieurs qu'il hésite. Mais comment il va me répondre, comment je dois commencer à lui dire ce que, ce qu'il m'arrive. Je suis venu un peu fou euh… - ils ont cette, cette sorte de crainte. Après c'est vrai que [acquiescement de MH1: "hum hum"] tout le monde y peut avoir des hauts et des bas, que c'est pas pour les fous- quand il y a trop de choses qu'on arrive pas à gérer et puis… J'essaye toujours de dire que c'est la société fait un épuisement et puis, finalement on doit avoir quelques outils pour

savoir comment, on peut gérer mieux ces, ces, ces, ces choses-là. Il- c'est bon pour, pour faire les démarches. Mais quand c'est là, ils pensent qu'ils ont une seule confiance que- elle est dans le [pas compris], #00:26:02-2# on le connait, et pis qu'on envoie pas chez quelqu'un qu'ils méritent pas. C'est j'ai impression aussi. #00:26:09-9#

MH5: Bon après, pour l'aspect en tout cas du seuil pour, pour avoir des rendez-vous c'est vrai que c'était tellement plus simple. Moi j'ai pas fait longtemps finalement où j'ai pratiqué ici avant qu'elle arrive. Mais pour les quelques patients pour lesquels j'avais besoin d'un psychiatre, j'en ai trouvé zéro. [acquiescement de MF4: "ouais"]. Et c'est que quand elle est arrivé, tout d'un coup, ben hop, tous les patients [acquiescement de MH6: "ouais"] pour qui j'en avais besoin j'ai pu trouver. #00:26:27-4#

MH6: Et ça c'est vachement cool. #00:26:28-2#

MH5: J'arrivais vraiment pas à en trouver. Alors peut-être que j'étais débutant je connaissais pas très bien le réseau #00:26:31-1#. Mais c'était impossible de trouver un rendez-vous chez un psychiatre! #00:26:33-8#

MF4: Non mais je- même si tu connais le réseau, c'est pas évident. #00:26:36-3#

MH5: Et du coup, je sais- on sait juste pas, on en a besoin [pas bien compris car bruit de fond], on peut juste rien référer. Après, les seuls qu'on, qu'on sait où on peut avoir des rendez-vous c'est, c'est au CNP. Mais au CNP, y a des gens qui veulent pas y mettre les pieds. #00:26:45-3# Et en impossible de… #00:26:46-4#

MF4: Non, mais non. #00:26:47-6#

MH7: Ceux qu'on sait [inaudible] et on sait pourquoi. #00:26:48-0# [acquiescement de MH5: "ouais"]

MF4: Il y a deux mois de retard aussi. [acquiescement de MH5: "ouais, ouais"] Il y a deux mois de retard. Il y a beaucoup de gens qui partent. Euh… Je sais pas, quand ils m'ont dit "deux mois", moi j'ai téléphoné moi-même. #00:27:00-0#

MH5: Tandis que là, tout d'un coup t'as besoin. Tu peux au moins orienter… #00:27:02-0#

MF4: Deux mois de retard! #00:27:02-5#

MH5: …Discuter avec la psychiatre, #00:27:04-0#

MH6: Et puis- puis effectivement… #00:27:04-1#

MH5: et pis avoir des rendez-vous. #00:27:05-4#

MH6: Une situation, moi j'ai une patiente qui me consultait seulement quand elle allait pas bien. Et quand elle allait pas bien, elle s'enfermait chez elle. Et a plusieurs reprises, je lui ai proposé de, de-… euh un suivi psychiatrique, euh, juste parce qu'elle était un trouble

bipolaire [pas bien compris]. #00:27:21-9# Et finalement elle a pu accepter parce que c'est ici. De, de, de, de venir et pis de, de, de-… D'avoir un diagnostique précis, d'avoir une médication qu'elle prenait pas, qu'elle, qu'elle acceptait pas, euh… #00:27:35-7#

MH5: Hum-hum, hum-hum. Faut dire que que le- prendre un rendez-vous chez un psychiatre, c'est quand même différent de prendre un rendez-vous chez le cardiologue. Le monde psychiatrique fonctionne déjà différemment. [acquiescement de MF4: "ouais"] Déjà alors y a, y a toujours cet aspect que c'est aux, aux patients de faire le premier pas, parce que ça fait partie d'une démarche de choix. #00:27:46-7# Mais n'empêche que déjà ça, c'est une barrière quand même pour y aller. Parce que y en a qui, qui feront pas l'effort. [acquiescement de MH6: "hum"] Pis après justement, c'est difficile, si on envoie une lettre au cardiologue en lui demandant de convoquer un patient, il va le convoquer. Alors des fois y aura un peu de retard, mais il va le faire. Quan- quand eux ils appellent un psychiatre en demandant un rendez-vous, des fois y a pas de réponses, ou des fois ce sera juste "non" pis on en trouve nulle part. [réaction de MH6 : "Et-"] #00:28:04-6# Et on peut pas nous envoyer une lettre au psychiatre en disant: "merci de convoquer tel et tel pour telles raisons". Donc même pour nous c'est compliqué, la croix et la bannière pour euh, pour essayer d'avoir un rendez-vous. #00:28:13-1# [acquiescement de MH6: "ouais"] Tandis que si on demande à l'orthopédiste ou au neurologue, n'importe, on envoie une lettre, ils nous l'envoient pis des fois y a plus ou moins de délai, mais c'est tout. #00:28:18-7# Mais on aura notre rendez-vous. Tandis que là, des fois… Là c'est compliqué. #00:28:23-6# Tandis que là on a quelqu'un, on sait comment elle fonctionne, on sait comment lui demander. On lui envoie un email, on lui explique la situation, est-ce que tu es d'accord, pas d'accord. Si oui, t'as de la place quand ? Pis… C'est, c'est vraiment efficace. #00:28:35-9# [acquiescement de MH7: "Hum hum"]

MF4: Moi je trouve c'est un grand aide d'avoir un psychiatre dans un cabinet comme ça. #00:28:39-8#

MH1: Hum. Et, et pour la relation- et pour la relation entre vous et le patient. Quel est l'enjeu du fait de, de demander euh, une aide psychiatrique ? [Pause] Est-ce qu'il y a des enjeux ou? #00:28:57-9#

MH5: Tu dis par rapport de nouveau à elle en particulier ? Ou de manière générale à demander… ? #00:29:02-0#

MH1: Demander à elle, oui. #00:29:03-4#

MH5: Parce que là il y a clairement un enjeu, c'est que justement quand on- le, l'aspect psychiatrique, souvent les gens sont déjà réticents à y aller. [acquiescement de MH1: "hum hum"] Si ils vont chez quelqu'un pis qu'ils ont une mauvaise expérience on peut mettre la croix sur avoir un jour une aide parce que ils voudront plus [acquiescement de MF4: "oui, c'est ça"] y retourner, pour- dans beaucoup de situations. [acquiescement de MF4: "ouais"] Il y en a peu qui seront d'accord d'aller voir encore quelqu'un d'autre. Là, le grand enjeu, c'est de les envoyer chez quelqu'un chez qui on a confiance. [acquiescement de MF4: "ouais" ; acquiescement de MH7: "hum hum"] Et, et chez qui les patients savent qu'on a confiance. Et là du coup ben on la connait, on a parlé avec elle, on sait qu'à la limite elle elle est d'accord de les recevoir, que c'est une bonne indication. Donc là l'enjeu c'est qu'on

assure, à priori, beaucoup mieux que ça se passe bien. #00:29:34-6# Donc ça c'est un gros enjeu, tandis que si on doit juste leur dire, de leur donner une liste de psychiatre, trouvez-en un. L'enjeu c'est que y a pas de chances que ça se passe pas très bien. J'en ai eu un ou deux qu'était chez des gens et y remettront plus les pieds [acquiescement de MF4: "hum"]. Pis après ils viendront plus voir. Là on sait qu'on va chez quelqu'un qu'est bien, on a confiance. Pis, il y a beaucoup plus de chance que le suivi il aboutisse. #00:29:53-3#

MF4: Moi je trouve- ouais. #00:29:52-1# [acquiescement de MH6: "oui"]

MH5: Je trouve que c'est le principal des enjeux. #00:29:53-5#

MH6: Et, et le fait aussi- on peut presque les accompagner chez le psychiatre. On peut aller avec eux au rend- au premier rendez-vous [acquiescement de MH5: "hum hum"]. Quand ils sont réticents, on peut leur, leur, leur, leur prendre la peur, en, en disant "voilà, écoutez on va faire comme ça euh, on fixe le rendez-vous chez elle et je fixe dans mon agenda, et je serai le premier quart d'heure avec vous" #00:30:12-5# [acquiescement de MH5 et MF4: "hum hum"] #00:30:15-2# Et, et j'ai fais ça une fois. Et, et, ça s'est très bien passé. Je trouve c'est- on peut donner des informations, et pis… L'adhérence [pas compris] c'est assez bien. #00:30:25-0#

MH5: Pis, pis, la reconnaissance pour nous, tu disais l'enjeu relationnel pour nous avec le patient [acquiescement de MH1: "hum"]. Si on les a envoyé chez quelqu'un et qu'effectivement ça se passe bien, déjà qu'on a du des fois un petit peu les travailler au corps pour qu'ils aillent voir le psychiatre. Si après ça se passe bien, ben ça f- ça fait- ben voilà ça établie une confiance entre nous et le patient. Quand on les envoie pis ils nous disent "mais vous m'avez envoyé chez machin, mais c'était la catastrophe, on m'a prit de haut, ça s'est mal passé". Bah, pour nous aussi, on a notre relation avec le patient c'est aussi un petit peu délétère, parce qu'on l'a forcé à faire un truc qui lui nous avait déjà dit qui voulait pas, pis après ben vous avez vu le docteur ça s'est mal passé. Ben là, on sait que si on les envoie ici, pis qu'on la connait, pis qu'on a confiance. Moi cette situation je l'ai jamais eu. #00:31:00-3#

MF4: Moi ce que je pense qui est positif d'avoir une psychiatre ici, parce qu'on lui transmets pas mal de… [pause] De données pour notre patient. Et puis des psychiatres, déjà, il sait de quoi il s'agit. Il peut aussi s'orienter dans les questions assez précis [acquiescement de MH1: "hum hum"], et ça met un lien direct, un empathie direct avec le psychiatre. Quand on, on voit ailleurs ils savent absolument rien de quoi il s'agit. #00:31:27-5#

MH5: Ça aussi d'ailleurs c'est assez curieux par rapport au cardiologue. [acquiescement de MF4: "ouais"] Le cardiologue, la première chose qu'il va faire, c'est qu'il va nous demander des informations, la liste [acquiescement de MF4: "ouais"] des antécédents, des traitements etc. parce qu'il a pas envie de tout recommencer. Tandis que… Le psychiatre, en général. Moi j'ai jamais un psychiatre qui m'a demandé… [inaudible] #00:31:39-2#

MH6: Il veut rien- il veut rien savoir de nous. #00:31:41-7#

MH5: Il veut rien savoir, pis il veut rien nous dire. #00:31:42-6#

MH6: Et, et pis il. il, il va rien nous dire. [rires] #00:31:43-7# Il demande des fois un ECG, [acquiescement de MH5: “voilà”] ou, ou un bilan sanguin. #00:31:48-8# [acquiescement de MH5: “ouais”] #00:31:48-0#

MF4: un ECG pour couter [pas bien compris]- #00:31:49-5#

MH5: Il y a le patient interposé. [pas bien compris] #00:31:51-4#

MF4: Et là je pense c’est la, la pourquoi va bien ici avec elle parce qu’elle a déjà toutes les renseignements avant qu’elle voit le patient. Moi je lui parle toujours quand je lui envoie des patients. [acquiescement de MH5: “hum”] Et elle sait attaquer dans les points sensibles. Et je pense c’est là qui marche, que l’empathie elle s’installe assez vite. #00:32:10-9#

MH5: Pis pour les patients de, d’aller voir un psychiatre qui est déjà un peu au courant, qui doit pas tout recommencer, tout re- [acquiescement de MF4: “hum hum”] prendre, parce qu’on- voilà il vient pour une raison précise, je pense c’est aussi sécurisant. #00:32:21-7#

MH7: Je sais pas ce que vous avez fait, mais vous avez fait des rapports pour l’adresser en général ? #00:32:24-9#

MH6: J’ai fait un petit mail, des, des fois. #00:32:26-3# [acquiescement de MH7: “ouais”] #00:32:27-1#

MF4: Moi je lui parle de comme ça. #00:32:29-1#

MH7: Ouais. Alors moi je lui faisais quand même une demande à une consultation. #00:32:32-2# Merci de la recevoir pour [acquiescement de MH5: “ouais”] telles ou telles raisons. Elle est- elle a tels problèmes somatiques, je lui faisais une petite lettre comme on lui fait d’habitude. [acquiescement de MH6: “ouais”] Juste pour qu’elle ait, un petit- voilà #00:32:41-1#

MH6: Oui, oui, un petit. #00:32:42-7#

MH7: Mais euh… #00:32:43-3#

MF4: Moi je parlais plutôt dans les problèmes psychiatriques. Ça veut dire la sensibilité, le point sensible. Comment elle est, l’hésitation de voir un psychiatre et tout ça. Et puis ce que moi j’ai remarqué- pis elle, elle a pu plus facilement entrer dans un relation de confiance parce que le patient elle, elle sa- elle- ça veut dire le psychiatre, elle a dit “mais elle me posait des questions, comme elle a vécu ce que j’ai vécu”. #00:33:13-0# Elle savait pas que je lui donnait toutes les, les données en avant pour euh, que euh- et c’est un petit peu ce que- ce qu’elle va attendre du psychiatre, du côté à elle [acquiescement de MH1: “hum hum”] pour créer cette sorte d’empathie [acquiescement de MH1: “c’est ça”] . Mais jusqu’à là j’ai jamais eu quelqu’un qui a, elle a, elle a vu une fois et pis qu’elle a plus voulu aller. [pause] J’ai jamais eu quelqu’un…#00:33:34-0#

MH1: Et du coup si je rebondis à ça. Quels sont- pourquoi vous pensez que ça se passe comme ça? Quels sont les bénéfices pour le patient, selon vous ? #00:33:43-2# D'avoir un psychiatre ici ?

MF4: La proximité, la confiance euh… Le, le résultat. #00:33:52-7#

MH1: Ok. #00:33:54-0#

MH5: Une collaboration d'aides soignants [pas sur] #00:33:55-9#

MF4: La collaboration, la prise en charge euh- adéquate et pis rapide. Sans que ces troubles- parce que si elle doit chercher encore deux mois des psychiatres, et ben, elle arrive plus dormir et pis euh, elle s'aggrave dans ces, ces, problématiques qui va arriver [pas compris] ça va être beaucoup plus dur à récupérer. Je pense tout ça c'est des points positives hein. #00:34:16-2# [acquiescement de tous les autres tour à tour: "Hum hum"] #00:34:17-3#

MH6: Et- je pense aussi euh, peut-être euh, l'aspect de… On, on va [pause] au cabinet [pause] donc on est, on est, on, on- chez, chez quelqu'un avec qui on peut parler donc on est pas fou. [acquiescement de MH1: "hum"] On doit pas aller au centre euh, euh, ambulatoire de psychiatrie en ville ou, ou, ou, ou dans un hôpital psychiatrique euh, ou peut-être c- cette crainte de, de diminuer ça, je pense ça joue un, un, un grand rôle. [acquiescement de MH1: "hum hum"] #00:34:50-9#

MF4: C'est beaucoup plus facile de venir dans un cabinet comme ça et pis aller voir un psychiatre parce que, souvent quand il va dans les centres psychiatriques neuchâtelois, Neuchâtel. Souvent les gens ils regardent qui est derrière eux, est-ce qu'ils les voient. C'est la crainte… #00:35:07-9#

MH5: C'est vrai que c'est moins…

MF4: Les, les gens. #00:35:09-0#

MH5: ..stigmatisant de venir au cabinet médical que d'aller à l'hôpital psychiatrique #00:35:11-3#

MF4: Et là c'est le cabinet médical, elle doit aller voir un psychiatre. Mais au cabinet médical, moi je suis à côté d'elle, en face d'elle où elle consulte. Personne ne sait si c'est pour moi ou si pour le psychiatre [acquiescement de MH5: "hum hum"] #00:35:22-9#

MH7: Et pis y a rien d'écrit sur la porte. #00:35:25-3#

MF4: Y a rien écrit sur la porte non plus. #00:35:27-0#

MH7: Pas marqué "consultation" #00:35:27-6#

MF4: Et je pense ça c'est- aussi ça fait que les gens ils consultent plus vite. Les difficultés qu'on a, moi ce que je trouve plus de difficultés avec les personnes âgées hein, les personnes

de, de soixante cinq ans qui font de, des dépressions un peu cachées post-retraites. #00:35:45-2# Euh, ils ont, ils ont honte de dire que- ils deviennent fou. Pour elle ils sont devenus fous hein. Pis là, déjà de parler pis d'adresser vers elle, j'ai dis “écoutez, elle est, elle est comme moi, comme un médecin, seulement elle va voir ce qui va moins bien maintenant que vous êtes à la retraite” #00:36:05-2# Là ils acceptent beaucoup plus facile. [acquiescement de MH1: “hum”] Ils acceptent beaucoup plus facile. #00:36:09-0#

MH5: C'est vrai que y a tout un pan de la psychiatrie qui irait jamais voir un psychiatre à l'hôpital ou à- même à l'extérieur. #00:36:15-9# [acquiescement de MF4: “mais ouais”] On a aussi été plus bas ici [pas bien compris], pis- qu'est pas forcément des trucs complètement banaux, anodins, y a quand même des situations [acquiescement de MH7: “ouais”], y a quand même plus ou moins complexes. #00:36:23-6#

MH6: Le, les généralistes ils traitent [acquiescement de MH5: “ouais”] 95% des, des, des cas psychiatriques, c'est ce qu'il dit. #00:36:30-6#

MH7: C'est ce que dit S., quatre-vingt- il disait quatre-vingt. #00:36:31-1#

MH6: Oui, quatre-vingt. #00:36:31-7#

MH7: Quatre-vingt. #00:36:32-5#

MF4: Moi je t'assure si… #00:36:33-9#

MH7: Je sais pas ce que ça donne les chiffres. [rires] #00:36:34-4#

MF4: si j'ai du adresser ces, ces patients soixante-cinq ans au centre psychiatrique, oublie hein. [acquiescement de MH1: “hum”] #00:36:41-0#

[pause] #00:36:43-0#

MH5: Pis après, pour certains patients, ça permet aussi des fois, ne serait-ce qu'une orientation. #00:36:47-0# Sans, sans qu'ils doivent forcément le psychiatre, parce que en ayant- finalement bon ben ça c'est l'expérience qu'on a, avait déjà ici à la, la PMU. Mais en ayant des portes de supervisions à distance [acquiescement de MF4: “ouais”], y a des gens qui sont très réticents à aller voir un psychiatre. Y a quand même une expertise psychiatrique qui peut être fournie [pause]. Via le généraliste parce que là on peut lui poser des questions, superviser, orienter pis de temps en temps ça évite aussi d'aller pour ceux qui veulent vraiment pas- #00:37:08-6#

MH6: Est-ce que- #00:37:09-7#

MH5: De quand même de pallier à ce genre de situations. #00:37:10-7#

MH6: Et-ce que vous, vous avez discuter des situations euh… #00:37:15-3# Qu'elle n'a pas vu ? Des, des, des- de… #00:37:17-3#

MF4: Oui. #00:37:17-7#

MH6: Sur- t- toi t'as pu discuter ? #00:37:18-9#

MF4: Moi j'ai pu discuter, et ce que j'ai appris #00:37:20-0#

MH7: En supervision, quoi. #00:37:22-4#

MH6: En supervision, oui. #00:37:22-8#

MH5: Ouais, moi, moi pas beaucoup, mais une ou deux fois. [acquiescement de MH7: "hum hum"; acquiescement de MH6: "ouais"] #00:37:25-1# Sur les six mois c'était, c'était très peu parce- #00:37:28-6#

MH6: Moi c'était le mardi, j'étais pas euh, j'ai eu congé l'après-midi, donc ça me, ça me coupe un petit peu pis… [acquiescement de MH5: "hum hum"] #00:37:33-4#

MF4: Moi ce que j'ai apprécié aussi de sa part, parce que j'avais une, une- c'est des cas qu'on discutait hein. Euh… Elle était pas encore diagnostiqué, on savait pas de quoi il s'agissait et pis elle avait une expertise psychiatrique très vite par son assureur. Et puis, je suis désolée mais l'expertise psychiatrique c'est toujours en faveur d'assureur sans qu'ils épluchent franchement le, le, le bonnes… Bonnes faveurs. Et puis là elle a réagit auprès de médecins de la caisse, [acquiescement de MH1: "hum hum"] euh… Que, cette expertise psychiatrique, pour moi c'est pas du tout une expertise. Il s'agit pas du tout d'un cas comme il décrit. Elle a donné ses côtés à elle et pis ils ont retournés en arrière. Ça veut dire… #00:38:25-9#

MH5: Pour qu'on ait eu plus de, peut-être de supervisions ou de [acquiescement de MF4: "ouais"] de ces interactions-là il aurait fallu qu'on ait plus de plages d'échanges un peu plus formalisées [acquiescement de MF4: "ouais"]. C'est vrai que moi j'avais la chance d'être là tous les mardis, pis surtout les mardis à midi pis c'est souvent un peu de manière comme ça officieuse [acquiescement de MH6: "ouais"] que j'échangeais. #00:38:37-5# Si t'es pas là à ce moment-là, c'est vrai que on avait pas de plage vraiment. On pouvait les réserver. Y a une fois où j'ai bloqué une plage dans son agenda pour qu'on puisse parler d'une situation parce que justement le monsieur avec la fin de vie, moi je savais pas trop. Enfin il demandait EXIT et les capacités de discernement. La- l'indication au bilan psychiatrique était pas si claire pour moi. Pis là on a pu en discuter, mais j'avais bloqué dans son agenda et ça je l'ai fait qu'une fois. #00:38:58-6# Sinon c'était toujours un petit peu comme ça… #00:39:00-3#

MH7: Un peu informel #00:39:00-8#

MH5: …à midi, un peu informel, mais si t'es pas là. Peut-être là si vraiment on avait voulu ce-cette place-là il aurait fallu qu'on, qu'on l'agende plus… #00:39:06-2#

MF4: C'est vrai - #00:39:08-6#

MF4 et MH5 (ensemble): maintenant #00:39:08-9#

MF4: Ouais. #00:39:09-4#

MH7: Bien que ce soit des patients précis #00:39:10-4#

MF4: Quand l'agenda il va être géré par le CNP, c'est beaucoup plus difficile de bloquer les plages hein. [acquiescement de MH1: "hum hum"] #00:39:16-3#

MH7: C'est à présent ce que les patients apprécient, c'est qu'on se connaissent. Qui ait, enfin voilà, souvent moi ça m'arrive avec d'autres patients externes. "Eh, vous connaissez Docteur Machin? [acquiescement de MH1: "hum hum"] Vous avez un contact avec lui ?" - "Non j'ai rien du tout" - "Pis il vous donne des rapports ?" - "Non rien du tout". Enfin voilà y a pas- [acquiescement de MH1: "hum hum"] donc le fait que ici. Pis ben voilà on a discuté avec la doctoresse euh, de, de, dossier. Enfin voilà on a parlé un petit peu, parce que vous étiez d'accord etc. Et pis ça y- c'est, [acquiescement de MH1: "hum"] ce lien ils aiment que- souvent, ils aiment qu'on se parlent quoi. #00:39:47-4#

MH5: C'est une extension aussi de ta propre confiance. Si ils ont confiance en toi, pis que toi t'a confiance en le médecin [acquiescement de MH7: "ouais"] eux ils auront aussi confiance [acquiescement de MH7: "oui"] plus facilement [acquiescement de MH7: "ouais, ouais"] en le médecin. #00:39:54-3#

MH7: Et que souvent euh, je veux dire [quelqu'un tousse, pas sur d'avoir compris] on a peur #00:39:56-1# que, que, y ait une- des patients, les patients sont contents de cet échange. [acquiescement de MF4 et MH6: "oui"] #00:39:59-7#

MH6: Parce que- #00:40:00-6#

MH7: Sauf, sauf situation exceptionnelle où vraiment t'as des gens qui veulent absolument pas que. #00:40:04-9#

MH5: Qu'ils veulent privé. #00:40:05-7#

MH7: Qui veulent tout séparer [acquiescement de MH5: "ouais hein"] le privé, mais… Sinon la plupart du temps c'est comme les autres. #00:40:09-1# [acquiescement de MH5: "hum hum"] On nous a fait un rapport une fois, il nous a dit euh… #00:40:11-8#

MH6: Ah oui. Ils nous demande aussi si le cardiologue nous a fait le rapport ou si le [acquiescement de MH7: "hum hum"] dermatologue nous a écrit. Il, il demande aussi. Et est-ce que vous l'avez reçu ce rapport ? #00:40:21-5#

MH1: Et, et concrètement, pour, pour le rapport, pour revenir un peu plus précisément, ou elle vous fait quoi comme retour ? Concrètement, c'est, c'est par quelles manières ? #00:40:30-2#

MF4: Pas de rapport complet. Parce que je pense- #00:40:32-2#

MH1: Oui, mais c'est comment ? #00:40:33-3#

MH5: T'as eu un rapport écrit une fois ? #00:40:34-2#

MF4: Non. #00:40:35-3# Du côté psychiatrique, je trouve que euh, même moi je suis généraliste hein et pis ça m'arrive de demander mon rapport de mon patient à l'hôpital. Ils m'ont dit "non je- on peut pas vous donner." - "Ok, je dois faire quoi alors comme démarches ?". "Le patient il doit lui-même signer un document. Que il permet de vous donner les renseignements à vous là on vous, on vous donne la"- #00:41:02-2#

MH1: Oui. Mais est-ce qu'il y a un retour par téléphone, de face à face ? #00:41:05-4#

MH6: ah, elle, elle m'a écrit deux, trois fois. #00:41:06-8#

MF4: Par face à face. #00:41:06-9#

MH1: ou mail ? #00:41:08-4#

MF4: Oui. #00:41:08-9#

MH6: Oui. #00:41:09-4#

MH1: Oui ? #00:41:10-0#

MH5: Je pense qu'elle… #00:41:11-2#

MF4: Email, face à face. #00:41:12-1#

MH5: …Elle a du établir un peu des modalités de, de communication un peu différentes en fonction de nous pis de quand on était là. Parce que moi elle m'a jamais écrit parce qu'elle sait qu'on se croise justement le mardi [acquiescement de MF4: "hum hum"], donc elle m'a toujours un peu parlé comme ça, on a trouvé un petit moment un midi pis elle m'a fait des retours pratiquement que comme ça. #00:41:25-6# [acquiescement de : "oui"] #00:41:26-2# Les seules fois où j'ai eu des rapports c'était justement plutôt être en copie du rapport qu'elle a écrit à l'AI, mais on a pas eu de retours formalisés. #00:41:32-4#

MH6: Elle l'a fait deux, trois fois des mails pour- même avec dans le même mail les, les, les deux, trois situations qu'elle suivait à ce moment-là [acquiescement de MH5: "hum hum"] euh, et pis… Ça c'était je trouvais incroyable de, de, de recevoir et pis de, de- faire copier-coller, pis on met dans le dossier [acquiescement de MH1: "hum hum"] pis… #00:41:51-3#

MH7: Moi elle m'en a parlé. #00:41:52-6#

MF4: Oui, c'est ça je pense pour nous elle… #00:41:54-5#

MH1: [inaudible] …de voix vive comme ça. #00:41:55-2#

MH7: voilà de voix vive, de vive voix, ouais. #00:41:56-6#

MH5: Mais ce qui, ben voilà, moi me convient bien déjà d'avoir un retour des psychiatres, c'est déjà précieux pour nous [acquiescement de MH1: "hum hum"]. #00:42:03-4# même si on a pas une lettre formalisée… #00:42:05-2#

MH6: Ouais, elle a, elle a, elle a su s'adapter à, à [acquiescement de MH5: "ouais"], à… À nos différentes manières de fonctionner. [acquiescement de MH1: "hum hum"] #00:42:09-4#

MH5: Après de temps en temps je pense que ce serait quand même pratique parce que justement ben là elle va partir donc, dès le moment où y a une assurance, dans six mois qui nous repose la question, qu'est-ce qu'il y avait, le diagnostique psychiatrique etc. Ben malheureusement des fois on aura pas de truc formel qui reprend les, les détails. C'est peut-être mon seul regret dans cette forme de communication. Pour gérer les situations ça va très bien. Par contre c'est vrai que ben après il reste quand même rien. Au moment où elle part, ben on, on a plus. Pis y a quand même souvent, où on a besoin pour les questions de rapport, de l'AI dans deux ans, n'importe quoi, de reprendre des diagnostiques, et ce qui avait été fait. Là ben c'est un peu plus délicat parce qu'on a.. #00:42:38-8#

MF4: On a toujours son numéro privé hein. #00:42:41-0#

MH5: On a pas, on a rien d'écrit. #00:42:41-7#

MF4: On a toujours son numéro privé. #00:42:44-3#

MH5: Ouais… Pis on se souvient pas toujours de ce qu'on avait discuté y a deux ans. [rires de MH6] #00:42:47-1# Pis les diagnostiques précis qui avait été évoqués à l'époque. On a une idée générale mais…

MF4: Mais normalement elle doit avoir euh… #00:42:52-4#

MH5: Quand il faut mettre le code… [pas compris] #00:42:53-9#

MF4: Pour chaque patient dans son dossier normalement après… #00:42:57-2#

MH5: Dans son dossier à elle, ouais. #00:42:58-6#

MF4: Oui, oui, oui. #00:43:00-1#

MH5: Ouais… Alors pour la retrouver. #00:43:00-4#

MH1: Et vous quand vous l'appeler, vous, vous- ? C'est, c'est comment ? Vous avez déjà parlé un peu, mais comment vous- faites les demandes ? De quelles manières ? Par téléphone ? Par mail ? Par… En oral ou…? #00:43:12-4#

MH6: Alors si elle est dans la maison, on lui donne un coup de fil. #00:43:15-5#

MH1: Appeler, ouais. #00:43:17-5#

MH6: Et, et, et, et… Ou on toque à sa porte. #00:43:19-7# [acquiescement de MH1: “hum hum”]. [pause] Et si c’est le jour elle est pas là, on, on.. #00:43:24-7#

MF4: Si elle est pas là… #00:43:24-8#

MH6: On fait un mail. #00:43:25-6#

MF4: C’est mail, ouais. #00:43:27-5#

MH7: Ça dépend le type de demandes. [acquiescement de MH1: “hum hum”] Y a des demandes, suivant les cas, non c’est vrai, si c’est dès que j’ai une demande plu- plus détaillée, j’écris. Comme ça je me les- après c’est juste une- une petite commentaire ben je sais que je vais la croiser ici le matin. Si c’est euh, juste une appréciation c’est un peu, ce- c’est variable. C’est entre la demande directe et pis un mail. #00:43:46-0#

MH5: Ce qui est d’ailleurs assez sympa cette souplesse, parce que des fois ben… Y en aimerait juste une petite orientation, une petite idée, ou ne serait-ce que lui demander si ça vaut la peine qu’on l’adre- qu’on lui adresse. Pis quand on doit faire une demande tout à fait formelle avec une grande lettre qui nous prend quand même au minimum 10-15 minutes voire plus quand il faut bien détailler. Quand c’est juste pour une orientation on hésite un peu, tandis que là quand c’est comme ça ben [acquiescement de MH7: “ouais”] on- plutôt on lui en parle, pis c’est un truc plus compliqué où l’on sait que de toute façon on aura besoin d’elle, ben on sait que ça vaut au moins la peine d’investir euh, 20 minutes [pas bien compris car bruit de fond] #00:44:11-9# à faire une bonne lettre pour qu’elle la lise. [acquiescement de MH1: “hum hum”] Si c’est à l’extérieur on est obligé de passer par une bonne lettre [acquiescement de MH7: “ouais”] Et pas .. [pas compris la fin] #00:44:19-6#

MH1: Et est-ce que vous arriver à… À regarder quel autour de vos questionnements, vos hésitations, vos, vos vécus finalement, vos inquiétudes avec le patient ? #00:44:29-8#

MH6: Oui. Ouais, ouais, ouais. #00:44:30-9#

MF4: Oui. #00:44:32-4#

MH5: Parfait. #00:44:33-5# [acquiescement de MH1: “hum”]

MF4: Même, même il était présent dans un colloque, vous voulez souvenez hein ? #00:44:37-7#

MH7: Hum hum. #00:44:38-8#

MF4: Quand il est intervenu hein [pause]. On était même avec nos secrétaires. C’était au niveau comment il faut gérer un peu les conflits pendant les cas qui vient, tu te souviens, il y a quelques temps ? #00:44:50-6#

MH7: Oui tout à fait, elle était intervenue. #00:44:51-6#

MF4: Elle est intervenue et pis ça on a trouvé c'est très… #00:44:57-9#

MH5: Je sais plus où c'était. #00:44:58-2# [acquiescement de MH7: "ouais"]

MF4: Très bien de sa part. #00:44:59-5# [pause] C'était au questionnement…

MH7: Ah! Elle [pas compris] au débriefing. #00:45:02-9#

MF4: Oui, aussi, de deébri- [peu audible] #00:45:03-6#

MH7: Elle a parlé du débriefing, on était en train de discuter de situations, elle a dit mais- #00:45:08-5#

MF4: Ça veut dire surtout les patients qui étaient agressifs avec les secrétaires [acquiescement de MH7: "ouais"]. Comment- on doit débri- débriefer, par quels moyens pour que nos secrétaires elles se protègent euh… #00:45:18-8# Du harcèlement. #00:45:20-0#

MH5: Elle était là par hasard, on avait une réunion avec les assistantes pis on faisait une euh… #00:45:22-3#

MF4: Oui, oui c'est ça. #00:45:23-0#

MH5: Une séance avec des débriefings. Pis elle était là pis elle est intervenue. Mais, de manière générale, moi je trouve qu'on a quand même pas eu énormément de moments vraiment- d'échanges, qu'elle- on pouvait lui parler des situations qu'on voulait lui adresser, j'ai quand même pas si souvent eu l'occasion après alors, après elle me retransmettait les informations importantes. Mais d'aller plus loin comme de temps en temps on avait l'occasion- pis nous on va un peu plus aussi, parler de comment nous on se sent dans la relation [acquiescement de MH6 : "ouais"], aller un petit peu plus loin sur le… #00:45:44-4# Ça y a-

MH6: On, on, on avait moins #00:45:45-2# de supervision.

MH5: Ben parce que voilà y a moins ce côté supervision. Y avait pas tellement de places alors ça… #00:45:50-8#

MH7: On l'a pas sollicité. #00:45:51-4# Après on-… #00:45:52-1#

MH5: Non c'est vrai. #00:45:52-2#

MH7: …C'était prévu hein. #00:45:53-2# On payait chaque mois pour, pour ça. #00:45:55-2#

MH5: On pouvait le demander [rires de MH6] mais fallait le demander, l'agender.. [rire de MF4] [pas sûre car rires trop fort] #00:45:57-5#

MH7: On payait une heure par, par semaine, [acquiescement de MH5: “ouais”] non? #00:46:00-7#

MF4: Parce que nous… #00:46:01-5#

MH7: Oui, par semaine! #00:46:01-6#

MF4: Nous c’est comme le patient. On, on met le patient en première ligne, et nous, pour nous… #00:46:06-1#

MH5: Ouais c’est ça. [rires de MF4] #00:46:07-0# Mais il manquait peut être un tout petit peu cette, cette plage de, [acquiescement de MF4: “ouais”] où on était ensemble pour le faire. #00:46:12-5# [acquiescement de MH6: “ouais”] Mais c’est vrai, peut-être qu’il aurait fallu formellement le demander, en disant “ben j’ai envie d’être supervisé pour telle situation, pis…” de lui bloquer, ça, ça prend du temps quand même on faisait ça. [acquiescement de MH6: “ouais, ouais”] #00:46:22-5# À la PMU on venait des fois toquer à la porte quand on voyait que c’était ouvert pis on savait que vous prendriez souvent le temps où bien vous nous diriez quand vous auriez le temps. Là c’est- y a un peu moins de disponibilité quand même donc euh... #00:46:31-7#

MH7: Bon. Y a, y a aussi le fait que c’était une mise en route hein. Vous aviez les premier six mois. #00:46:35-7# [acquiescement de MH5: “ouais” ; acquiescement de MF4: “ouais”]. Donc euh… Le, le temps qu’on voit comment ça fonctionne, comment ça se passe, de prendre des rendez-vous etc. Je pense que peut-être dans un deuxième temps… #00:46:44-8#

MH5: Peut-être petit à petit on fonctionnera un peu différemment. #00:46:46-5#

MH7: Gentiment on… Tiens tiens, cette situation, ça vaudrait vraiment la peine qu’on prenne le temps. Moi j’ai l’impression que y a un petit bout de ça. Au fur et à mesure j’ai vu comment est-ce qu’on arriverait mieux à- #00:46:57-4#

MH5: Mais bon, le… #00:46:58-8#

MH7: À profiter de ça. #00:46:59-3#

MH5: Y a quand même la question de la disponibilité parce qu’elle est là qu’un jour par semaine. [acquiescement de MH6: “ouais”] Donc déjà de voir tous nos patients si en plus elle doit superviser euh, cinq médecins pour leur ressenti dans leurs relations etc. #00:47:07-6#

MF4: Ouais ça [pas compris] #00:47:07-9#

MH5: C’est, c’est beaucoup de boulot hein donc euh… #00:47:10-3# [acquiescement de MF4: “ouais”] Après je sais pas à quel point. #00:47:13-6#

MF4: Par contre, ouais. #00:47:14-9#

MH7: Ouais. Bon en attendant y en a aucuns qui l'a vraiment fait. #00:47:16-0# Alors tu vois, entre, entre tous… #00:47:17-2#

MH5: On aurait pu bloquer, on aurait pu faire un planning. #00:47:19-0# Avec des tournus. #00:47:19-6# [rires] Tous les mardis c'est la supervision de… De Kraënbhul… [réaction de rires mais pas compris] #00:47:22-3#

MF4: Moi elle m'a fait hein. Moi elle m'a fait une fois parce que… #00:47:26-9#

MH5: [pas compris] sur le canapé, qui va parler de ses problèmes. #00:47:29-0#

MF4: J'avais un patient très bizarre, qu'il me parlait que pour l'esprit. J'ai dis, "mais moi je, je sais pas dans quelle manière je dois gérer les situations. Est-ce que je dois entrer dans son jeu? Est-ce que je dois lui dire que c'est pas du tout vrai ce qu'il pense?". Et pis là elle est venue avec moi. C'est moi que j'ai, j'ai géré toutes les consultations. Et pis, après euh… #00:47:51-8#

MH7: Elle est venue avec toi dans la consultation ? #00:47:53-5#

MF4: Oui, oui, oui. #00:47:54-0#

MH7: Ah, ouais. #00:47:54-5#

MF4: On a fait une consultation ensemble. #00:47:55-8#

MH7: Ah, ok. #00:47:56-5#

MH6: Donc elle était, elle, elle, était ? #00:47:58-2#

MH7: À quel endroit ? #00:47:59-0#

MF4: Elle était à côté. #00:47:59-3#

MH7: De qui ? De toi ou du patient ? #00:48:00-8#

MF4: Le patient il était là. [acquiescement de MH7: "ouais"] Moi j'étais là, elle était là. #00:48:04-8#

MH6: Ah, excellent. #00:48:05-4#

MH7: Ok. #00:48:06-1#

MF4: Et pis on a fait ensemble cette consultation. #00:48:08-9# J'ai dis pour moi "est-ce qu'il est schizophrène ? Est-ce que il est d'autres choses ? Je sais pas, mais il délire à mon avis parce que c- je sais pas. Après j'ai, j'ai, j'ai fait toute mon anamnèse je… Elle est intervenue

deux, trois fois sur trois, quatre questions précis, pour qu'elle se situe un peu de quoi il s'agit. Non, ça on a fait. #00:48:31-8#

MH6: Ouais, ouais le problème #00:48:32-8#

MF4: Et pis pour les deuxièmes qu'on a eu après… #00:48:35-2#

MH5: Après je pense que toi t'as eu peut-être un peu plus de… Peut-être un peu plus d'occasions parce que t'étais- y a beaucoup enfin moi j- j'étais là de temps en temps le mardi à midi, mais le mardi à midi toi t'étais quasi toujours là [acquiescement de MF4: “ouais”]. Donc elle- enfin je voyais, vous êtes presque tous les mardis midis tu lui parlais de tes situations. Donc en fait t'avais une sorte de plage peut-être… #00:48:47-8#

MH7: Avec la supervision euh… #00:48:48-8#

MF4: J'ai pas mal de psycho-social, finalement. #00:48:49-4#

MH5: Ouais, mais t'as pu parler de… #00:48:50-3#

MH7: Permanente [inaudible] #00:48:51-2#

MH5: …Beaucoup de situations comme ça, qui se sont fait par, par des questions de proximité. #00:48:54-3#

MF4: J'avais, j'avais beaucoup de psycho-social et puis c'était surtout… [pas compris] qui nous a épuisé tout le monde hein [rires] #00:49:00-0#

MH5: Mais est-ce que y a des fois où tu l'as contacté en dehors de ces plages un peu informelles à midi, où vraiment tu lui dis à dit “j'aimerai être supervisé pour telles situations” ? #00:49:06-7#

MF4: Oui, mais pour ça je lui ai dis: “écoute, moi j…” #00:49:08-9#

MH5: Oui mais tu lui as dis pas juste quand tu t'es croisé à midi ? #00:49:10-0#

MF4: Non, non. #00:49:10-9#

MH5: Tu l'as appelé pour ça… #00:49:11-7#

MF4: Non, non, non. #00:49:11-7#

MH5: Ou tu as écrit.. #00:49:12-5#

MF4: Non, non, non. #00:49:13-3#

MH5: …Par email pour lui demander ça ? #00:49:13-4#

MF4: On a organisé en avance, on a organisé en avance, on a organisé deux semaines avant. #00:49:16-0# [acquiescement de MH1: “hum hum”] Qu’on la voit le patiente, le patient ensemble. #00:49:19-8# [pause] Non, non j’ai organisé deux semaines. #00:49:23-6#

MH1: Et, et globalement, vous avez l’idée que cette collaboration elle modifie votre regard clinique ? Votre manière d’intervenir ou ? De quelles manières? Est-ce que la collaboration avec le psychiatre sur place est-ce qu’il modifie votre regard clinique ? #00:49:37-8#

MH5: Moi clairement puisque d’habitude quand ils vont voir le psychiatre j- je sais pas ce qu’il en pense, ou je sais pas ce qu’il fait donc plutôt plus je peux lui faire confiance et puis m’en occuper. Là quand même, d’avoir un retour, de connaitre les diagnostiques, de connaitre un peu l’évolution, savoir ce qu’en pense le psychiatre, moi ça m’aide à m’orienter quand même dans ma manière d’être… #00:49:52-0#

MF4: Mais c’est normal, oui. #00:49:53-5#

MH5: Si on sait que y a un trouble qui est plus ou moins avéré ben on sait un petit peu mieux comment, comment anticiper les causes ou les attaquer. Si on sait où en sont les diagnostiques, si on sait qu’on peut décharger déjà une partie assez curologique [pas sur]. Moi je trouve que ça modifie, ça modifie pas mal de choses dans ma manière d’être. #00:50:08-7#

MF4: Ouais. #00:50:11-2#

MH7: Le regard clinique. #00:50:12-3#

MH5: Mais le regard clinique… #00:50:13-1#

MH7: Je but avec ça moi. Qu’est-ce que ça change dans le regard clinique, c’est-à-dire est que je perçois autrement ? #00:50:18-3#

MF4: Est-ce que tu arrives mieux euh, se situer dans les cas que on a facilement, on a pu louper… #00:50:24-3#

MH7: Attends, attends, attends, excuse. Parce que… #00:50:25-3#

MF4: C’est ça regard clinique. #00:50:26-4#

MH7: Ouais, ouais. Non je… [pause] Je, j’arrive pas, ce, ce…- C’est abstrait pour moi. Pour l’instant… [pas compris la fin] #00:50:34-4# [acquiescement de MH1: “hum”] [rires] #00:50:35-6#

Phillippe: Dans, dans, dans la façon de faire… #00:50:38-0#

MH1: Oui. #00:50:38-8#

MH6: Peut-être ? #00:50:39-8#

MH1: Comment ça se traduit dans vos interventions. Est-ce que vos interventions elles sont modifiées un bout, du fait qu'il y a le psy de liaison qui intervient aussi ? Ou qui vous fait un retour, je sais pas ? #00:50:52-8#

MH6: Alors on a, on a un outil de plus. #00:50:55-6#

MF4: En hôpital psychiatrique. #00:50:57-1#

MH6: C'est surtout ça. #00:50:58-0# Après si on l'utilise ou pas, ça c'est, c'est en discutant avec le patient. Mais ça nous donne quand même un, un, un outil de plus, de, de- et pis de, de j'ai l'impression aussi un petit peu de l'assurance. [acquiescement de MH1 "hum"] #00:51:10-4#

MH7: Et ouais, c'est, c'est comme si y a un truc qu'était posé ailleurs, et pis que ça, ça change forcément le… [acquiescement de MH1: "hum hum"] #00:51:15-6#
Je pense à deux situations, où ben voilà, il y a un partie qui est gérée, et pis ça change forcément, j'en sais rien, je sais pas comment dire. [acquiescement de MH1: "hum hum"] Quand je revois la personne, eh bah… C'est bon il y a un problème qu'on peut mettre de côté. #00:51:30-5# [acquiescement de MH6: "oui"]

MF4: Oui, ça décharge, ça décharge. #00:51:31-4#

MH7: Et que- tout d'un coup, on peut aborder autrement. Parce que sinon la personne elle a, elle a envie d'être entendue. Sauf que, on l'a entend pas forcément, on a pas le temps et on doit gérer autre chose en même temps. Donc là c'est tout d'un coup y a une partie qu'on peut clairement poser de côté et il faut encore qu'on règle cette question-là. #00:51:46-5# Donc ça enlève- oui ça change la clinique [acquiescement de MH1: "hum hum"], mais ça c'est sur. #00:51:51-2# J'ai aussi une autre situation où voilà, une situation conflictuelle de couple, tout d'un coup cet aspect il a été- alors évidemment, je vois beaucoup [pas compris], peut-être que j'ai d'autres patients qui ont des psychiatres à l'extérieur, mais comme, comme je suis plus au clair de ce qui se passe là. Je le vois, je le sais, [acquiescement de MH1: "hum"] et pis c'est- donc je, je réalise en tout cas dans ces cas-là, que ces questions-là, ils sont, ils sont ju- ils sont suivis et soutenus ailleurs. #00:52:15-9#

MH6: Et tu te sens moins seul. #00:52:17-0#

MH7: Et tu te sens moins seul, oui. Exactement. #00:52:18-5# En tout cas ça se perçoit euh, ouais. #00:52:20-5# [acquiescement de MH6: "ouais"]

MH1: Oui, et dans le, dans le vécu si vous… [pause] #00:52:25-0# Partez de le vécu, dans le vécu des situations complexes, alors est-ce qu- à quoi ce sens dans [pas bien compris] votre vécu finalement ? On parlait de la solitude, est-ce qu'il y a d'autres vécus, qui sont différents du fait qu'il y a un psychiatre qui est là ? #00:52:37-2# [pause] Dans les situations complexes ? #00:52:39-0#

MF4: D'abord, quand on est sur que euh… Il est chez un professionnel, qui va la aider plus qu'on peut lui offrir… #00:52:48-1#

MH5: Contrairement quand il est chez nous. #00:52:48-7#

MF4: Hein? #00:52:49-3#

[Rires de MH7 et MH6] #00:52:50-0#

MF4: Quoi ? #00:52:51-3#

MH6: Tu te dévalorises! #00:52:51-9#

MH7: C'est que en fait chez nous, il- c'est pas très professionnel. #00:52:55-2#

MF4: Non, non. #00:52:56-0#

MH7: C'est ça que tu veux dire ? [rires] #00:52:57-1#

MF4: J'ai dis pour les cas comme, comme dans, comme dans la médecine chez nous, souvent on fait des choses, on sait plus de quoi il s'agit hein. [acquiescement de MH1: "hum"] #00:53:03-5# Et pis on cherche l'aide ailleurs. Mais là, j'ai dis quand chez- c'est un psychiatre, au moins on sait que au niveau diagnostique, elle va aller jusqu'au bout. On a un peu plus soulagé de ce côté-là. Euh, moi je parle pour moi hein. Et puis… #00:53:21-5#

MH6: Bon c'est pas si simple. Tu demandes deux psychiatres et pis t'auras quand même deux diagnostiques différents. [acquiescement de MF4: "ouais"] Je... [rires] #00:53:27-2#

MF4: Mais au moins, on est pas fautives. Parce que on a pas mis une diagnostique que, euh… #00:53:36-4# [petite pause] Que c'est- c'est parce qu'il lui, ce qu'il lui appartient à lui directement, selon ces symptômes-là [acquiescement de MH1: "hum"] #00:53:44-0# C'est ça.

MH7: Et que ça permet de la sécurité. #00:53:45-7#

MF4: On, on donne- ça améliore la sécurité [pas bien compris] et puis, cette sphère-là, on sait qu'elle est gérée autrement. Deux, quand on a une diagnostique précis, si on se trouve devant un schizophrène par exemple, on sait que des fois quelles- questions on doit poser si ils viennent avec une sorte de délire et d'agressivité, parce que on doit aussi savoir où la porte elle se trouve hein. [rires] #00:54:08-7# Voilà, il y a… #00:54:12-3#. [pause] On, on se- moi je, je me comporte autrement quand je sais que je me trouve avec un schizophrène devant moi. [acquiescement de MH1: "hum hum"] #00:54:22-0# Au niveau de… #00:54:22-0#

MH5: C'est vrai quand on a une sécurité de diagnostique, un petit peu plus… #00:54:23-5#

MF4: De poser les questions, et pour le reste. #00:54:24-8#

MH5: …de certitudes, c’est aussi plus facile, d’al- de, d’aller, enfin d’y aller plus franchement, où [acquiescement de MF4: “ouais”] on sait qu’on est un peu plus en confiance. Donc c’est vrai que moi je rejoins surtout MH6 de- ben voilà c’est pour ça souvent qu’on s’entoure de spécialistes. C’est que les situations sont complexes. Pis d’avoir quelqu’un qui connait bien, ça, ça oriente, pis on est moins seuls, dans des situations des fois c’est compliqué de s’en sortir. [acquiescement de MF4: “oui”]. #00:54:42-2#

MH7: Ouais, où tu te dis, je rate quelque chose [acquiescement de MH5: “ouais, ouais”], est-ce que je vois pas quelque chose ? [acquiescement de MH5: “ouais, ouais… Ouais.”] Rien que le fait qu’il y ait eu un regard de quelqu’un [acquiescement de MH5: “ouais”] qui est avisé [acquiescement de MH5: “ouais”], ben ça te détend un peu de te dire “bon ben voilà on est déjà deux à avoir regardé la situation” [acquiescement de MH5: “ouais”]. #00:54:53-0#

MF4: C’est ça. #00:54:53-8#

MH5: Et pis quelqu’un qui conrsacre aussi le temps. Parce que comme tu disais ben c’est vrai que souvent… #00:54:58-1#

MH6: D’être tout seul dans la pièce [pas bien compris] #00:54:59-0#

MH5: Y a tout pleins de plaintes, pleins de choses qui se- quand y a de problèmes psy, y a des somatisations, d’autres choses. Ben c’est vrai que de tout gérer en parallèle, c’est pas toujours facile- ou d’y accorder le temps nécessaire. #00:55:07-2# Quand on sait que y a une partie, ben qui est- le psychiatre va prendre le temps de… D’a- d’aborder l’aspect psychiatrique ben nous, ça nous permet des fois de [acquiescement de MH7: “hum hum”] situations où y a trop d’aspects… #00:55:15-9#

MH7: Pouvoir alléger ce qu’on fait. #00:55:16-4#

MH5: …De pouvoir aussi se focaliser sur d’autres choses.

MH6: Oui. Et pis, de, de, de savoir que ben finalement on est pas tout seul [acquiescement de MH5: “hum hum”] à porter la situation, la dame qui se croit être à la maison, qui, qui sort jamais qui euh, etc. On, on sait que voilà, elle a encore un rendez-vous là. Elle est- elle prend, elle prend la médication et pis on peut euh.. On, on- quand elle vient chez moi, on peut au moins discuter qu’est-ce qui se passe chez le psychiatre [rires] #00:55:41-2# Euh… Donc c’est quelque chose qui, qui, qui…[acquiescement de MF4: “ouais”] #00:55:45-3# nous arrive [pas bien compris] #00:55:45-4# Quelque chose..

MH1: [inaudible] #00:55:46-1#

MH7: Je vous mets juste en garde il est 13h20. [acquiescement de MH1: “hum, hum”] C’est juste que dans [acquiescement de MH1: “hum, hum”]- je sais pas, c’était… #00:55:48-9#

MF4: Est-ce que on est au bout des questions ? #00:55:50-8#

MH7: Non, [rires] non. #00:55:51-0#

MH1: Presque, presque. #00:55:51-9#

MH5: Y avait peut-être encore un dernier aspect, [acquiescement de MH1: "oui"] c'était le, le côté aussi de, temps en temps quand y a des situations qui sont un peu conflictuelles avec des patients. Ben y a des fois c'est difficile de savoir si c'est nous qui devons nous remette en question dans notre attitude ou bien si c'est le patient. Pis là, d'avoir quand même des fois quelqu'un qui est… Qui est, qui est assez sensible à ce genre de choses et qui nous donne un deuxième avis, ça nous aide aussi ben tout d'un coup à nous rassurer en disant "non mais je suis quand même juste dans mon attitude, mon appréciation c'est pas juste moi qui me laisse déborder par mes à priori, mes attitudes". Pis là d'avoir ça, c'est un [pas compris] #00:56:19-0#

MH6: De savoir… #00:56:19-7#

MH5: C'est confortable. #00:56:20-6#

MH6: De savoir- parce que on se pose des fois la question "est-ce que nous on est adéquate ?" Est-ce que nous, avec notre [acquiescement de MH5: "ouais"] fonction de, de… #00:56:26-0#

MH5: Avec notre compétence. #00:56:27-1#

MH6: De, de percepter [?? pas compris] des choses acquiescement de MH5: "ouais"] avec notre fonctionnement, avec no- comment on est avec acquiescement de MH5: "ouais"] le patient, est-ce qu'on est adéquat. Et pis d'avoir un retour par euh, par, par le, le [acquiescement de MH5: "hum hum"] psychiatre ça, ça, ça nous rassure [acquiescement de MH5: "hum hum"] et pis ça nous… #00:56:38-4#

MH1: Ok. Comme le temps file, mais peut-être par rapport le projet plus global. Euh… Quels sont les bénéfices, si on peut dire comme ça, si on essaye d'être un peu- les bénéfices du projet, c'est quoi ? #00:56:52-7#

MF4: Je pense tout ce qu'on a discuté [acquiescement de MH1: "hum hum"] c'était bénéfique. [acquiescement de MH1: "hum hum"] #00:56:54-8# On a rien d'autres à ajouter de plus. #00:56:57-2#

MH7: Ben je sais pas. On est plus efficaces, [acquiescement de MH1: "hum hum"], on est plus pertinents [acquiescement de MF4: "hum hum"] euh, je pense que… C'est plus rassurant pour nous. C'est plus rassurant pour les patients. [acquiescement de MH1: "hum"] #00:57:09-3# Ben je pense que- voilà.

MF4: On perd moins de temps pour chercher de droite à gauche. #00:57:11-5#

MH7: Ensuite, je pense qu'on arrive à rendre la psychiatrie moins… Moins… #00:57:14-9#

MH5: Elitiste [pas bien compris] #00:57:16-6#

MH7: Hein ? #00:57:17-0#

MH5: Ouais. #00:57:17-7#

MH7: Moins, moins- voilà c'est pas un truc à part. Ça, ça inclut un peu plus partout c'est tellement sectorisé, on a tellement pas [pas bien compris à cause du bruit de fond] d'énergie à, à préparer la psychiatrie, je crois que c'est vraiment une façon de la, de la rendre… #00:57:30-4#

MH5: Communautaire. #00:57:32-0#

MH7: Communautaire quoi. C'est une, c'est une spécialité comme une autre quoi. C'est, c'est toujours cette question-là. Y a toujours des centres à part… Pour l'instant ça change #00:57:38-8#

MH5: Ou moins stigmatisé. #00:57:40-2#

MH7: C'est moins stigmatisé, voilà. Je cherchais le truc. #00:57:41-7# [pause] Moi je trouve que c'est, c'est- ça part dans la même démarche que on a mit un CUP [???] [acquiescement de MH5: "hum hum"]. Euh, au sein de l'hôpital physique. #00:57:50-5#

MF4: C'est ça. #00:57:51-1#

MH7: Parce que avant il fallait choisir l'hôpital, soit on avait des urgences de ce genre, soit des urgences de ce genre. Donc on était déjà- il fallait vraiment- ouais je trouve que déjà que les gens aillent aux mêmes urgences. Après qu'on soit pas dans le même bureau, mais c'est les urgences. Parce qu'on a un problème de santé, ça veut dire même- c'est de la santé aussi. Et pis finalement [acquiescement de MH1: "hum"], ça part du même registre-là. C'est qu'on, on va au cabinet parce que on a un problème de santé. Alors après y a le bureau qu'est plutôt-là etc. Mais c'est pas euh, euh deux trucs complètement séparés. Ça- je pense que c'est plutôt une démocratisation de la psychiatrie, de la rendre plus… #00:58:22-1#

MH5: C'est moins isolé [acquiescement de MH7: "ouais"] pour la psychiatrie aussi. #00:58:25-1#

MH6: De, de l'intégrer, dans notre… #00:58:26-3#

MH7: De l'intégrer. #00:58:27-1#

MH6: …Quotidien. Dans notre, dans, dans nos attitudes, dans nos… Dans… Parce qu'on a tous, tous de ça. On a tous, des, des idées bizarres hein. [rires] #00:58:34-2#

MH5: Certains un peu plus que d'autres. #00:58:36-7#

MF4: D'avoir moins cette phobie des psychiatres. #00:58:37-0#

MH7: Ouais, tout à fait. Je pense que c'est vraiment le… #00:58:40-3# [pause] Le, le, le… Ouais, c'est cette stimgmatisation qu'on essaye de, d'améliorer quoi. #00:58:48-5#

MH1: Et les aspects négatifs ? #00:58:49-9# [pause] #00:58:52-3# Est-ce que y a des points négatifs de ce projet ? #00:58:53-3#

MH7: Oui, il y a des points négatifs. #00:58:56-0#

MH1: Moins positifs. #00:58:56-5#

MH7: [rires] Moins positifs c'est qu'on est pas sur de pouvoir le prolonger. #00:58:59-2# [Rires de MH6 et MF4] #00:58:59-9#

MH5: [inaudible] #00:59:02-1#

MH7: Ça on en a pas de garantie. #00:59:02-6#

MF4: On a pas de garantie c'est ça. #00:59:04-8#

MH7: Et quand on dit "ah lalala, c'est embêtant parce que c'était bien" Et que on est… #00:59:08-2#

MH6: On doit de nouveau faire tout seul [rires] #00:59:09-7# … Tu fais ça… [?? pas sur]

MH5: C'est vrai que y a des, y a des choses qu'on pourrait améliorer dans le fonctionnement, mais c'est pas des points négatifs sur le principe du projet. Y a que des avantages moi je trouve. [acquiescement de MF4: "ouais"] #00:59:17-9#

MH6: Ouais, idéalement, ce sera chouette si elle est là deux jours par semaines. #00:59:21-3#

MH5: Alors après, les, les aménagements de disponibilité, de plages… #00:59:23-9#

MH1: Ça serait quoi ? Les choses d'amélioration, de développement futur, des idées de développement ? #00:59:28-0#

MH5: Ah, ben moi y a peut-être quand même deux, trois des fois d'avoir un peu plus de plages et de formaliser pour les supervisions, pour les plages… #00:59:32-8#

MF4: Oui, c'est ça. #00:59:33-3#

MH5: …D'échanges. Y avait le fait d'avoir des rapports. D'avoir une trace [acquiescement de MH7 ou MH6: "ouais"] écrite quand même un petit peu… #00:59:37-2#

MH7 ou MH6: Le rapport. #00:59:37-2#

MH5: …Avec des diagnostiques, des choses comme ça. Moi c'est les deux choses que [acquiescement de MH6: "ouais, hum hum"] que c- mais, mais justement qui sont des choses que simplement, c'est vrai que si ça se prolongeait, on - petit à petit on mettrait en place. Donc c'est pas le principe du projet même, c'est juste que dans notre manière de collaborer, c'est des ajustements. #00:59:50-6#

MH6: Et, et, et l'accessibilité de son agenda, je trouvais. Au début c'était très bien avec les… [pas compris] qu'on voyait que.. #00:59:56-5#

MF4: Avec nous, oui. #00:59:56-9#

MH6: Ses plages vides, avec un clic. Et pis maintenant qu'on doit euh, orienter le patient à… À nos secrétaires qui, qui cherchent un rendez-vous. #01:00:06-3#

MH7: On pourrait faire. #01:00:07-6#

MH6: On pourrait faire, mais c'est… #01:00:08-7#

MH7: Mais on le fait pour pleins de choses, mais on en a raz-le-bol de flanquer [rire de MH6] des pommes dans tous les coins, qu'on se souvent jamais. #01:00:13-9#

MF4: Oui, il faut… #01:00:15-0#

MH1: D'autres réflexions d'améliorations ? De… développement ? Sans toucher à à la réalité existantes mais… ? #01:00:21-4#

MH5: Effectivement le, le l'occurence de pérennisation du projet. [acquiescement de MH1: "hum hum"]. #01:00:27-9#

[Pause] #01:00:30-4#

MH1: Pourquoi ? Qu'est-ce qui serait comme… Le regret ? #01:00:34-5#

MH5: Ben parce qu'on voit que c'est utile. Pis c'est vrai que… #01:00:38-5#

MH6. Ouais, ça sert. #01:00:38-9#

MH5: On, on débloque tout un mode de fonctionner avec cette collaboration-là. Pis si on l'a plus, on sait qu'on devra retrouver d'autres manières, qu'on, qu'on sait plus de nouveau vers qui les adresser après on va retrouver des clichés. #01:00:49-2#

MH1: Mais est-ce qu'il vous aurait besoin peut-être d'heures supplémentaires ? #01:00:52-9# Pour les généralistes, pour les médecins généralistes, des aspects psychologiques, de formation, de type spécifique d'intervention. Un autre mode de collaboration ? Qu'est-ce que vous pensez à d'autres ? #01:01:04-6#

MF4: Mais là il y a pas mal aussi de formations qui se font dans le sphère psychiatrique. Il y a des, des formations purement psychiatriques des fois quand on reçoit l'invitation. Euh… Moi quand je vois le thème qui m'intéresse je vais aussi et je suis gé- généraliste et je vais là-bas. Ça veut dire maintenant est-ce qu'un va faire un office encore une formation en plus. Probablement, probablement avec les psychiatres qui va être là de nous présenter des fois des cas [acquiescement de MH1: "hum hum"], pourquoi pas, ça peut… Nous donner un peu un aspect différent au niveau, au niveau de nos attitudes quotidien avec les cas psychiatriques. Pourquoi pas, d'avoir de temps en temps une sorte présentation qu'on fait nous hein. #01:01:54-9#

MH5: Après, moi j'ai l'impression que l'offre de formation en termes de, enfin de, de théories qu'on a, y a déjà assez de choses… #01:02:00-2#

MF4: C'est ça. #01:02:00-2#

MH5: Enfin je trouve. Après où ça aide vraiment dans ce genre de projet, c'est le côté plus supervision- #01:02:04-2#

MF4: C'est ça. #01:02:04-9#

MH5: D'avoir une supervision. Alors, après je pense qu'on peut tous à titre indépendant euh, organiser comme, avec la médecine psycho-somatique où t'as une supervision qu'est déjà mise en place. [acquiescement de MF4: "hum hum"] Pis ça c'est chouette. Mais là ben du coup on en a une partie qui vient au cabinet un peu spontanément. [acquiescement de MF4: "hum hum"] #01:02:16-6#

MH7: Donc, j'imagine que si ça se prolonge, on aurait peut-être pus aussi faire des- avec les assistantes #01:02:22-1#

MH5: Alors après… #01:02:22-6#

MH7: Par exemple faire euh- enfin on se réunit de temps à autres pur des- enfin, [acquiescement de MH5 : "hum hum"] pour des colloques de formation, alors on choisit des thèmes qui sont sur le grill là comme ça. [acquiescement de MH5: "hum hum"] #01:02:31-8#

MH6: Mais c'est vrai que ça, on pourrait… #01:02:33-3#

MH7: Ça pourrait être utilisé, pis qu'on fasse un jour avec elle par exemple [acquiescement de MH5: "hum hum"], de dire "tiens, ben on parle de- qu'est-ce qui, qu'est-ce qui vous créer problèmes [acquiescement de MH1: "hum hum"] avec des patients psy ? #01:02:43-0#

MH6: Le patient difficile à la réception. #01:02:45-3# [acquiescement de MF4: "ouais"] Un petit peu comme ça [inaudible] #01:02:46-6#

MH5: [pas bien compris] aussi. C'est ça, dans le relationnel dans le… Dans le vécu aussi des relations, y a des choses qu'elle juge très frustrantes, dans leur quotidien, de, de refuser des offres avec des pa- enfin quand il y a pas de place pour les patients, de devoir dire "non", des

gens qui les agressent.-Donc, y a, y aurait pleins de places c'est vrai où, elle elle bénéficierait d'avoir une forme de supervision. #01:03:04-3# [acquiescement de MH7: "hum"] #01:03:05-5#

MH6: Ouais. C'est peut-être même qu'on peut… #01:03:09-2#

MH5: [rire] #01:03:10-6# Tiens, on va, on va demander. #01:03:12-0#

MH6. Ça serait une bonne idée. #01:03:12-8# [acquiescement de MH5: "ouais"] Peut-être leur dire "voilà, euh. On utilise aussi cette supervision qu'elle nous offre pour, euh.. Collaboratrice". #01:03:20-9#

MH5: Pis sachant que elle, elle travaille ici. Parce que c'est, c'est pas comme si on demande par exemple, ben maintenant on va avoir Monsieur- le Docteur S., pis on lui demande d'un jour [pas compris car bruit de fond] …existante ce sera pas la même chose que CL-Psy qui travaille au quotidien avec elle, qui sait un petit peu comment elle fonctionne. #01:03:32-7#

MH7: Qui passe à la maison, qui voit concrètement comme ça se passe. #01:03:33-3#

MH5: Qui passe à la réception, qui voit concrètement qu'on sait. Qui peut- ouais. Ça serait beaucoup plus pertinent. #01:03:38-9#

MH7: Et l'ambiance, ouais. [pas sur] [acquiescement de MH5: "ouais"] #01:03:40-2#

MH7: Excellent. #01:03:41-3#

MH5: Ouais c'est une bonne idée ça. #01:03:41-6#

MH7: Excellent, ouais. #01:03:42-4#

MF4: Qu'elle- elle veut regarder ceux qui pètent les plombs plus facilement de #01:03:46-4# [rire de MH6] dire "ça c'est- il est au bout, il faut…" [rires] #01:03:48-9#

MH5: Ouais, savoir comment gérer, comment les [inaudible], comment se comporter. #01:03:50-8#

MF4: Je sais pas. [rires] #01:03:51-6# Il faut intervenir. #01:03:56-2#

MH1: Très bien. On arrive à la fin.
